# Robust Physics‐Informed Neural Network Approach for Estimating Heterogeneous Elastic Properties from Noisy Displacement Data

**DOI:** 10.1002/advs.202508445

**Published:** 2025-11-25

**Authors:** Tatthapong Srikitrungruang, Sina Aghaee Dabaghan Fard, Matthew Lemon, Jaesung Lee, Yuxiao Zhou

**Affiliations:** ^1^ Wm Michael Barnes '64 Department of Industrial and Systems Engineering Texas A&M University College Station TX 77843 USA; ^2^ J. Mike Walker '66 Department of Mechanical Engineering Texas A&M University College Station TX 77843 USA

**Keywords:** elastography, inverse problem, mechanical properties estimation, noisy measurement data, physic‐informed neural network

## Abstract

Accurately estimating spatially heterogeneous elasticity parameters, particularly Young's modulus and Poisson's ratio, from noisy displacement measurements remains a significant challenge in inverse elasticity problems. Existing inverse estimation techniques are often limited by instability, high noise sensitivity, and difficulties in recovering the absolute scale of Young's modulus. This work presents a novel Inverse Elasticity Physics‐Informed Neural Network (IE‐PINN) to robustly reconstruct heterogeneous elasticity distributions from noisy displacement data based on the principles of linear elasticity. The IE‐PINN incorporates three distinct neural network architectures, each dedicated to modeling displacement fields, strain fields, and elasticity distributions. This approach significantly enhances stability and accuracy under measurement noise. Additionally, a two‐phase estimation strategy is proposed: the first phase recovers relative spatial distributions of Young's modulus and Poisson's ratio, while the second phase calibrates the absolute scale of Young's modulus using boundary loading conditions. Methodological innovations, including positional encoding, sine activation functions, and a sequential pretraining strategy, further improve the model's performance and robustness. Extensive numerical experiments demonstrate that IE‐PINN effectively overcomes critical limitations faced by existing methods, providing accurate absolute‐scale elasticity estimations even under severe noise conditions. This advancement holds substantial potential for clinical imaging diagnostics and mechanical characterization, where measurements typically encounter substantial noise.

## Graphical abstract

Heterogeneous distributions of elasticity parameters are learned from noisy displacement data using the Inverse Elasticity Physics‐Informed Neural Network (IE‐PINN). This approach integrates both data‐driven and physics‐driven methods to address the significant limitations faced by existing techniques. The proposed framework is particularly advantageous for clinical imaging diagnostics and mechanical characterization, especially in scenarios where measurement errors are present.

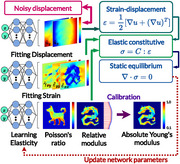

## 1 Introduction

Quantifying the spatial distributions of elastic properties, specifically Young's modulus and Poisson's ratio, is crucial in numerous applications, including biomedical imaging. Young's modulus characterizes a material's local resistance to elastic (reversible) axial deformation, while Poisson's ratio quantifies the coupling between axial and transverse deformations. When external loads are applied, an internal stress field is established within the solid material, resulting in deformation (i.e., displacement) patterns that depend directly on spatial distributions of the elastic properties. Accurate spatial characterization of elastic properties is essential for precise disease diagnosis, including cardiovascular and airway diseases,^[^ ^1^, ^2^, ^3^, ^4^, ^5^ ^]^ as well as cancer.^[^ ^6^, ^7^, ^8^ ^]^ It is also critical for the development of biomedical devices and tissue engineering scaffolds,^[^ ^9^, ^10^, ^11^ ^]^ the evaluation of structural integrity (e.g., bone health,^[^ ^12^ ^]^ and engineered or additively manufactured parts.^[^ ^13^, ^14^, ^15^ ^]^), and high‐fidelity computational mechanics modeling.^[^ ^16^, ^17^ ^]^ Typically, elastic properties vary spatially within these materials, necessitating advanced methods to characterize their heterogeneity.

Techniques for estimating elastic properties are generally classified into direct and indirect methods. Direct methods, such as nanoindentation and atomic force microscopy, assess elasticity distributions by inducing local deformation on the surface of the sample using a known force.^[^ ^18^, ^19^, ^20^, ^21^ ^]^ However, these direct methods are limited to small, localized areas of exposed surfaces, often requiring destructive procedures to access regions of interest, which limits their practicality for many applications. On the other hand, indirect methods determine elasticity distributions by measuring the displacement field resulting from an externally applied force on the surface. Common indirect methods include medical imaging techniques such as Magnetic Resonance Elastography (MRE),^[^ ^22^, ^23^ ^]^ and ultrasound‐based elastography methods.^[^ ^24^, ^25^ ^]^ Additionally, Digital Image Correlation (DIC) and Digital Volume Correlation (DVC) techniques are employed during mechanical testing to extract displacement fields from 2D and 3D imaging data, respectively.^[^ ^26^, ^27^, ^28^, ^29^, ^30^ ^]^ Materials with spatially varying elasticity often show complex patterns in displacement measurements and significant noise.^[^ ^31^, ^32^ ^]^ Conventionally, strain‐based elastography methods have been widely used in clinical applications. These methods assume a uniform stress distribution to determine elasticity distributions on a relative scale by inverting the strains. To achieve uniform stress, trained technicians apply the load uniformly over the surface. However, even with uniform loading, heterogeneous stress distributions may develop within the material, a phenomenon known as stress localization.

To achieve more robust and precise elasticity property estimations from the displacement fields, physics‐informed approaches incorporate governing physical principles. These principles are typically formulated as partial differential equations (PDEs) models, such as those describing linear elasticity.^[^ ^33^ ^]^ Estimating elasticity using PDE models is inherently ill‐posed, often resulting in unstable or non‐unique solutions.^[^ ^34^ ^]^ These approaches are categorized into direct and iterative methods. Direct methods simplify or reformulate linear elasticity PDE equations into forms solvable analytically or numerically, typically limited to simple geometries or idealized conditions.^[^ ^35^, ^36^, ^37^ ^]^ These methods generally require smooth displacements and strain fields without noise and auxiliary constraints such as average Young's modulus. Minor errors, such as noise in the data or inaccuracies in the constraints, can propagate through the estimation process, degrading the accuracy and stability of the solutions.^[^ ^35^ ^]^ Variational and weak‐form methods, such as the Virtual Fields Method (VFM),^[^ ^38^, ^39^ ^]^ and equilibrium‐gap formulations,^[^ ^40^, ^41^ ^]^ are derived from principles of energy minimization or virtual work. These approaches can estimate heterogeneous Young's modulus from full displacement fields,^[^ ^39^, ^42^, ^43^ ^]^ but they require the Poisson's ratio to be known.^[^ ^44^ ^]^ The iterative methods utilize finite element models (FEM) to minimize discrepancies between measured and simulated outputs iteratively.^[^ ^45^, ^46^, ^47^, ^48^, ^49^ ^]^ The iterative methods are computationally intensive and heavily dependent on the given initial conditions. A common challenge for all physics‐informed approaches is that displacement measurements are inevitably noisy, and numerical differentiation amplifies these errors, making accurate elasticity estimation difficult.^[^ ^50^, ^51^, ^52^ ^]^ Additionally, many methods typically assume incompressibility, which is impractical for many solid materials that exhibit compressibility and heterogeneous Poisson's ratios.^[^ ^53^, ^54^, ^55^ ^]^

Recent advancements in machine learning (ML) methods, such as Gaussian process regression (GPR),^[^ ^56^ ^]^ deep neural networks (e.g., generative adversarial networks,^[^ ^57^ ^]^ and convolutional neural networks.^[^ ^58^ ^]^) have enabled elasticity mapping using direct elasticity measurements. However, these purely data‐driven methods typically exhibit limited generalization and reduced accuracy when applied to materials with complex heterogeneity or geometries. An unsupervised learning employed with physics knowledge, as EUCLID‐based methods have shown impressive ability for homogeneous hyper elastic materials, but their formulations explicitly assume material homogeneity and are restricted to identifying global parameters rather than spatially heterogeneous distributions.^[^ ^59^, ^60^, ^61^, ^62^ ^]^

Physics‐informed neural networks (PINN), integrating physical laws directly into neural network architectures, have demonstrated success primarily in forward elasticity problems, predicting displacement or strain with given known parameters.^[^ ^63^, ^64^, ^65^ ^]^ In forward problems, the incorporated physical laws serve as a regularizer in PINNs, thereby enhancing robustness to noise. In contrast, inverse elasticity estimation with PINNs is significantly challenging because these models are highly sensitive to observational noise and fitting errors, which are magnified by the differentiations required to enforce physical consistency. Consequently, parameter estimation must rely on derivatives that are already amplified by error. Most existing inverse PINN approaches assume homogeneous elasticity with a constant value to be estimated.^[^ ^54^, ^55^, ^66^, ^67^, ^68^, ^69^, ^70^ ^]^ Heterogeneous elasticity estimation is generally challenging as, at every point, different elasticity values need to be estimated. Recent works addressing heterogeneous elasticity estimation with PINNs frequently struggle with noisy displacement data and often produce relative rather than absolute Young's modulus values.^[^ ^53^, ^71^, ^72^, ^73^, ^74^, ^75^ ^]^ To simplify the problem, strict assumptions are often made, such as known true (internal or boundary) stress distributions,^[^ ^73^, ^74^ ^]^ known mean Young's modulus,^[^ ^53^, ^75^ ^]^ and incompressible materials.^[^ ^53^, ^72^ ^]^

In this study, we propose an Inverse Elasticity PINN (IE‐PINN) model specifically designed to simultaneously estimate the spatial distributions of elasticity parameters, namely, Young's modulus and Poisson's ratio, from noisy displacement data based on the governing physics of linear elasticity, without requiring boundary force distribution information. Our IE‐PINN model demonstrates robust performance against noise, enabling accurate recovery of absolute Young's modulus distribution rather than merely relative distribution. A novel two‐step approach incorporating loading force boundary conditions facilitates precise absolute elasticity estimation. By combining with a specialized neural network architecture, our method achieves robust heterogeneous elasticity estimation with low errors based on noisy displacement datasets.

## 2 Result and Discussion

### 2.1 Inverse Elasticity Physic‐Informed Neural Network (IE‐PINN)

Estimating Young's modulus and Poisson's ratio from deformation fields constitutes an inherently ill‐posed inverse elasticity problem, often yielding non‐unique, unstable, and noise‐sensitive solutions. Conventional PINNs mainly target forward problems, benefiting from the automatic differentiation to compute PDE residuals analytically.^[^ ^76^ ^]^ However, high expressiveness of neural networks often leads to unstable or overly sensitive derivative estimates when using automatic differentiation. Automatic differentiation in IE‐PINN can lead to divergence in PDE residuals and poor elasticity estimation (Figure S1, Supporting Information). Recently, ElastNet has successfully estimated heterogeneous elasticity based on ideal noise‐free displacements;^[^ ^75^ ^]^ however, ElastNet directly applies finite difference to displacement measurement data without functional approximation, making it vulnerable and unstable in the presence of even small amounts of noise (Section 2.3). Additionally, ElastNet estimates only relative Young's modulus distributions,^[^ ^53^, ^75^ ^]^ requiring the true mean Young's modulus to derive absolute values. However, the true mean Young's modulus is typically not available in practice.

**Figure 1:**
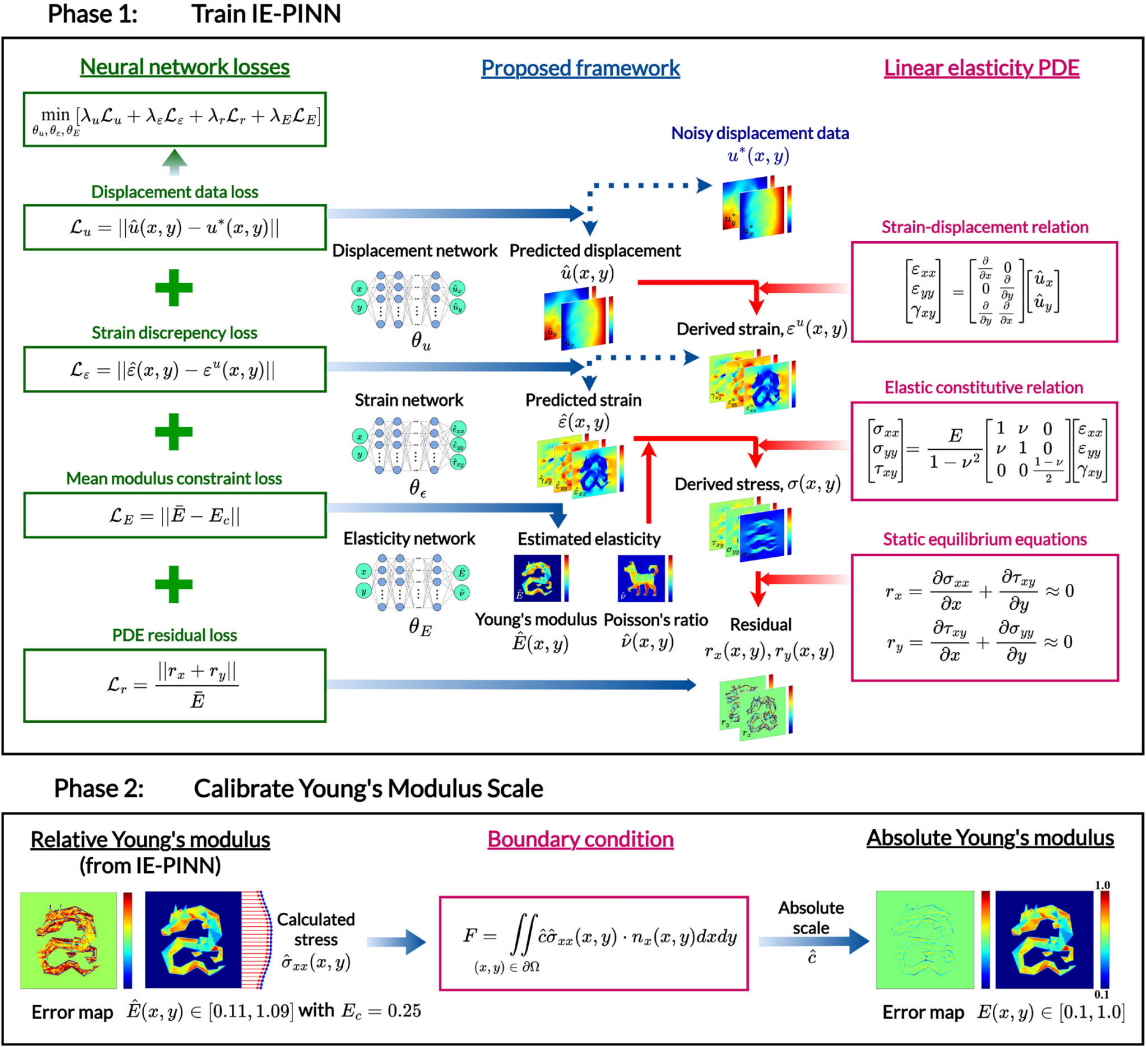
Framework for heterogeneous elasticity estimation from noisy displacement data. The framework consists of two distinct phases: the Inverse Elasticity Physics‐informed Neural Network (IE‐PINN) training phase and the Young's modulus scale calibration phase. In the first phase, three neural networks are trained using spatial coordinates to predict mechanical quantities, including displacement, strain, and elasticity (Young's modulus and Poisson's ratio), respectively. The displacement network is specifically employed to mitigate the adverse impact of noisy displacement measurements. The predicted displacements are used to compute the strain vector via the strain‐displacement relation. The strain network fits the strain derived by the displacement network. The elasticity network predicts Young's modulus and Poisson's ratio from spatial coordinates. Based on the constitutive equation, the stress tensor is derived from the strain tensor, Young's modulus, and Poisson's ratio. The static equilibrium loss is evaluated by the finite difference of the stress field. The goal of the training process is to estimate the parameters ($\theta_{u},\theta_{\varepsilon },\theta_{E}$) of all three neural networks by minimizing the (total) neural network loss, which evaluates the displacement network fitting, discrepancies in strain, deviations in mean modulus constraints, and the partial differential equation (PDE) residuals related to equilibrium equations. In Phase 2, the relative stress predicted at the boundary from Phase 1, combined with the experimental loading boundary conditions, is used to recover the correct absolute scale $\hat{c}$, resulting in an absolute‐scale distribution of Young's modulus.

To address the critical limitations of instability under noise and the inability to estimate Young's modulus on an absolute scale, we propose the IE‐PINN framework for robustly estimating heterogeneous Young's modulus and Poisson's ratio distributions from noisy displacement fields. **Figure** **1** illustrates the proposed methods, consisting of two phases. In Phase 1, IE‐PINN is trained based on noisy displacement data, and the spatial distributions of relative Young's modulus and Poisson's ratio are estimated. Then, in Phase 2, the absolute scale of Young's modulus is estimated (referred to as calibration), recovering the absolute scales of Young's modulus distribution by leveraging the estimated relative stress distributions from IE‐PINN.

To clearly demonstrate the advantages of IE‐PINN, throughout Section 2, we employ a dataset from ElastNet study,^[^ ^75^ ^]^ where the true spatial distribution of Young's modulus adopts a dragon shape and Poisson's ratio a dog shape, with displacements simulated using FEM. In this work, we use noisy displacement data generated by adding zero‐mean Gaussian noise to the displacements. The standard deviation of the noise is initially set to 0.1% of the average displacements (i.e., signal‐to‐noise, or SNR, ratio of 1000 as described in Section 4). In addition, comparative analyses at varying noise levels and patterns are conducted to evaluate the robustness of the proposed method (Section 2.3).

**Figure 2:**
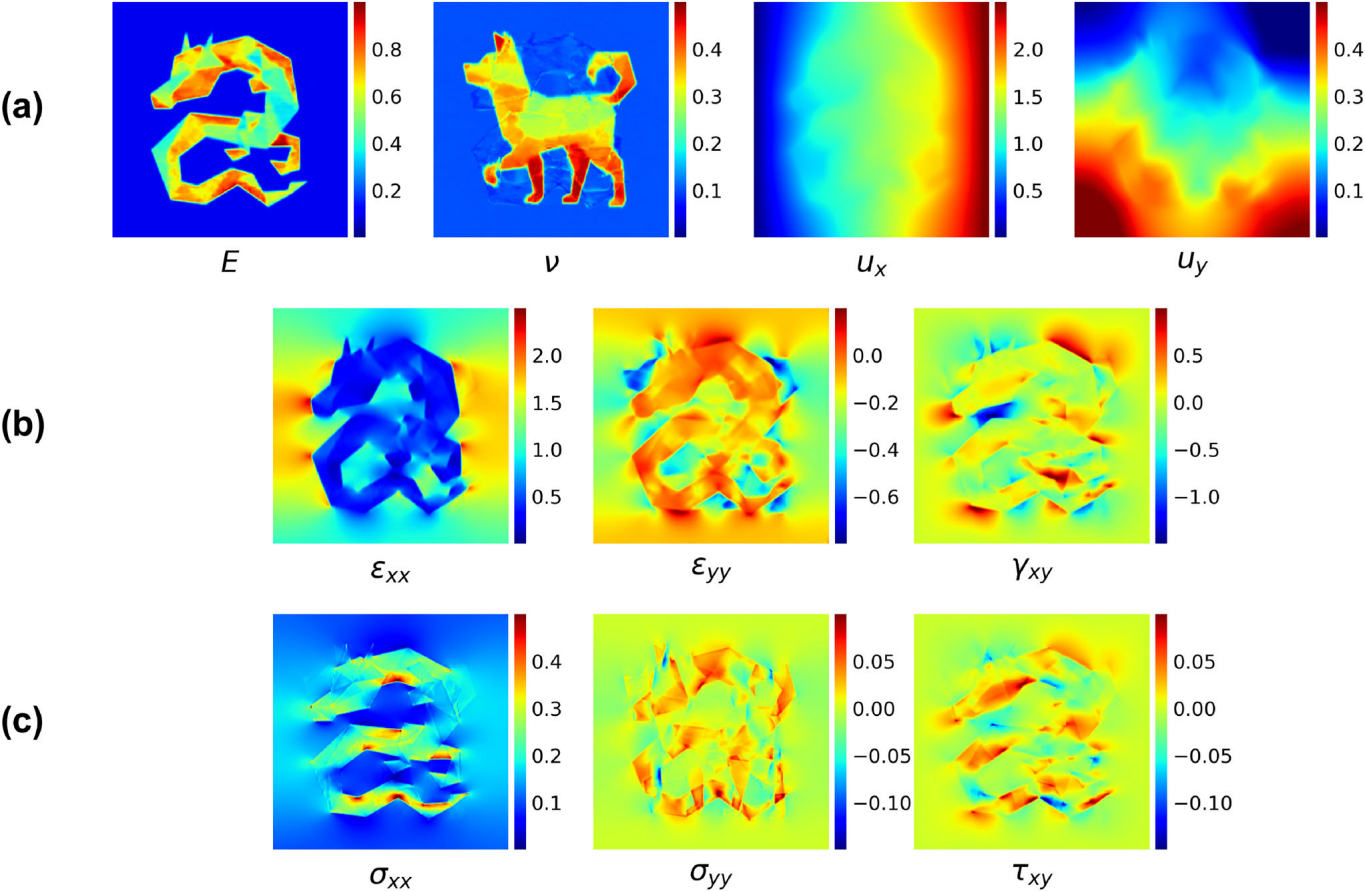
The prediction field of mechanical quantities. The model is applied to a measured displacement that contains a signal‐to‐noise ratio of 1000. a) The predicted Young's modulus field (MPa), Poisson's ratio field, and axial displacement field (mm). b) The predicted strain field (%). c) The predicted stress field (MPa).

The IE‐PINN architecture integrates the governing PDEs of linear elasticity, including the strain‐displacement relation, the elastic constitutive relation, and the equilibrium equations. The core innovation for achieving robust elasticity estimation in the presence of noisy displacements lies in the neural network framework, which comprises three deep neural networks: the displacement network, the extra strain network, and the elasticity network. The displacement network fits noisy displacement data ($u_{x}$ and $u_{y}$) with respect to spatial coordinates (x and y). Neural networks can effectively mitigate the adverse effects of noise through smoothing.^[^ ^77^ ^]^ In PINN, such smoothing is regulated by the PDE equations. Fitting neural networks to measurement data (i.e., displacements) is a widely used approach in PINNs.^[^ ^76^ ^]^ However, displacement fitting alone introduces high sensitivity in its second derivatives; fitting errors are amplified and propagated to the second derivative function, making the inverse elasticity estimation unstable, especially when the data are noisy. To address this, IE‐PINN incorporates a dedicated strain network, which decouples and predicts strain, thereby significantly reducing the sensitivity in second derivatives. The discrepancy between strains predicted by the strain network and those computed from predicted displacements is minimized through a strain discrepancy loss term. This approach substantially improves the stability and accuracy of elasticity estimation, even under noisy conditions. **Figure** **2** depicts the predictions made by the IE‐PINN for all relevant quantities, including denoised displacements, strains, stresses, and elasticity parameters. These predictions were trained using noisy displacement data, and their corresponding error maps are provided in Figure S2 (Supporting Information).

**Figure 3:**
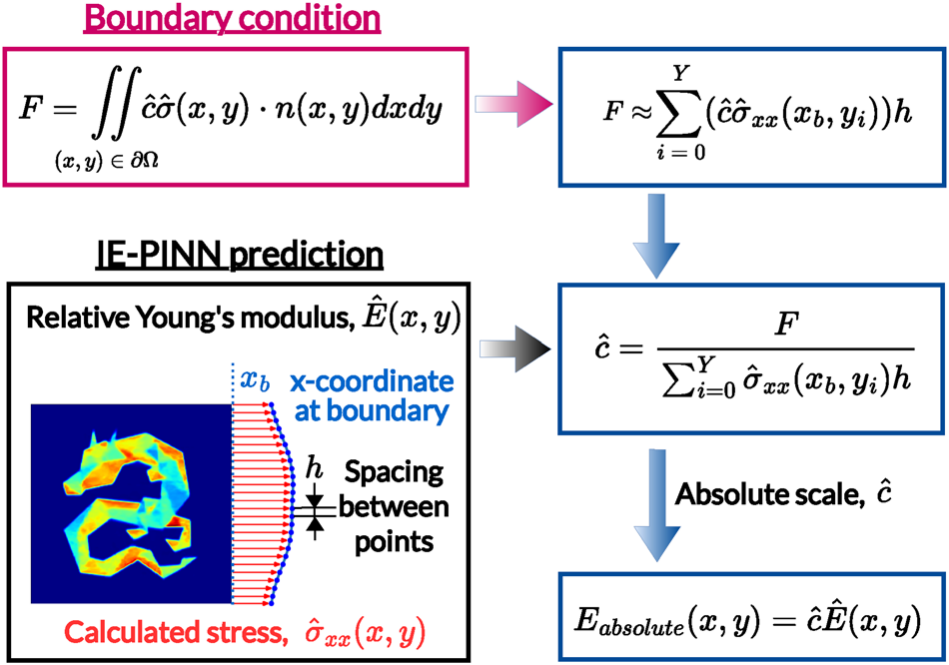
Young's modulus scale calibration procedure. Upon completion of Phase 1 training, the IE‐PINN provides a spatially varying relative Young's modulus field. In Phase 2, the absolute scale is calibrated by incorporating the known loading boundary conditions. Specifically, the predicted boundary stress from the relative Young's modulus is used to compute the resultant force, which is then aligned with the experimentally applied loading force to recover the true scale of Young's modulus.

Another significant innovation of IE‐PINN is its method for estimating absolute elasticity scales. Accurate absolute elasticity estimation requires precise enforcement of boundary conditions related to the applied loading force. Directly incorporating these boundary conditions into PINN loss functions often introduces gradient imbalances,^[^ ^78^ ^]^ leading to ill‐conditioned optimization landscapes.^[^ ^79^, ^80^ ^]^ To address this challenge, we calibrate the absolute scale of Young's modulus by aligning the boundary force, which is computed from the predicted stress based on the relative Young's modulus estimated from Phase 1, with the experimentally measured loading force, which is often available in practice.^[^ ^81^, ^82^, ^83^ ^]^ **Figure** **3** illustrates the calibration procedure employed in this study using numerical integration of the predicted relative stress under the applied loading force, where the technical details are described in Section 4. The proposed two‐step approach enables effective estimation of the elasticity distributions at the correct absolute scale, while maintaining training stability.

**Figure 4:**
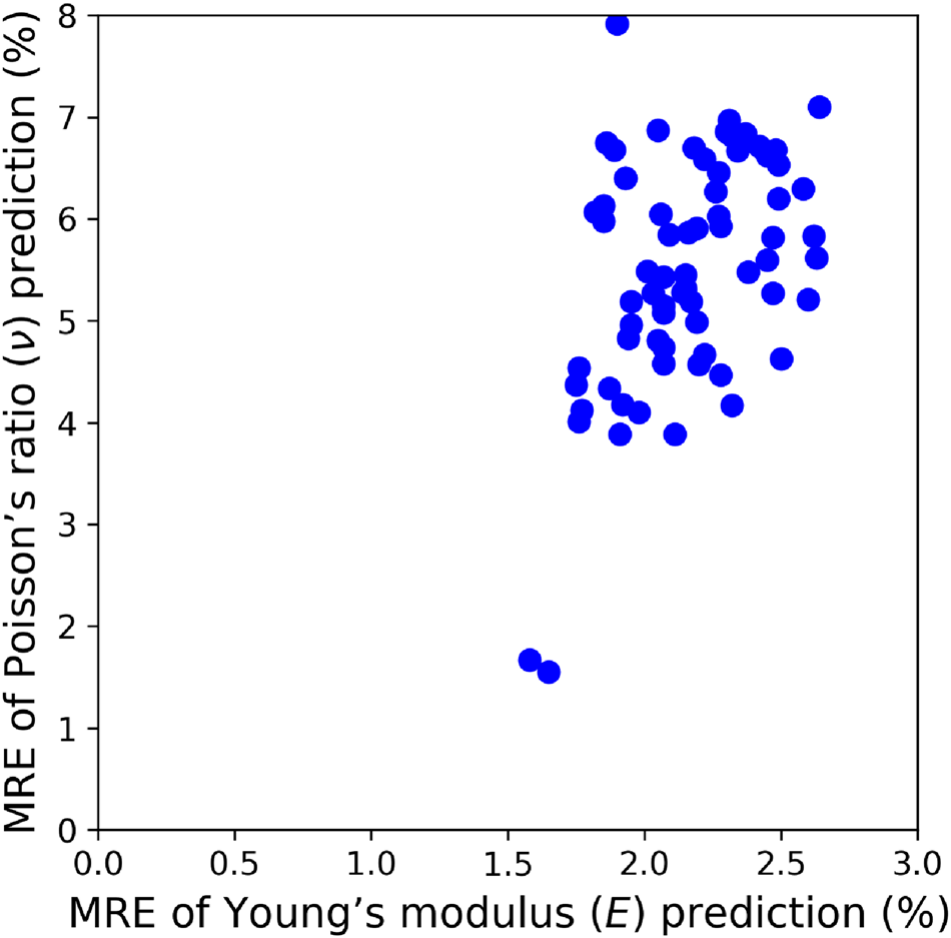
The accuracy of elasticity estimation across various types of elastic distribution patterns. The proposed model achieves a significantly low and consistently reliable mean relative error (MRE) across 50 independent datasets with noisy displacement data at a signal‐to‐noise ratio (SNR) of 1000, demonstrating robust accuracy in estimating the elasticity parameters.

**Figure 5:**
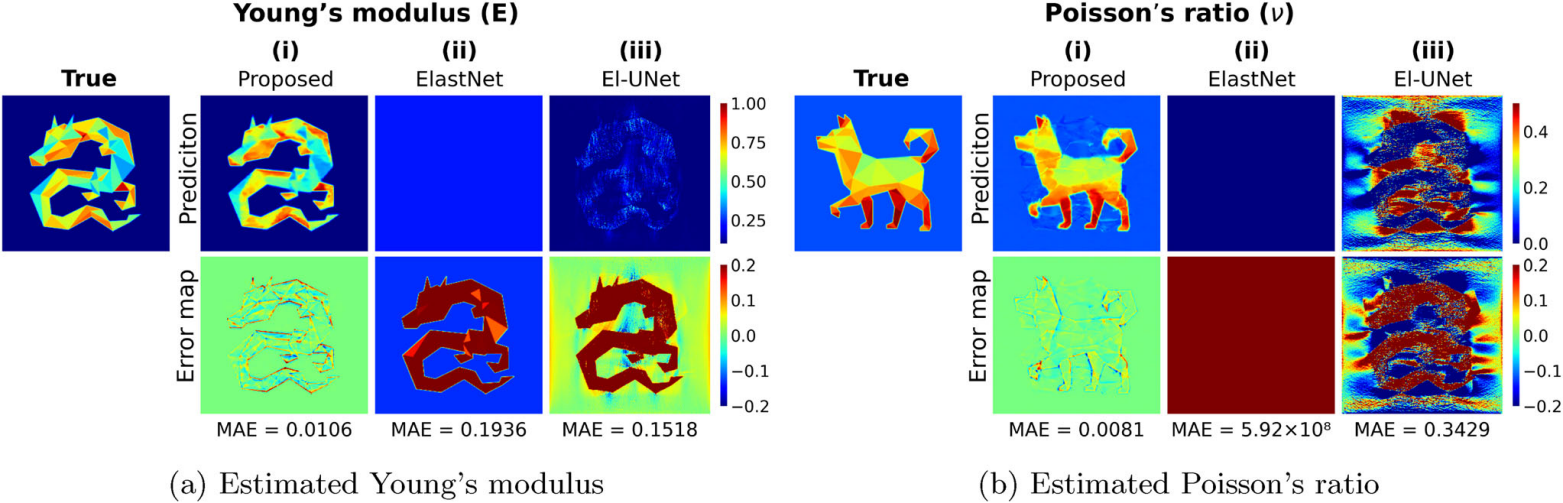
Estimations of Young's modulus (E) and Poisson's ratio (ν), and corresponding error maps, obtained from various state‐of‐the‐art PINN‐based methods. All models were trained on the same noisy displacement data with a signal‐to‐noise ratio (SNR) of 1000. i) IE‐PINN (Proposed) incorporates both displacement and strain networks. ii) ElastNet estimates elasticity directly from displacement observations via the finite difference.^[^ ^75^ ^]^ iii) EI‐UNet employs a Convolutional Neural Network (CNN) architecture with auto‐differentiation.^[^ ^74^ ^]^ However, all benchmark methods fail to recover the distributions of Young's modulus and Poisson's ratio in the presence of noise in the displacement data.

This research applies the proposed IE‐PINN to a thin plate scenario under plane stress conditions, where it predicts the stress distributions ($\sigma_{xx}$, $\sigma_{yy}$, and $\tau_{xy}$) based on constitutive and strain‐displacement equations, rather than assuming uniform distributions. The model minimizes a combined total loss function comprising displacement data, strain discrepancy, PDE residual, and mean modulus constraint losses during training. Each loss term is designed to reduce a specific source of errors: The displacement loss penalizes deviations between predicted and observed displacements; The strain discrepancy loss reduces the differences between the strain predictions from the strain network and those derived by differentiating the predicted displacements; The PDE residual loss enforces the governing equations by minimizing the PDE residuals computed from the strain and elasticity networks. Finite difference is employed for numerical differentiation. After training convergence, the IE‐PINN reliably predicts displacement, strain, stress, Young's modulus, and Poisson's ratio distributions. The proposed IE‐PINN performs robustly across multiple datasets with different spatial elasticity distributions (**Figure** **4**). In comparison to IE‐PINN's excellent estimation accuracy, two recent models for estimating heterogeneous elastic properties, ElastNet^[^ ^75^ ^]^ and EI‐UNet^[^ ^74^ ^]^, demonstrate estimation failure under the same noisy condition (**Figure** **5**). Moreover, IE‐PINN demonstrates stable convergence toward the ground‐truth elastic properties and exhibits low variability in the estimated parameters (Figure S3, Supporting Information).

In the following sections, we systematically demonstrate the advantages of each component of IE‐PINN, including the neural network architecture (Sections 2.2 and 2.6), robustness to varying noise levels (Section 2.3), sensitivity to the mean modulus constraint (Section 2.4), robustness to different boundary conditions (Section 2.5), and the impact of pretraining (Section 2.7).

### 2.2 Advantages of Displacement Fitting and Decoupled Strain Prediction

**Figure 6:**
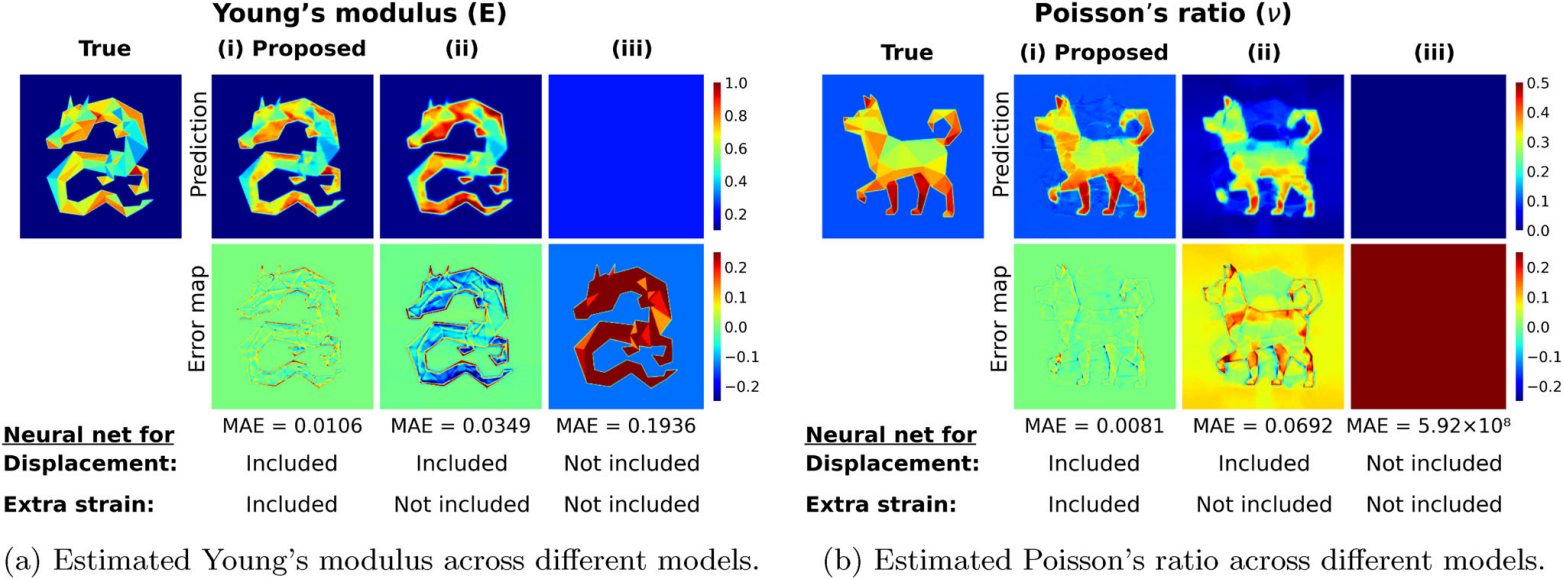
Young's modulus (E) and Poisson's ratio (ν) estimations and corresponding error maps were obtained from different models. All models were trained using the same noisy displacement data with a signal‐to‐noise (SNR) ratio of 1000. i) IE‐PINN (Proposed) incorporates both displacement and strain networks. ii) Model with only a displacement network. iii) Model without both displacement and strain networks, similar to ElastNet.^[^ ^75^ ^]^ Employing Function approximation over noisy displacement data tends to denoise the measurements and improve the stability. Additionally, incorporating a dedicated strain network enhances the robustness and accuracy of elasticity estimation.

For robust elasticity estimation from noisy displacement data, using a neural network fitted to displacement measurements is essential. **Figures** **6**(a) and 6(b) compare the estimation and error maps of Young's modulus and Poisson's ratio produced by the proposed IE‐PINN model against those obtained from variants in which the strain (and displacement) networks are excluded. Figure 6a(iii), b(iii) corresponds to the variant in which both displacement and strain networks are excluded. In this case, finite difference is applied directly to the noisy displacement fields to compute strain, which is then used to estimate stress. This configuration is consistent with the approach used in ElastNet.^[^ ^75^ ^]^ Direct differentiation significantly amplifies the errors in the noise, making the elasticity estimation vulnerable to noise. These results clearly demonstrate the detrimental impact of noise on this approach, which fails to estimate both Young's modulus and Poisson's ratio. Fitting displacement data alone can mitigate the adverse effects of noise to some extent. Figure 6a(ii),b(ii) demonstrates that both Young's modulus and Poisson's ratio are estimated with some errors. Nonetheless, the displacement network brings another challenge: it is sensitive to its second derivative. Data fitting errors propagated to the second derivative make the elasticity estimation unstable. As a result, in other datasets, solely using the displacement network also often fails in elasticity estimation (Figure S4a(ii), b(ii), Supporting Information), whereas the full IE‐PINN model consistently maintains accurate estimates. Figure 6a(i),b(i) presents our proposed method, which reduces the estimation errors significantly in both Young's modulus and Poisson's ratio. The estimated values closely match the ground‐truth values. The strain network not only improves the accuracy of elasticity estimation but also stabilizes the elasticity estimation significantly under the noisy conditions.

Under high noise conditions, the displacement network alone is not sufficient. In contrast, the proposed IE‐PINN successfully estimates both Young's modulus and Poisson's ratio, confirming its robustness against noise (Figures S5 and S6, Supporting Information).

### 2.3 Robustness to Displacement Noise

**Figure 7:**
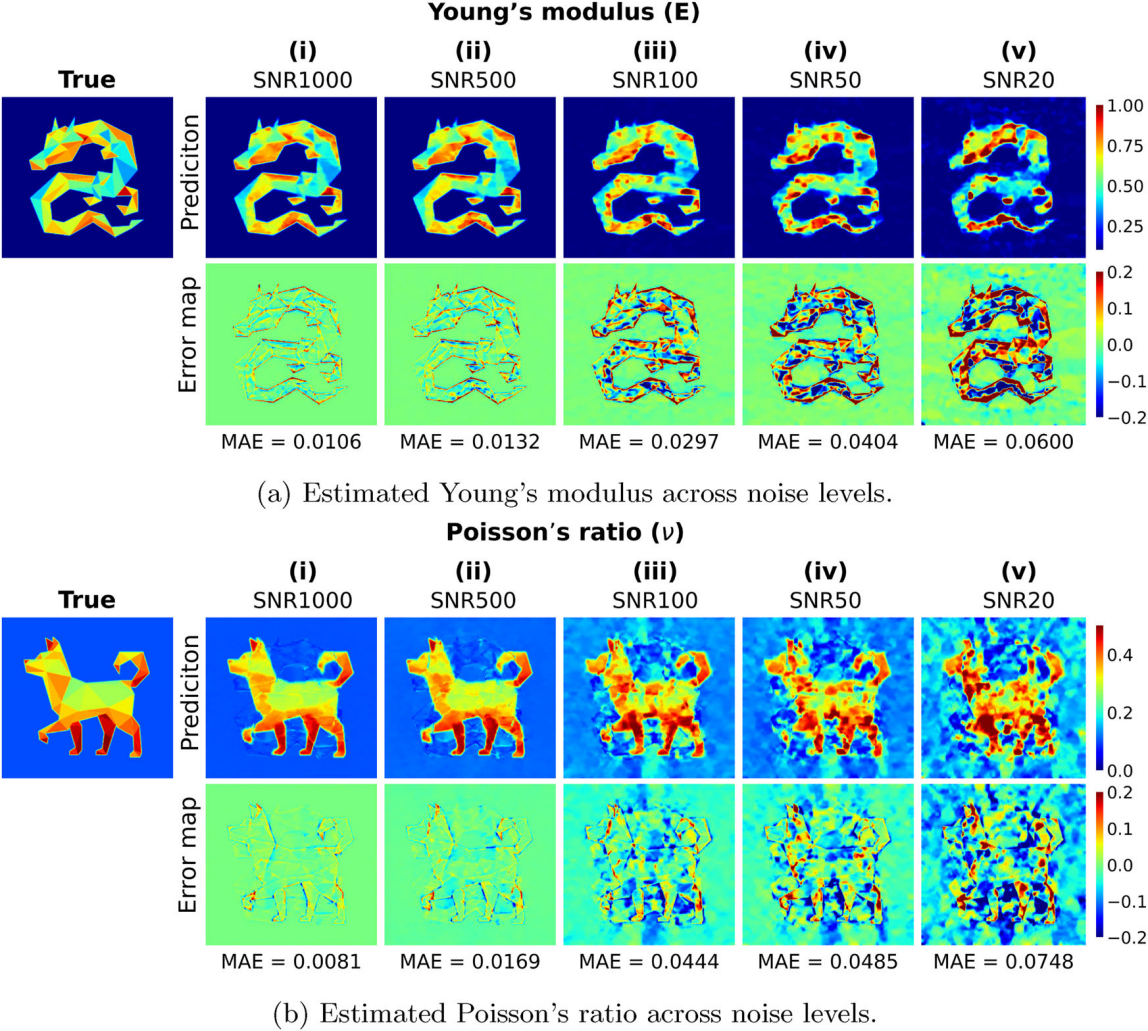
Robustness of IE‐PINN under noisy displacement data. estimated field of a) Young's modulus (E) and b) Poisson's ratio (ν), along with their corresponding error maps, are evaluated across varying noise levels, defined by signal‐to‐noise ratio (SNR): i) SNR = 1000, ii) SNR = 500, iii) SNR = 100, iv) SNR = 50, and v) SNR = 20. IE‐PINN was trained using the displacement data across five different SNRs. Although estimation errors increase with higher noise, the model maintains robust performance across all noise levels.

**Figure 8:**
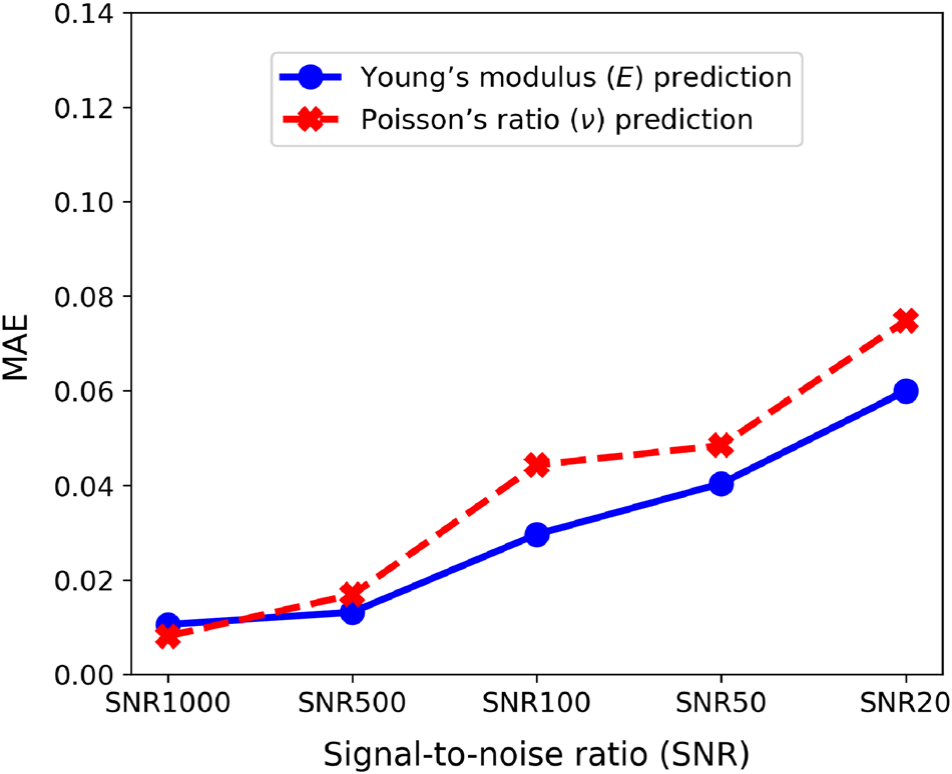
Robustness of estimation errors across different noise levels. The predicted Young's modulus and Poisson's ratio exhibit strong robustness to noise, with estimation errors increasing linearly as noise levels increase.

Noise in observed displacements presents significant challenges for inverse problems, as it is strongly amplified during numerical differentiation (Figure S7, Supporting Information), thereby worsening the accuracy of elasticity estimation. In contrast, the IE‐PINN robustly recovers spatial derivatives under noisy conditions by leveraging its specialized architecture with the three coupled networks (Figure S8, Supporting Information). In this work, we primarily use independent Gaussian noise, which is widely adopted in the literature.^[^ ^59^, ^84^, ^85^, ^86^ ^]^ However, because spatially dependent structured noise is often encountered in real data,^[^ ^87^, ^88^ ^]^ IE‐PINN is also validated under structured noise. To study the sensitivity of the IE‐PINN to displacement noise, we investigate five signal‐to‐noise ratios (SNRs), defined as the ratio of the mean displacement to the standard deviation of the noise: 1000, 500, 100, 50, and 20. Under Gaussian noise, the predicted fields and error maps for Young's modulus and Poisson's ratio are presented in **Figure** **7**a,b, respectively. The mean absolute errors (MAE) of IE‐PINN in the estimation of Young's modulus and Poisson's ratio across these noise levels are shown in Figure 8. The results demonstrate that estimation errors remain low across noise levels, confirming the model's robustness in estimating elastic properties. As the noise level increases tenfold (from SNR 1000 to 100), the errors in both Young's modulus and Poisson's ratio increase moderately but remain within acceptable bounds. In the estimated Young's modulus fields (Figure 7a), results at SNR 1000 and 500 closely match the ground truth. At SNR 100 and 50, the overall dragon‐shaped distribution is largely preserved, though boundary sharpness and internal detail degrade. Even at SNR 20, where noise has a standard deviation of 5% of the mean displacement, the global structure remains identifiable despite diminished fine‐scale features. Poisson's ratio estimations (Figure 7b) show similar trends. At SNR 1000 and 500, the estimates align well with the true field, with only minor errors in the background. At SNR 100, deviation becomes noticeable, but key structures remain identifiable. Under severe noise (SNR 20), the estimates degrade substantially; however, some sub‐regions retain partial accuracy. The proposed IE‐PINN generally shows robustness across fifty datasets, randomly selected from ElastNet,^[^ ^75^ ^]^ as shown in Figure 4. The estimated results for five randomly selected datasets at SNR 1000 are presented in Figures S9–S13 (Supporting Information) and at SNR 100 are presented in Figures  S14–S18 (Supporting Information).

**Figure 9:**
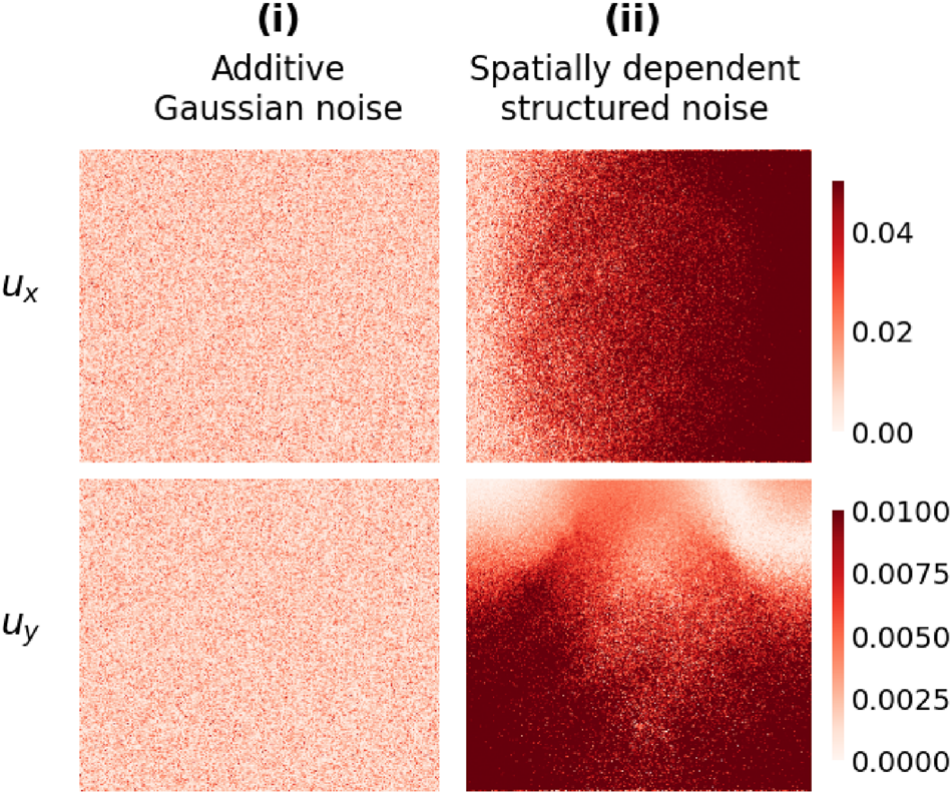
Comparison of two noise patterns: i) additive Gaussian noise and ii) spatially dependent structured noise (SNR 100).

**Figure 10:**
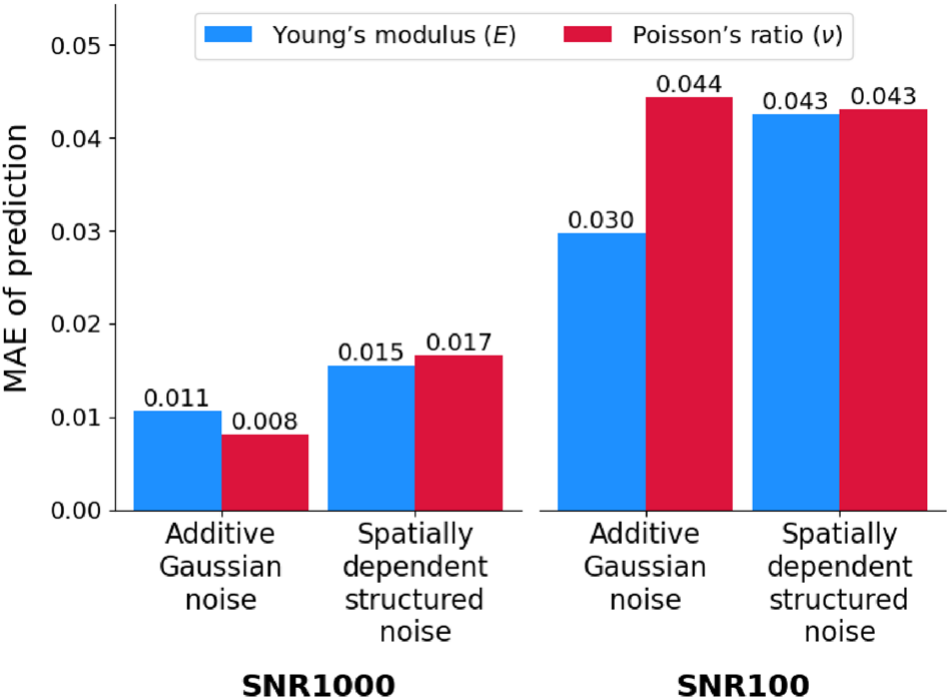
Robustness of IE‐PINN under different noise patterns. Estimation errors for Young's modulus and Poisson's ratio are slightly lower under additive Gaussian noise than under spatially dependent structured noise.

Structured noise, such as ultrasound artifacts, is common in clinical imaging and exhibits correlated spatial patterns. To validate performance under realistic conditions, we generate structured noise as illustrated in **Figure** **9**ii, with technical details provided in Section 4.^[^ ^89^, ^90^ ^]^ **Figure** **10** presents the MAE of the estimated Young's modulus and Poisson's ratio under Gaussian and structured noise at SNR levels of 1000 and 100. Because structured noise distorts the overall displacement patterns, estimation becomes more difficult, and the resulting errors are generally higher than under Gaussian noise. Nevertheless, IE‐PINN maintains strong robustness across SNR levels, as demonstrated in Figure S19a,b (Supporting Information), which present the predicted fields and error maps. Remarkably, even at SNR 20, the dragon and dog shapes in the underlying elasticity maps are clearly recovered.

### 2.4 Absolute Scale Calibration and Mean Young's Modulus Constraint

The boundary condition on the loading force is critical as it determines the unit of Young's modulus. Without this boundary condition, only the relative distribution can be obtained. However, integrating the boundary conditions directly into the loss function through boundary condition residuals ($r_{bc}$), weighted by the corresponding loss weight ($\lambda_{bc}$), can cause training instability or even failure. The training performance is often highly sensitive to the choice of this weight. The performance of conventional PINNs under single‐phase training, where boundary conditions are enforced with fixed loss weights, shows strong sensitivity to weight $\lambda_{bc}$ (Figure S22, Supporting Information). In contrast, IE‐PINN remains robust under varying boundary weight configurations (Figure S23, Supporting Information). Previous methodologies typically assume prior knowledge of the true mean of the Young's modulus distribution,^[^ ^53^, ^75^ ^]^ or stress distributions,^[^ ^73^, ^74^ ^]^ which is generally unavailable in practical applications, complicating the estimation of elasticity on an absolute scale. To overcome this limitation, IE‐PINN initially estimates Young's modulus with an arbitrary mean value, generating a relative modulus distribution. Subsequently, IE‐PINN employs a novel calibration method to recover the absolute scale by aligning the predicted relative boundary stress distribution with experimentally measured loading forces (Figure 3).

**Figure 11:**
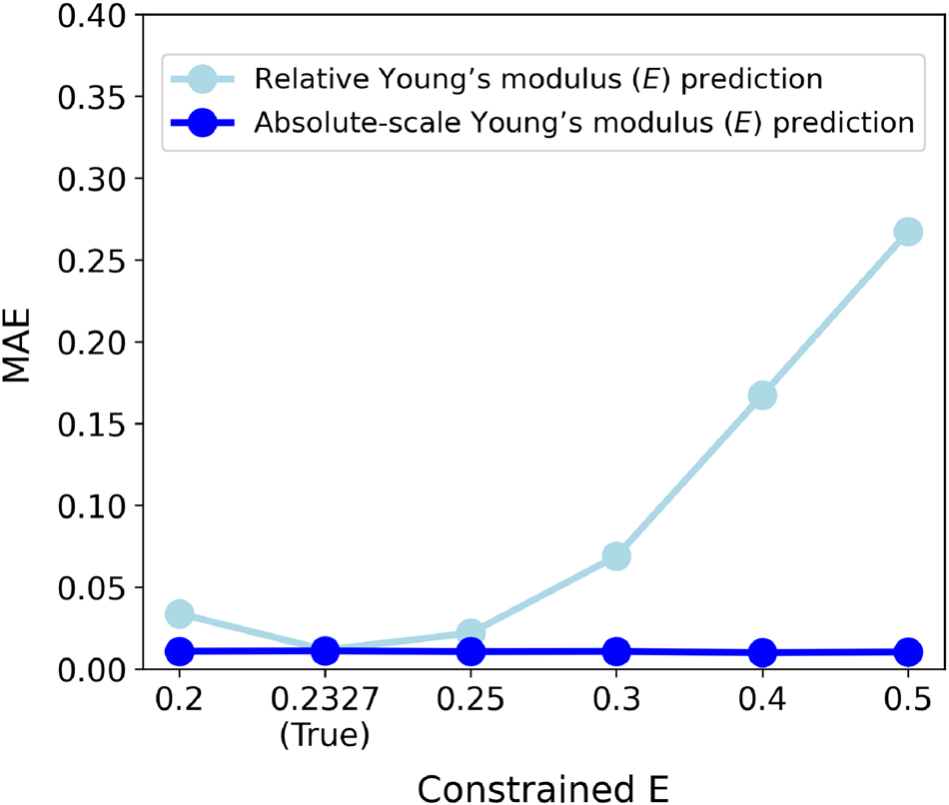
Impact of constrained mean Young's modulus. Although the model is trained with various values of constrained mean Young's Modulus, the proposed absolute scale calibration technique effectively adjusts the estimated relative Young's modulus from IE‐PINN in Phase 1 to their corresponding absolute scales. The estimation errors remain consistent across different constrained mean values, demonstrating the robustness of the calibration approach.

**Figure** **11** demonstrates the impacts of various arbitrary mean constraints on the estimation errors both before and after scale calibration; the estimation MAEs are provided in Table S1 (Supporting Information). Initially, high MAE is observed before calibration due to inaccurate mean elasticity assumptions. Nevertheless, the proposed calibration technique successfully identifies the correct scale from boundary stress predictions, achieving performance comparable to the ideal case. The figure confirms that the calibration performance is significantly robust against the constrained mean modulus values. Poisson's ratio estimates are also consistently robust across imposed mean constraints (Figure S24, Supporting Information). This independent calibration scheme maintains precise absolute‐scale Young's modulus estimation while preserving the training stability of IE‐PINN.

### 2.5 Robustness to Boundary Conditions

**Figure 12:**
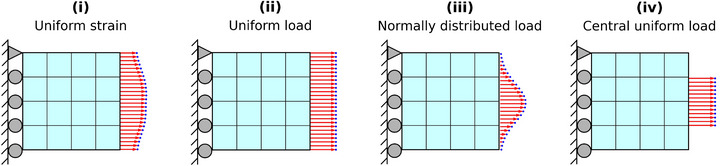
Boundary loading conditions. (i) Uniform strain: a load is applied to achieve uniform strain along the boundary. (ii) Uniform load: a load is applied uniformly along the boundary. (iii) Normally distributed load: a load following a normal distribution is applied along the boundary. (iv) Central uniform load: a uniform load, similar to (ii), is applied only to the central portion of the boundary.

Generally, boundary loading conditions significantly influence the deformation behavior of an object. To evaluate the robustness of the IE‐PINN under varying boundary conditions, we consider four distinct loading scenarios as illustrated in **Figure** **12**. In all scenarios, loading forces are applied to the right boundary of the object. In the first scenario, the loading force is applied to produce uniform strains along the boundary; this dataset is obtained from ElastNet.^[^ ^75^ ^]^ The second scenario applies a uniform force distribution along the entire boundary. The third scenario imposes the boundary experiences a normally distributed force along the boundary. In the final scenario, a central uniform load is applied only to the middle 50% portion of the boundary. For each of these boundary conditions, FEA simulations are performed to generate the displacement field based on three different distributions of elastic properties. Gaussian noise corresponding to a signal‐to‐noise ratio (SNR) of 1000 is added to the displacement data.

**Figure 13:**
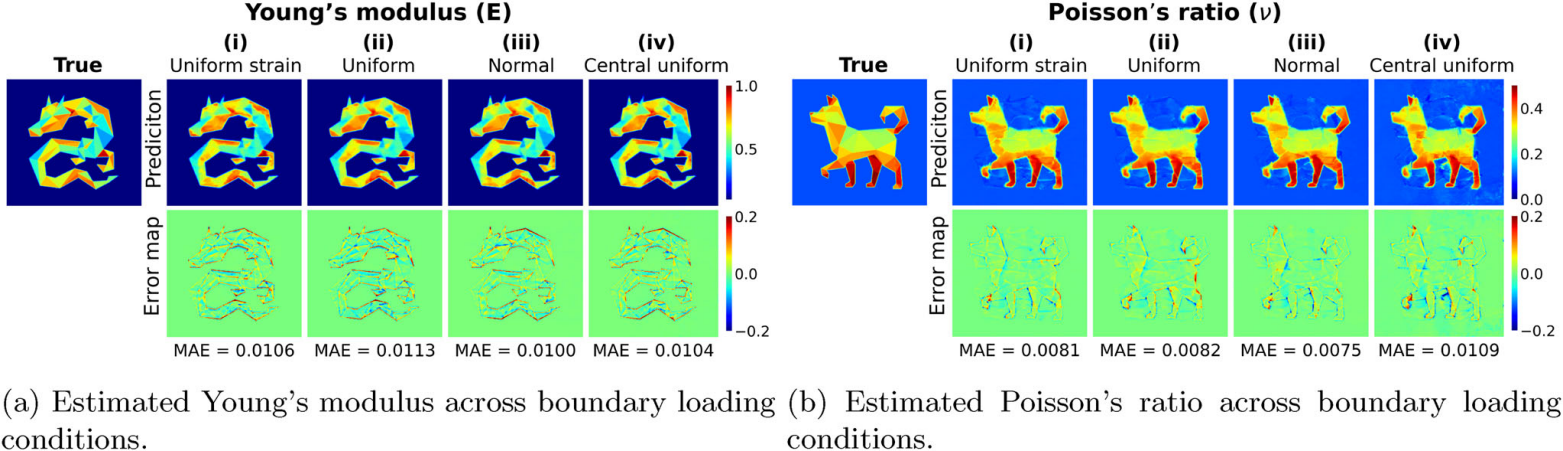
Predicted a) Young's modulus (E) and b) Poisson's ratio ((ν)), along with their corresponding error maps, evaluated under four different boundary loading conditions. The dataset is characterized by a distribution of Young's modulus in the shape of a dragon and Poisson's ratio in the shape of a dog. The model exhibits robust and accurate performance across all loading conditions.

**Figure** **13** demonstrates that the performance of the IE‐PINN remains consistently accurate across all four loading scenarios. The estimated elastic properties closely match the ground‐truth distributions under each boundary condition, indicating strong robustness of the proposed method. This consistency is further validated by results from two additional datasets shown in Figures S20 and S21 (Supporting Information).

Additionally, the imprecise loading condition is discussed in Note S1 (Supporting Information). Even though Phase 2 of IE‐PINN relies on the magnitude of the applied force, which is generally accurate. The sensitivity of the Young's modulus estimation to errors in force measurements is quantitatively analyzed in Table S2 (Supporting Information; details are included in Note S2, Supporting Information). The accuracy of Young's modulus may be marginally affected by force measurement errors, but the Poisson's ratio remains unchanged.

Moreover, even when the loading boundary condition is unknown (a common scenario in strain‐based elastography^[^ ^91^ ^]^), IE‐PINN can accurately estimate both the relative Young's modulus and Poisson's ratio fields under noisy displacement measurements, whereas ElastNet fails under noisy displacement data. This capability is essential for extending the applicability of IE‐PINN to broader domains.

### 2.6 Positional Encoding and Activation Functions

**Figure 14:**
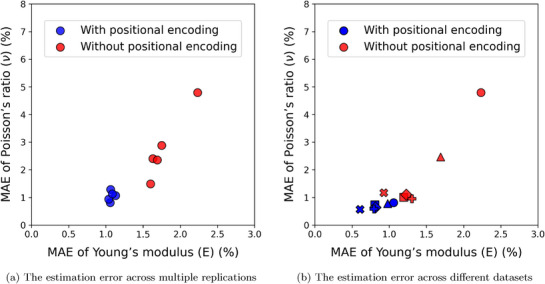
Benefits of the Positional Encoding Function. a) The estimation error with the positional encoding shows improved and consistent performance across multiple replications. b) The estimation error across various datasets that utilize positional encoding features further emphasizes the advantages of robust estimation and enhanced accuracy.

Neural networks with low‐dimensional positional inputs (coordinates x and y) often struggle to represent complex functions,^[^ ^92^, ^93^ ^]^ PINNs also often struggle to capture high‐frequency wavelets or sharp transitions in the target response due to spectral bias.^[^ ^94^ ^]^ These factors can lead to ill‐conditioned optimization problems and result in gradients that lack informativeness. To address this, we integrate a positional encoding technique,^[^ ^95^ ^]^ which transforms the two‐dimensional coordinates into a richer high‐dimensional latent representation. This approach enables the representation of highly detailed functions using low‐dimensional inputs,^[^ ^93^ ^]^ and improves the accuracy of elasticity estimations as shown in **Figure** **14**. On the same dataset, the use of positional encoding produced more consistent results across multiple replications, improving the accuracy of both Young's modulus and Poisson's ratio estimates (Figure 14). This improvement is not confined to a single dataset; when applied to multiple datasets, positional encoding continued to yield superior estimation performance (Figure 14).

**Figure 15:**
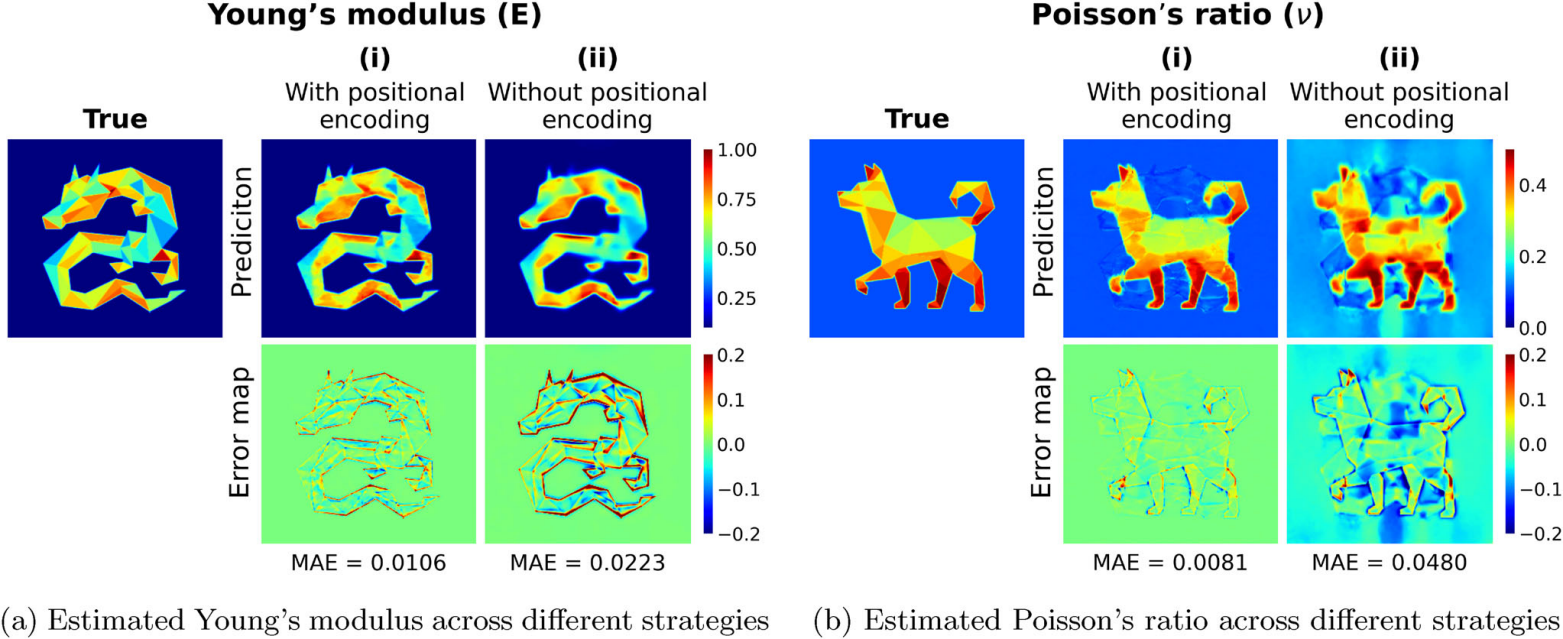
The estimation field of elasticity from two different types of coordinates. The coordinates with positional encoding function ((a)(i) and (b)(i)) can yield higher accuracy and resolve the spectral bias shown in estimation from the model without positional encoding function ((a)(ii) and (b)(ii)).

**Figure** **15** highlights the significance of the positional encoding function. The estimations made using the positional encoding coordinates, as shown in Figure 15a(i), result in superior estimates for both Young's modulus and Poisson's ratio. This approach effectively captures the details in the Young's modulus field and addresses the spectral bias observed in estimations made without the positional encoding function (see Figure 15a(ii)). Additionally, the model that incorporates positional encoding coordinates demonstrates improved estimations for Poisson's ratio as well.

**Figure 16:**
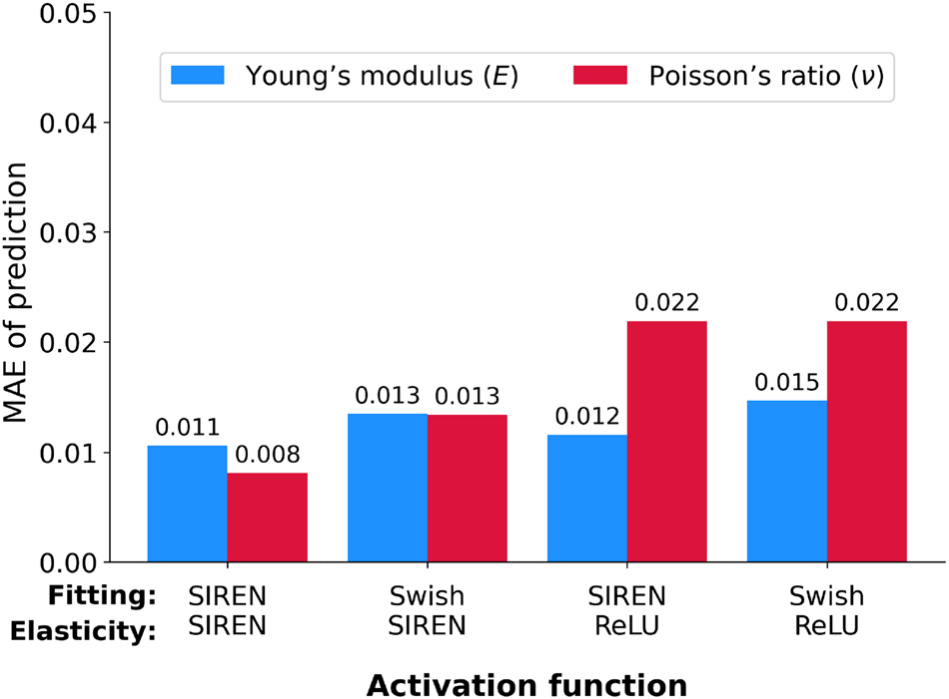
Performance comparison across different activation functions. The estimation errors in Young's modulus (E) and Poisson's ratio (ν) are presented for models using various activation functions in neural networks (**Fitting** denotes both displacement and strain networks, **Elasticity** denotes elasticity network). Among the activation functions evaluated, the SIREN activation function achieved the highest accuracy across all elastic property estimations.^[^ ^92^ ^]^

Another critical aspect influencing neural network performance in PINN is the choice of activation functions. Traditional activation functions often suffer from vanishing/exploding gradients during training, reducing training efficiency. IE‐PINN employs the sine activation function (SIREN), which has proven effective in precise gradient and divergence prediction.^[^ ^92^ ^]^ **Figure** **16** compares elasticity estimation errors across different activation functions, specifically evaluating the SIREN with Swish, used in ElastNet. ^[^ ^75^ ^]^ The same activation functions are used for displacement and strain fitting (denoted as Fitting) and those for Young's modulus and Poisson's ratio (denoted as Elasticity). The results demonstrate that using SIREN consistently produces the lowest MAEs for both Young's modulus and Poisson's ratio estimations.

### 2.7 Pretraining Strategy

Training PINNs is often more challenging than training conventional neural networks, primarily due to the complexity of the multi‐term loss function and the computational cost and instability associated with numerical differentiation used to compute PDE derivatives.^[^ ^96^, ^97^ ^]^ IE‐PINN involves three distinct neural networks, each with specific loss terms and their roles in solving the linear elasticity PDEs, including displacement fitting, strain discrepancy, and PDE residual losses. To enhance the training efficacy, IE‐PINN employs a sequential pretraining strategy. The pretraining stage consists of two sequential steps. First, the displacement network is fitted to the observational data for 50,000 iterations using a loss function that includes only the displacement data loss term ($L_{u}$). Second, the strain network is added and trained for 100,000 iterations using a strain discrepancy loss term ($L_{\varepsilon }$); the strain network is fitted to the strain field computed from the output of the displacement network.

Following this pretraining stage, the elastic network is incorporated into the architecture, and the loss function is augmented to include the PDE residual loss associated with the governing PDEs ($L_{r}$). This enables subsequent training of the full physics‐informed model.

The key idea behind this pretraining strategy is to fit only the minimum necessary components at each stage. In the first step, only the displacement network is optimized, and thus the loss weighting configuration remains simple. Moreover, during pretraining, the PDE residual terms are excluded, avoiding the need to compute derivatives. Despite this simplification, both the displacement and strain networks are aligned with the observational data prior to full model training. This approach not only improves estimation accuracy but also significantly reduces training time. Additional details are provided in Note S3 (Supporting Information).

**Figure 17:**
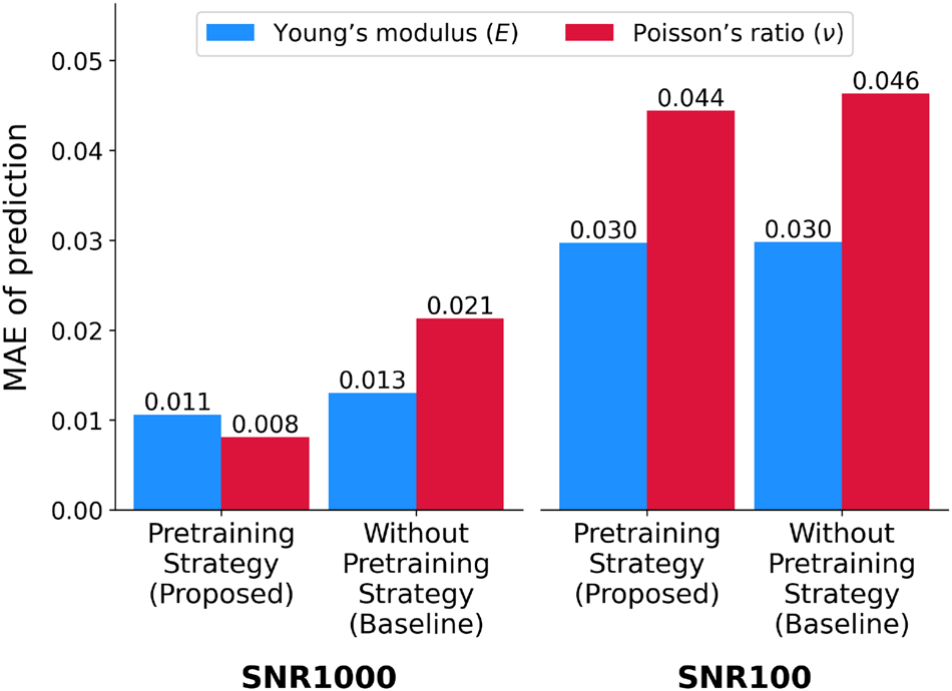
Impact of pretraining strategy. The estimation errors in Young's modulus (E) and Poisson's ratio (ν) are compared between two training strategies: A pretraining scheme that sequentially trains neural networks versus without pretraining training strategy. Models are evaluated using noisy displacement data under two levels, corresponding to SNR of 1000 and 100. The results demonstrate that employing a pretraining strategy significantly improves the prediction accuracy of both elasticity parameters, achieving up to 50% reduction in error.

**Figure** **17** compares the performance of IE‐PINN with and without a pretraining strategy under different noise levels (SNR of 1000 and 100) under the same total number of training iterations. The results demonstrate that pretraining effectively improves estimation accuracy, confirming its effectiveness in both low and high noise levels.

### 2.8 Related Work and Future Study

Several studies have explored inverse PINNs for elasticity estimation. Early approaches primarily assumed homogeneous materials with constant elasticity; some relied on both displacement and stress measurements,^[^ ^54^, ^66^, ^67^, ^68^ ^]^ while others used only displacement data.^[^ ^55^, ^69^, ^70^ ^]^ More recent efforts have extended to heterogeneous materials for spatially varying elasticity estimation. Some studies assume incompressible material behavior and estimate only Young's modulus while assuming Poisson's ratio is constant.^[^ ^53^, ^72^ ^]^ Due to the ineffective performance after integrating the boundary condition on the loading force, some works exclude the boundary conditions and obtain a relative Young's modulus distribution rather than an absolute scale.^[^ ^72^, ^75^ ^]^ The prior knowledge of the true mean of Young's modulus can be integrated as another loss to obtain the accurate distribution of elasticity distributions.^[^ ^53^, ^75^ ^]^ Other methods use strain data under the assumption of a known boundary stress distribution, which is typically unavailable in real‐world scenarios.^[^ ^73^, ^74^ ^]^

ElastNet uses the strain data for incompressible materials in the earlier model,^[^ ^53^ ^]^ and displacement data for compressible materials in the more recent version,^[^ ^75^ ^]^ employing finite‐difference approximations to estimate elasticity. Unlike typical PINN‐based methods, ElastNet does not fit a functional model (e.g., neural networks); instead, it directly applies numerical differentiation to all the variables, including noisy measurements, making it particularly sensitive to noise, as discussed in Section 2.2. Additionally, boundary conditions are not incorporated into their estimation, resulting in the estimated Young's modulus being expressed in relative scales rather than absolute ones. In contrast, our proposed IE‐PINN demonstrates robustness to measurement noises and enables estimation of heterogeneous Young's modulus on an absolute scale, based on the externally applied force that can be experimentally measured using localized mechanical testing techniques such as ultrasound,^[^ ^81^ ^]^ nanoindentation,^[^ ^82^ ^]^ and atomic force microscopy.^[^ ^83^ ^]^

The IE‐PINN model shows promise for inferring the underlying elastic properties from deformation data, in biomechanics,^[^ ^98^ ^]^ clinical ultrasound imaging,^[^ ^99^ ^]^ and magnetic resonance elastography (MRE).^[^ ^100^, ^101^ ^]^ However, several important challenges remain for future work.

First, in this work, IE‐PINN is extensively validated on FEM‐simulated data. Synthetic validation is critical because it provides exact ground truth and enables systematic variation of elasticity maps, boundary conditions, and noise structures, thereby allowing rigorous assessment of accuracy and robustness.^[^ ^59^, ^74^, ^75^, ^86^, ^88^, ^102^, ^103^, ^104^ ^]^ Nevertheless, validation on experimental data remains essential. As future work, we plan to extend IE‐PINN to experimental datasets, where noise patterns and material heterogeneity are more complex and less controlled, thus further demonstrating its practical applicability.

Second, uncertainty quantification represents a promising direction. Existing Bayesian inversion approaches either require extensive forward FEA for Monte Carlo sampling, leading to prohibitive computational cost, or restrict parameterizations to low‐dimensional constitutive spaces. Extending IE‐PINN with Bayesian formulations could enable robust uncertainty quantification.

Third, biomechanical applications often involve complex geometries.^[^ ^105^ ^]^ In such cases, applying accurate boundary conditions becomes particularly challenging, especially when surface normals are difficult to estimate. Additionally, extending the estimation of three‐dimensional elasticity remains an open research direction.

Fourth, the nonlinear behavior of soft tissues in clinical imaging,^[^ ^106^ ^]^ limits the direct applicability of IE‐PINN, which is based on linear elasticity. To address this, one may incorporate hyperelastic models, such as the Saint Venant‐Kirchhoff formulation,^[^ ^107^ ^]^ to better capture physiological responses.

Finally, practical limitations remain. IE‐PINN is computationally intensive, which currently hinders real‐time use, and its accuracy is constrained by the low spatial resolution of many experimental measurements. Moreover, like other PINN‐based methods, IE‐PINN requires retraining for each new geometry or boundary condition. Exploring transfer learning or meta‐learning strategies to minimize the need for retraining is an exciting direction for future research.

Addressing these challenges is essential to establishing IE‐PINN as a viable framework for biomechanical analysis and clinical translation.

## 3 Conclusion

In this work, we present an Inverse Elasticity Physics‐Informed Neural Network (IE‐PINN) model designed to effectively estimate heterogeneous Young's modulus and Poisson's ratio from noisy displacement data. This model addresses the challenges of measurement noise and the difficulty of accurately recovering Young's modulus at the correct absolute scale. IE‐PINN employs three distinct neural networks to stabilize the estimation, each predicting displacements, strains, and elasticity parameters. Specifically, the extra strain network significantly stabilizes elasticity estimation under noisy data. This novel architecture achieves strong robustness against noise in displacement and accurately reconstructs spatially varying elastic properties.

Furthermore, the IE‐PINN incorporates various strategies, including a positional encoding function, a sine activation function, and a pretraining strategy. We propose a two‐phase approach: Phase 1 involves estimating the spatial distribution of Young's modulus on a relative scale, along with Poisson's ratio; Phase 2 focuses on recovering the correct absolute scale of Young's modulus using defined loading boundary conditions.

IE‐PINN robustly achieves absolute‐scale elasticity estimation from noisy displacements, whereas state‐of‐the‐art methods exhibit significant performance degradation under noisy data. Our proposed approach is particularly advantageous for clinical elastography, material design, and structural optimization, where displacements are measured in response to defined external loading conditions and often contain noise arising from equipment or environmental factors.

## 4 Experimental Section

### Domain Knowledge of Linear Elasticity

Linear elasticity is a mathematical model that describes how solid objects deform and become internally stressed by prescribed loading conditions. The IE‐PINN model enables the estimation of spatially varying elastic properties from noisy displacement measurements by seamlessly embedding the governing physics laws of linear elasticity into the neural network loss function, thereby enforcing physical consistency during training. This study focuses on a two‐dimensional setting with isotropic material and the plane stress assumption. The formulation is based on three fundamental principles of linear elasticity: the displacement‐strain relation, the constitutive stress‐strain relation, and the static equilibrium equations, each detailed below.

First, the displacement‐strain relation characterizes material deformation within the framework of continuum mechanics. The displacement vector $\left(u={\left[u_{x},u_{y}\right]}^{T}\right)$ represents the movement of material points, while the strain vector $\left(\varepsilon ={\left[\varepsilon_{xx},\varepsilon_{yy},\gamma_{xy}\right]}^{T}\right)$ quantifies relative deformation. This relationship is represented by the system of partial differential equations (PDEs):

(1) $$\varepsilon =\left[\begin{array}{c} \varepsilon_{xx}\\ \varepsilon_{yy}\\ \gamma_{xy} \end{array}\right]=\left[\begin{array}{cc} \frac{\partial }{\partial x} & \;0\\ 0 & \;\frac{\partial }{\partial y}\\ \frac{\partial }{\partial y} & \;\frac{\partial }{\partial x} \end{array}\right]\left[\begin{array}{c} u_{x}\\ u_{y} \end{array}\right]$$

where $\varepsilon_{xx}$ and $\varepsilon_{yy}$ denote axial strains, $\gamma_{xy}$ is shear strain, and ∂/∂x and ∂/∂y denote partial derivatives.

Second, the elastic constitutive relation defines the relationship between stress and strain, characterized by Young's modulus (E) and Poisson's ratio (ν) at specific locations in a material. Stress, denoted by σ, quantifies internal force per unit area. Young's modulus reflects a material's local resistance to elastic (reversible) axial deformation, while Poisson's ratio quantifies the relationship between axial and transverse deformations. This relation is expressed as:

(2) $$\sigma =\left[\begin{array}{c} \sigma_{xx}\\ \sigma_{yy}\\ \tau_{xy} \end{array}\right]=\frac{E}{1-\nu^{2}}\left[\begin{array}{ccc} 1 & \;\nu & \;0\\ \nu & \;1 & \;0\\ 0 & \;0 & \;\frac{1-\nu }{2} \end{array}\right]\left[\begin{array}{c} \varepsilon_{xx}\\ \varepsilon_{yy}\\ \gamma_{xy} \end{array}\right]$$

where $\sigma_{xx}$ and $\sigma_{yy}$ are axial stresses, and $\sigma_{xy}$ is shear stress.

Third, the static equilibrium equations enforce pointwise force balance by setting the residual forces, defined as the divergence of the stress field, to zero at every point in space. The residual forces $\left(r_{x},r_{y}\right)$ are defined as follows.

(3) $$\begin{array}{cc} r_{x} & =\frac{\partial \sigma_{xx}}{\partial x}+\frac{\partial \tau_{xy}}{\partial y} \end{array}$$

(4) $$\begin{array}{cc} r_{y} & =\frac{\partial \tau_{xy}}{\partial x}+\frac{\partial \sigma_{yy}}{\partial y} \end{array}$$

The static equilibrium condition is enforced by setting the residual forces to zero:

(5) $$\begin{array}{c} r_{x}=0\;\text{and}\;\;r_{y}=0 \end{array}$$

### Finite Difference for Derivative Approximation

In the IE‐PINN framework, the finite difference method is utilized as the numerical differentiation technique to approximate derivatives.^[^ ^108^ ^]^ This method can be expressed using convolution operations with predefined derivative kernels on structured grids, which provides computational efficiency, enables GPU acceleration, and allows for seamless integration with deep learning frameworks.^[^ ^109^ ^]^ This operation is conducted across the entire two‐dimensional spatial domain by convolving the target field with predefined derivative kernels at each grid point (i,j), as detailed below.

(6) $$\begin{array}{c} \left(f*w\right)\left(i,j\right)=\sum_{a=1}^{A}\sum_{b=1}^{B}\left[w(a,b)\times f(i+a-1,j+b-1)\right] \end{array}$$

where f denotes the target field to be differentiated, while w is the (A×B) kernel corresponding to the specific partial derivative operation.

**Figure 18:**
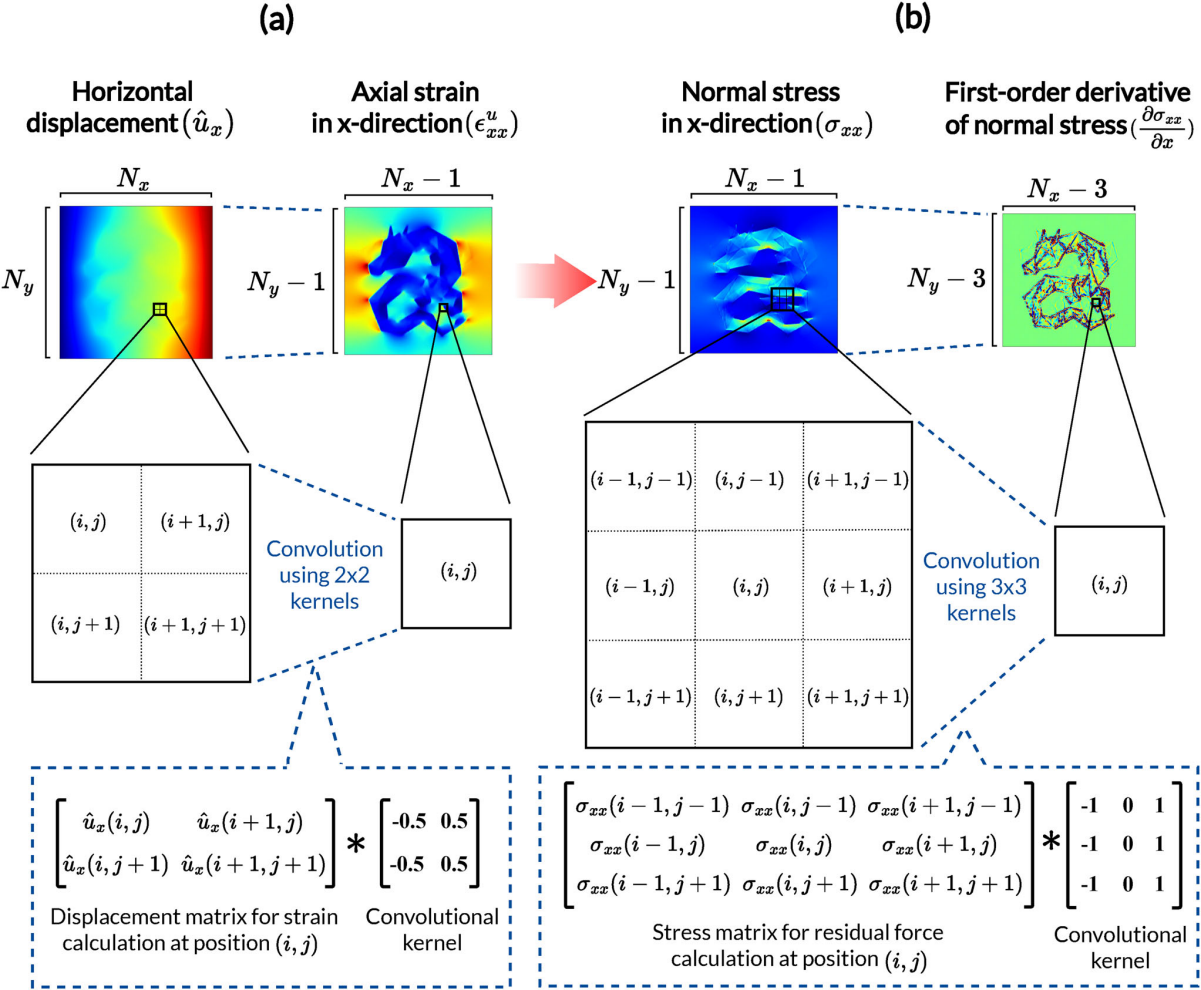
Finite difference approximation of derivatives. (a) Strains are computed using 2×2 finite difference kernels applied to displacement fields, based on the displacement‐strain equation. (b) First‐order derivative of an axial stress is computed using 3×3 kernels for static equilibrium equations.

The finite difference method is applied to compute both the displacement‐strain and static equilibrium equations. **Figure** **18**(a) illustrates how the axial strain in the x‐direction is calculated at position (i,j) using a 2×2 kernel $w_{x}$. Within each grid cell, the horizontal displacement field is convoluted with the kernel to compute the local axial strain. This operation is repeated across all grid cells to construct the strain map, resulting in dimension reduction from $N_{x}\times N_{y}$ to $\left(N_{x}-1\right)\times \left(N_{y}-1\right)$. The convolution operation for calculating strains is defined as follows.

(7) $$\varepsilon_{xx}^{u}\left(i,j\right)=\sum_{a=1}^{2}\sum_{b=1}^{2}w_{x}\left(a,b\right){\hat{u}}_{x}\left(i+a-1,j+b-1\right)$$

(8) $$\varepsilon_{yy}^{u}\left(i,j\right)=\sum_{a=1}^{2}\sum_{b=1}^{2}w_{y}\left(a,b\right){\hat{u}}_{y}\left(i+a-1,j+b-1\right)$$

(9) $$\begin{array}{ccc} \gamma_{xy}^{u}\left(i,j\right) & = & \sum_{a=1}^{2}\sum_{b=1}^{2}w_{y}\left(a,b\right){\hat{u}}_{x}\left(i+a-1,j+b-1\right)\\ & & +\;w_{x}\left(a,b\right){\hat{u}}_{y}\left(i+a-1,j+b-1\right) \end{array}$$

where the $w_{x}\left(i,j\right)$ and $w_{y}\left(i,j\right)$ are the convolution kernels that facilitate the finite difference of the displacements in x‐ and y‐direction, defined as follows.

(10) $$w_{x}=\left[\begin{array}{cc} -0.5 & \;0.5\\ -0.5 & \;0.5 \end{array}\right],\;w_{y}=\left[\begin{array}{cc} 0.5 & \;0.5\\ -0.5 & \;-0.5 \end{array}\right]$$

Residual forces are calculated by applying 3×3 convolutions to the stress field as illustrated in Figure 18(b). The dimension of the stress field reduces to $\left(N_{x}-3\right)\times \left(N_{y}-3\right)$. The residual forces at the position (i,j) are calculated as follows.

(11) $$\begin{array}{cc} r(i,j)= & \sum_{a=1}^{3}\sum_{b=1}^{3}\{w_{xx}\left(a,b\right)\sigma_{xx}\left(i+a-1,j+b-1\right)\\ & +w_{yy}\left(a,b\right)\sigma_{yy}\left(i+a-1,j+b-1\right)\\ & +w_{xy}\left(a,b\right)\tau_{xy}\left(i+a-1,j+b-1\right)\}/ht \end{array}$$

where $w_{xx}$, $w_{yy}$, and $w_{xy}$ are the kernels for the finite difference applied to the stress components $\sigma_{xx}$, $\sigma_{yy}$, and $\tau_{xy}$, respectively. h and t denote the vertical and horizontal spacing between neighboring displacement data points. $r_{x}$ is obtained by using the kernels defined below.

(12) $$\begin{array}{ccc} w_{xx} & = & \left[\begin{array}{ccc} -1 & \;0 & \;1\\ -1 & \;0 & \;1\\ -1 & \;0 & \;1 \end{array}\right],\;w_{yy}=\left[\begin{array}{ccc} 0 & \;0 & \;0\\ 0 & \;0 & \;0\\ 0 & \;0 & \;0 \end{array}\right],\\ w_{xy} & = & \left[\begin{array}{ccc} 1 & \;1 & \;1\\ 0 & \;0 & \;0\\ -1 & \;-1 & \;-1 \end{array}\right] \end{array}$$

and $r_{y}$ is obtained by the kernel below.

(13) $$\begin{array}{ccc} w_{xx} & = & \left[\begin{array}{ccc} 0 & \;0 & \;0\\ 0 & \;0 & \;0\\ 0 & \;0 & \;0 \end{array}\right],w_{yy}=\left[\begin{array}{ccc} 1 & \;1 & \;1\\ 0 & \;0 & \;0\\ -1 & \;-1 & \;-1 \end{array}\right],\\ w_{xy} & = & \left[\begin{array}{ccc} -1 & \;0 & \;1\\ -1 & \;0 & \;1\\ -1 & \;0 & \;1 \end{array}\right] \end{array}$$

### Architecture and Training of the IE‐PINN Framework

The IE‐PINN model estimates the spatial distributions of Young's modulus and Poisson's ratio that are expressed by neural networks. It is based on a hybrid approach integrating a data‐driven neural network model with the physics law of elasticity. As described in Section 2, IE‐PINN comprises three networks for predicting (i) displacements, (ii) elastic properties, and (iii) strains.

The displacement network predicts x‐ and y‐direction displacements field (${\hat{u}}_{x}\left(x,y\right)$ and ${\hat{u}}_{y}\left(x,y\right)$). These are denoised versions of the noisy observations $u^{*}\left(x_{i},y_{j}\right)$ collected at equidistant spatial positions $\left(x_{i},y_{j}\right)$, ($i=1,\text{…},N_{x}$ and $j=1,\text{…},N_{y}$). The elastic network predicts two output variables, Young's modulus and Poisson's ratio, denoted by $\hat{E}\left(x,y\right)$ and $\hat{\nu }\left(x,y\right)$, respectively. IE‐PINN estimates the underlying elastic properties by this elastic network. The trained elastic network recovers the unobserved Young's modulus and Poisson's ratio by learning functions that are consistent with the governing equations of linear elasticity. The strain network predicts three strain components: axial strains (${\hat{\varepsilon }}_{xx}\left(x,y\right)$ and ${\hat{\varepsilon }}_{yy}\left(x,y\right)$), and shear strain (${\hat{\tau }}_{xy}\left(x,y\right)$).

These three networks are seamlessly integrated through a specially defined loss function. This loss function is designed to achieve four objectives: minimizing the prediction discrepancies of (i) displacements, (ii) strains, (iii) PDE equilibrium residuals, and (iv) elastic modulus constraints from their ideal values. A common strategy for this multi‐objective optimization problem is to minimize the overall loss, represented as a weighted sum of four individual loss terms:

(14) $$\min_{\theta_{u},\theta_{\varepsilon },\theta_{E}}L_{\text{total}}=\min_{\theta_{u},\theta_{\varepsilon },\theta_{E}}\left[\lambda_{u}L_{u}+\lambda_{\varepsilon }L_{\varepsilon }+\lambda_{r}L_{r}+\lambda_{E}L_{E}\right]$$

where $L_{s}$ and $\lambda_{s}$ denote the loss term and its corresponding weight for each $s\in \left\{u,\varepsilon ,r,E\right\}$. In this work, the loss weights are selected based on empirical exploration of different combinations. A detailed description is included in Note S4 (Supporting Information). Equation (14) identifies the optimal network parameters ($\theta_{u}$ for displacement, $\theta_{\varepsilon }$ for strain, $\theta_{E}$ for elasticity) that jointly satisfy the four loss criteria. The L1 norm is used to measure deviations from the ideal values in all loss terms.

The displacement loss $L_{u}$ measures the average prediction error between the predicted displacements and the observed noisy measurements in spatial directions:

(15) $$L_{u}=\frac{1}{N_{x}N_{y}}\sum_{i=1}^{N_{x}}\sum_{j=1}^{N_{y}}\left(|{\hat{u}}_{x}\left(i,j\right)-u_{x}^{*}\left(i,j\right)|+|{\hat{u}}_{y}\left(i,j\right)-u_{y}^{*}\left(i,j\right)|\right)$$

where $N_{x}$ and $N_{y}$ denote the numbers of x‐ and y‐coordinates in the spatial grid where noisy displacement observations $u_{x}^{*}$ and $u_{y}^{*}$ are collected. ${\hat{u}}_{x}$ and ${\hat{u}}_{y}$ denote the corresponding predicted displacements.

The strain loss $L_{\varepsilon }$ quantifies the discrepancy between the strain predicted by the strain network ($\hat{\varepsilon }$ and $\hat{\gamma }$), and the strain computed from the displacement network via the finite difference ($\varepsilon^{u}$ and $\gamma^{u}$):

(16) $$\begin{array}{ccc} L_{\varepsilon } & = & \frac{1}{\left(N_{x}-1\right)\left(N_{y}-1\right)}\sum_{i=1}^{N_{x}-1}\sum_{j=1}^{N_{y}-1}\left(\right|{\hat{\varepsilon }}_{xx}\left(i,j\right)-\varepsilon_{xx}^{u}\left(i,j\right)\left|+\right|{\hat{\varepsilon }}_{yy}\left(i,j\right)\\ & & -\;\varepsilon_{yy}^{u}\left(i,j\right)|+|{\hat{\gamma }}_{xy}\left(i,j\right)-\gamma_{xy}^{u}\left(i,j\right)\left|\right) \end{array}$$

Here, the reduced spatial resolution, $\left(N_{x}-1\right)$ and $\left(N_{y}-1\right)$, results from the finite difference.

The physical laws of linear elasticity are enforced through the PDE residual loss, which penalizes deviations from static equilibrium. The stress field is computed via the constitutive equation in (2), using the predicted strain and elasticity values. The PDE residuals ($r_{x}\left(i,j\right)$ and $r_{y}\left(i,j\right)$) are computed through (11), (12), and (13). The PDE residual loss is defined as follows.

(17) $$L_{r}=\frac{1}{\left(N_{x}-3\right)\left(N_{y}-3\right)}\sum_{i=1}^{N_{x}-3}\sum_{j=1}^{N_{y}-3}\frac{|r_{x}\left(i,j\right)|+|r_{y}\left(i,j\right)|}{\tilde{E}\left(i,j\right)}$$

The denominator $\tilde{E}\left(i,j\right)$ is a local aggregation of predicted Young's modulus values for normalization, defined by:

(18) $$\tilde{E}\left(i,j\right)=\sum_{a=1}^{3}\sum_{b=1}^{3}\hat{E}\left(i+a-1,j+b-1\right)$$

To determine the absolute scale of Young's modulus, a boundary condition must be considered. In Phase 1, IE‐PINN estimates the relative distribution of Young's modulus ($\hat{E}\left(i,j\right)$) by constraining its mean to a user‐defined reference value, $E_{c}$. The corresponding loss function is given by:

(19) $$L_{E}=\left|\frac{1}{\left(N_{x}-1\right)\left(N_{y}-1\right)}\sum_{i=1}^{N_{x}-1}\sum_{j=1}^{N_{y}-1}\hat{E}\left(i,j\right)-E_{c}\right|$$

Although this constraint centers the modulus distribution around an arbitrary scale, Phase 2 applied an external loading boundary condition to calibrate and recover the absolute scale.

As summarized in Figure 1, the three networks are seamlessly integrated such that the PDE residuals are computed from the noisy displacement measurements through the interconnection of the networks and their associated loss functions. During training, all neural network parameters are updated simultaneously to minimize the total loss function. In IE‐PINN, a dedicated strain network is introduced specifically for stress calculation, decoupling the PDE residual calculation from direct reliance on the displacement network. This decoupling significantly mitigates the sensitivity to noise, particularly in the computation of the second‐order derivatives. The training followed the pretraining strategy outlined in Section 2.7.

The hyperparameters used during the experiments are detailed in Table S3 (Supporting Information). Additionally, the computational consumptions assessed with a single GPU (NVIDIA RTX A2000) are summarized in Table S4 (Supporting Information).

The estimation error among the same dataset is measured by the mean absolute error (MAE), whereas the mean relative error (MRE) is used to compare the estimation error across different data sets. Both measures are defined as

(20) $$MAE=\frac{1}{\left(N_{x}-1\right)\left(N_{y}-1\right)}\sum_{i=1}^{N_{x}-1}\sum_{j=1}^{N_{y}-1}\left(|\hat{E}\left(i,j\right)-E\left(i,j\right)|\right)$$

(21) $$MRE=\frac{100}{\left(N_{x}-1\right)\left(N_{y}-1\right)}\sum_{i=1}^{N_{x}-1}\sum_{j=1}^{N_{y}-1}\left(\frac{|\hat{E}\left(i,j\right)-E\left(i,j\right)|}{E(i,j)}\right)$$

where $\hat{E}$ denotes the estimated Young's modulus, and E denotes the ground‐truth modulus. Poisson's ratio is evaluated in the same manner.

### Young's Modulus Scale Calibration Technique

The inverse elasticity problem is generally ill‐posed; estimation from displacement is unstable. The boundary condition is theoretically required to obtain the absolute value of Young's modulus. The existing methods use 1) known internal stress distributions, 2) known boundary stress distributions, or 3) impose the true mean Young's modulus loss to guarantee a unique solution to the problem; however, the obtained solution is a relative Young's modulus with respect to the constrained mean of Young's modulus value. Moreover, the mean of Young's modulus is always unknown.

The scale calibration technique is proposed to recover the absolute Young's modulus field from the relative stress field. This approach relies on determining the absolute scale of Young's modulus by utilizing the Neumann boundary condition known as the traction boundary condition.^[^ ^110^, ^111^ ^]^ This condition indicates the resultant of surface force $\left(F_{S}\right)$ over the entire boundary surface S that can be expressed as the integral of a surface force density function $T^{n}\left(x\right)$.

(22) $$F_{S}=\int \int_{(x,y)\in S}T^{n}\left(x,y\right)dxdy$$

Commonly, the surface force density is referred to as the traction vector that varies with the spatial location. In this study, which focuses on a thin rectangular plate with a plane stress assumption, the geometry of the object is assumed to be two‐dimensional, where the traction is defined as follows.^[^ ^111^ ^]^

(23) $$T^{n}\left(x,y\right)=\left[\begin{array}{c} T_{x}^{\left(b\right)}\left(x,y\right)\\ T_{y}^{\left(b\right)}\left(x,y\right) \end{array}\right]=\left[\begin{array}{c} \sigma_{xx}^{\left(b\right)}\left(x,y\right)n_{x}+\tau_{xy}^{\left(b\right)}\left(x,y\right)n_{y}\\ \tau_{xy}^{\left(b\right)}\left(x,y\right)n_{x}+\sigma_{yy}^{\left(b\right)}\left(x,y\right)n_{y} \end{array}\right]$$

where $T_{x}^{\left(b\right)}$ and $T_{y}^{\left(b\right)}$ are the traction force at the boundary in the x‐ and y‐directions, $\sigma_{xx}^{\left(b\right)}$ and $\sigma_{yy}^{\left(b\right)}$ are the stress at boundary in the x‐ and y‐directions, $\tau_{xy}^{\left(b\right)}$ is a shear stress at boundary, $n_{x}$ and $n_{y}$ are normal vector to x‐ and y‐directions

In the simulated dataset used in this study, a boundary force is applied in the x‐direction, acting perpendicular to the right surface of the object. A detailed description of the loading boundary condition is included in Note S5 (Supporting Information). The corresponding traction force per spatial coordinate is then as follows.

(24) $$T^{n}\left(X\right)=T_{x}^{\left(b\right)}\left(x,y\right)=\sigma_{xx}^{\left(b\right)}\left(x,y\right)n_{x}+\tau_{xy}^{\left(b\right)}\left(x,y\right)n_{y}$$

As the applied force is perpendicular to the surface, it can be written as the vector form with $Fn_{x}+0n_{y}$. Then, the boundary force F is equivalent to the total traction force at the boundary in the x‐direction that can be calculated from the predicted stress at the boundary as

(25) $$\begin{array}{c} F={\;\int \int \;}_{(x,y)\in S}\sigma_{xx}^{\left(b\right)}\left(x,y\right)dxdy \end{array}$$

In general, any function E(x,y) can always be written as $cE^{\prime }\left(x,y\right)$, where c is defined as a scale magnitude. If the Young's modulus E(x,y) in the linear elasticity model satisfies the static equilibrium equation in (3) and (4), $E^{\prime }\left(x,y\right)$ also satisfies the equation for any c.

Therefore, without the inclusion of loading boundary conditions, E(x,y) remains unidentifiable from these equilibrium equations alone. One common approach to anchor the scale of $E^{\prime }\left(x,y\right)$, and thereby determine c, is to constrain the mean of $E^{\prime }\left(x,y\right)$. In our proposed IE‐PINN framework, this is achieved by enforcing the mean of elastic modulus constraint ${\bar{E}}^{\prime }\left(x^{\prime },y^{\prime }\right)=E_{c}$ within the loss function. We denote the trained $E^{\prime }\left(x,y\right)$ in IE‐PINN as $\hat{E}\left(x,y\right)$. Since $E_{c}$ is arbitrary, the resulting scale of $\hat{E}\left(x,y\right)$ cannot be interpreted in absolute terms; thus, it exists on a relative scale.

The true magnitude c can be recovered by aligning $c\hat{E}\left(x,y\right)$ with the loading boundary condition in Equation (25). The stress component $\sigma_{xx}$ can be expressed as $\sigma_{xx}=c\sigma_{xx}^{\prime }$, as demonstrated below through the constitutive relation:

(26) $$\begin{array}{cc} \sigma & =\frac{E}{1-\nu^{2}}\left[\begin{array}{ccc} 1 & \;\nu & \;0\\ \nu & \;1 & \;0\\ 0 & \;0 & \;\frac{1-\nu }{2} \end{array}\right]\left[\begin{array}{c} \varepsilon_{xx}\\ \varepsilon_{yy}\\ \gamma_{xy} \end{array}\right] \end{array}$$

(27) $$\begin{array}{cc} & =\frac{\hat{c}\hat{E}}{1-\nu^{2}}\left[\begin{array}{ccc} 1 & \;\nu & \;0\\ \nu & \;1 & \;0\\ 0 & \;0 & \;\frac{1-\nu }{2} \end{array}\right]\left[\begin{array}{c} \varepsilon_{xx}\\ \varepsilon_{yy}\\ \gamma_{xy} \end{array}\right] \end{array}$$

(28) $$\begin{array}{cc} & =\hat{c}\hat{\sigma } \end{array}$$

Equation (25) can therefore be rewritten as:

(29) $$\begin{array}{c} F={\;\int \int \;}_{(x,y)\in S}\hat{c}{\hat{\sigma }}_{xx}^{\left(b\right)}\left(x,y\right)dxdy \end{array}$$

By using numerical integration, the boundary force is obtained as follows.

(30) $$\begin{array}{cc} F & \approx \sum_{i=0}^{Y}\hat{c}{\hat{\sigma }}_{xx}\left(x_{b},y_{i}\right))h \end{array}$$

The estimated relative Young's modulus is subsequently calibrated to recover the absolute Young modulus. The formulation of this absolute scale calibration is given by:

(31) $$\begin{array}{c} \hat{c}=\frac{F}{\sum_{i=0}^{Y}{\hat{\sigma }}_{xx}\left(x_{b},y_{i}\right)h} \end{array}$$

and absolute Young's modulus, denoted by $E_{absolute}\left(x,y\right)$, is obtained by scaling the predicted relative field $\hat{E}\left(x,y\right)$ with a calibration factor ($\hat{c}$):

(32) $$\begin{array}{cc} E_{absolute}\left(x,y\right) & =\hat{c}\hat{E}\left(x,y\right) \end{array}$$

### Data Descriptions

The datasets for simulation are generated from the published data in the work by Chen and Gu.^[^ ^53^, ^75^ ^]^ The Gaussian noise, which is commonly used to verify robustness against noise, is incorporated into the displacement field generated from finite element analysis (FEA), denoted as u(i,j), using the following formulation:^[^ ^59^, ^84^, ^85^, ^86^ ^]^

(33) $$\begin{array}{c} u_{g}^{*}\left(i,j\right)=u\left(i,j\right)+\varepsilon_{i,j} \end{array}$$

where $u_{g}^{*}\left(i,j\right)$ represents the observed noisy displacement data with the Gaussian noise. ε denotes a zero‐mean Gaussian noise with a standard deviation (σ), which is determined according to a prespecified signal‐to‐noise ratio (SNR) as follows:

(34) $$\begin{array}{c} \sigma =\left|\frac{\bar{u}}{SNR}\right| \end{array}$$

where $\bar{u}$ is the mean of the displacement measurements. To evaluate the robustness of the proposed method, SNR levels are systematically varied from 1000 to 20.

For example, the primary dataset used in the manuscript is a displacement field generated from FEA based on the spatial distribution of Young's modulus, shaped as a dragon, and that of Poisson's ratio, shaped as a dog. In this dataset, the mean horizontal displacement ($u_{x}$) is 1.27 and the mean vertical displacement ($u_{y}$) is 0.242. For an SNR of 100, Gaussian noise with standard deviations of 0.0127 and 0.00242 is added to the horizontal and vertical displacements, respectively.

Recognizing the limitations of independent Gaussian noise, we also perform additional validation with structured noise following established models,^[^ ^90^ ^]^. The observed noisy displacement with structured noise $u_{s}^{*}\left(i,j\right)$ is represented as

(35) $$\begin{array}{c} u_{s}^{*}\left(i,j\right)=u\left(i,j\right)*h\left(i,j\right)+\gamma \left(i,j\right) \end{array}$$

where h(i,j) denotes a spatially dependent point spread function (PSF), and γ(i,j) is an additive Gaussian noise term with standard deviation $\sigma_{\gamma }$ that is also varied according to the target SNR levels, similar to the Gaussian noise scenario. Structured noise is modeled by the convolution of u(i,j) with the PSF, which is often represented using a Gaussian kernel modulated by a cosine function, as shown below.^[^ ^112^ ^]^

(36) $$\begin{array}{c} h\left[i,j\right]=e^{-\frac{i^{2}}{\sigma_{x}^{2}}-\frac{j^{2}}{\sigma_{y}^{2}}}\cos\;\left(2\pi f_{c}j\right) \end{array}$$

where $f_{c}$ is the transducer center frequency (6 MHz), and $\sigma_{x}^{2}$ and $\sigma_{y}^{2}$ are the axial and vertical spatial spreading, set at 0.5 and 0.05, respectively, as reported in the literature.^[^ ^90^ ^]^

### Neural Network Architecture

All three neural networks (a displacement, a strain, and an elasticity network) in IE‐PINN have 16 fully connected hidden layers with 128 neurons. The activation function between each fully connected hidden layer is a sine activation function named “SIREN”.^[^ ^92^ ^]^ Only the elasticity network has the last layer as the softplus activation function to guarantee the positivity of prediction. In all three networks, the input coordinates will pass through a positional encoding layer to achieve a more meaningful representation for learning. In this study, the sine and cosine functions with different frequencies,^[^ ^95^ ^]^ are used as the positional encoding function at the input layer defined as

(37) $$\begin{array}{cc} \gamma (x,2i) & =\sin\left(f^{2i/\omega }x\right),\;\;\;i\in \left\{1,2,\cdots ,\omega \right\} \end{array}$$

(38) $$\begin{array}{cc} \gamma (x,2j+1) & =\cos\left(f^{2j/\omega }x\right),\;\;\;j\in \left\{1,2,\cdots ,\omega \right\} \end{array}$$

where x is the x coordinate, and the positional encoding in (37) and (38) is also employed to y positional input. f is the minimum frequency set at 0.0001, and ω is the maximum number of frequencies set at 64 in this work.

## Conflict of Interest

The authors declare no conflict of interest.

## Supporting information

Supporting Information

## Data Availability

The data that support the findings of this study are available in the supplementary material of this article.
