# Supplementary material for: Robust Physics‐Informed Neural Network Approach for Estimating Heterogeneous Elastic Properties from Noisy Displacement Data
Source: Adv Sci (Weinh). 2025 Nov 25;12(48):e08445. doi: 10.1002/advs.202508445 (PMC12752583; doi:10.1002/advs.202508445)
Supplement: Supplementary file 1 — Supporting Information [file ADVS-12-e08445-s001.pdf]

Supporting Information

Robust Physics-Informed Neural Network Approach for Estimating Heterogeneous Elastic Properties from Noisy Displacement Data

Tatthapong Srikitrungruang, Sina Aghaee Dabaghan Fard, Matthew Lemon, Jaesung Lee, Yuxiao Zhou*

Supplementary Figures

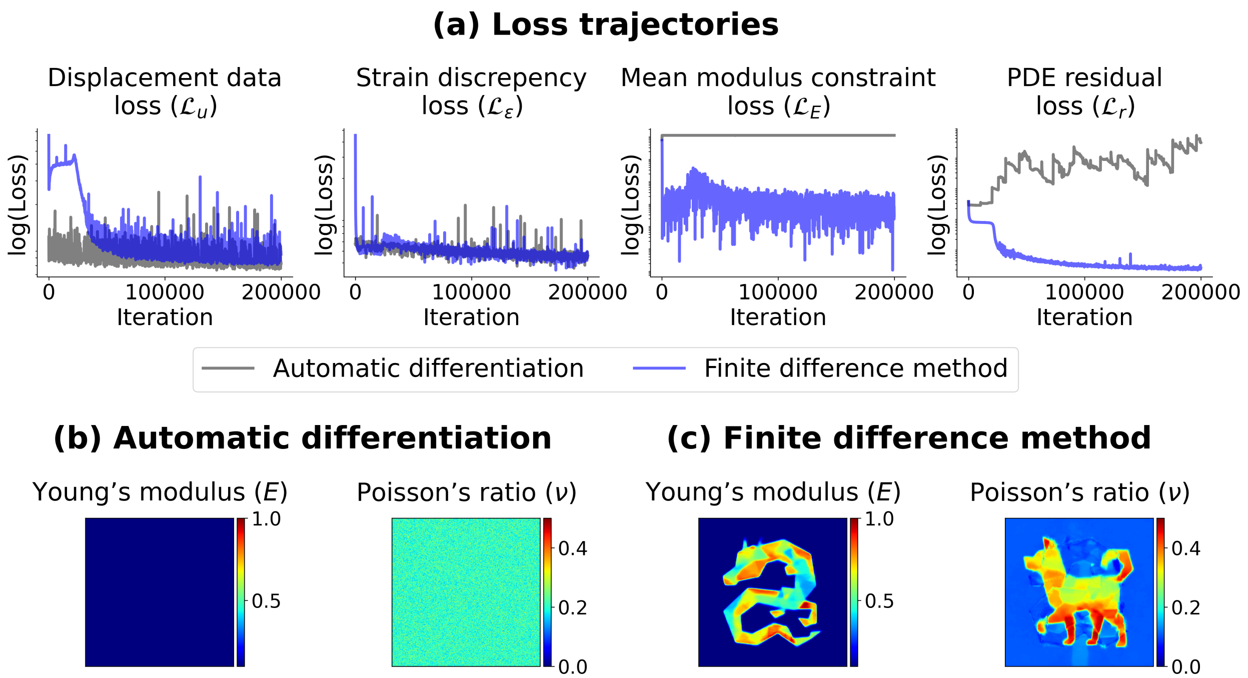

Supplementary Figure S1: Comparison between automatic differentiation and finite difference method . Both differentiation methods were applied using the same parameters on the same dataset. The results include (a) Loss trajectories of two different approaches, (b) Elasticity estimations obtained through automatic differentiation, and (c) Elasticity estimations obtained using finite difference method . In the context of the inverse elasticity problem, automatic differentiation fails to estimate the elasticity parameters.

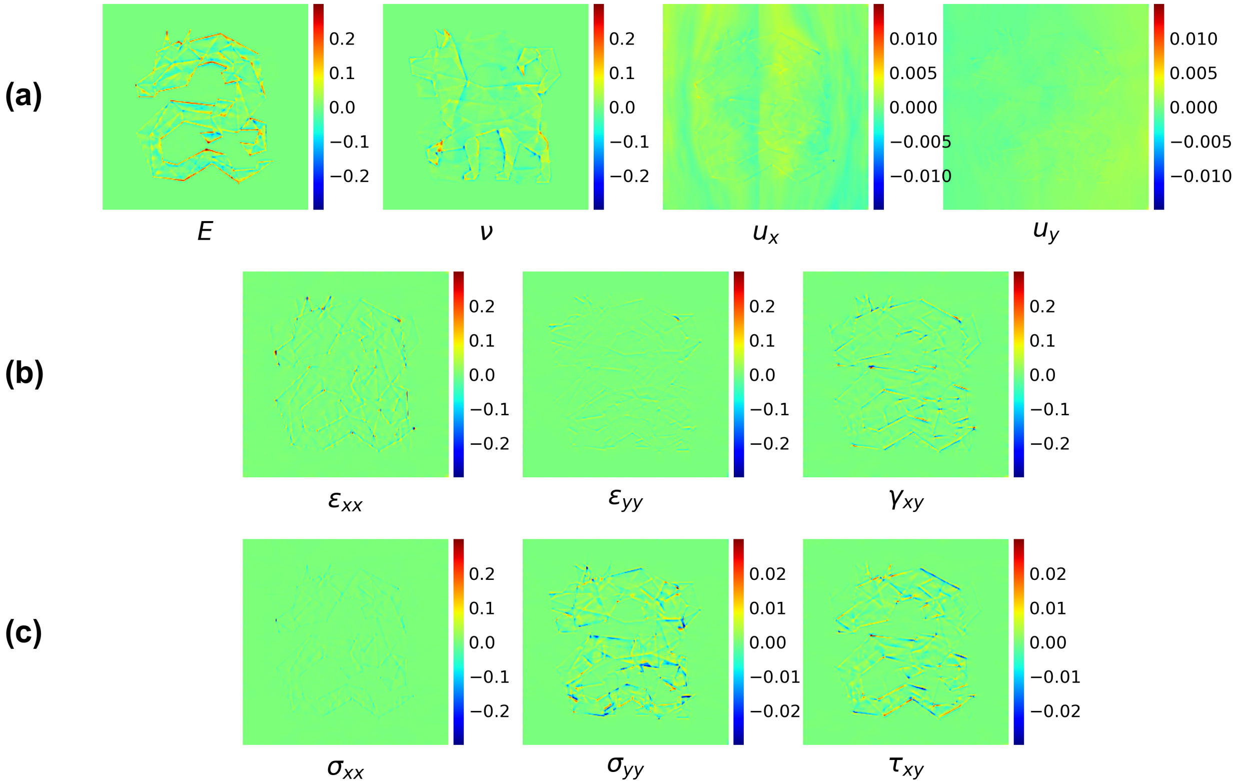

Supplementary Figure S2: The error map of mechanical quantities. The model is applied with a measured displacement that contains a signal-to-noise ratio of 1000. (a) The error of predicted Young's modulus field (MPa), Poisson's ratio field, and axial displacement field (mm) (b) The error map of predicted strain field (%) (c) The error map of predicted stress field (MPa)

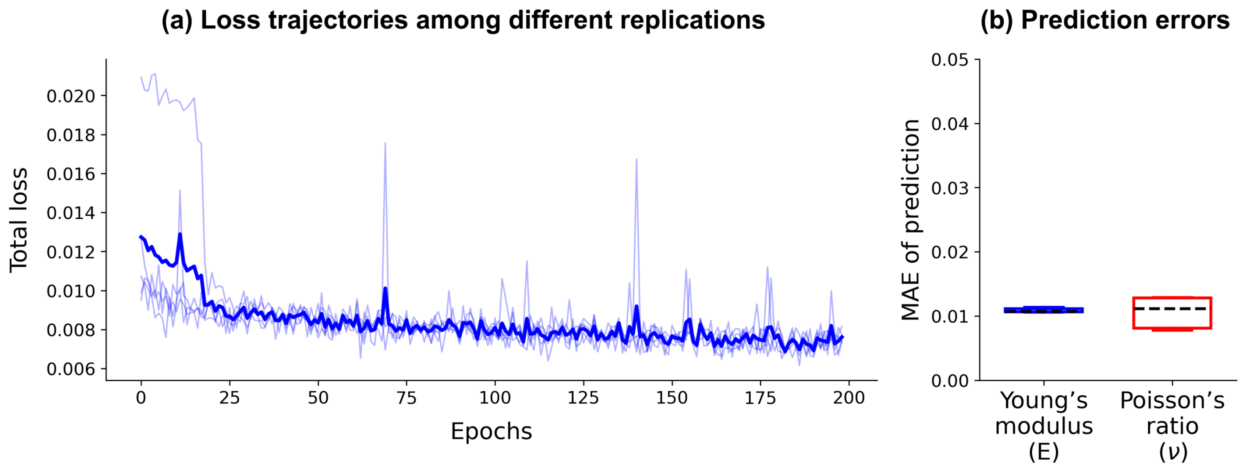

Supplementary Figure S3: Stability of IE-PINN. (a) Loss trajectories across five replications on the same dataset with noisy displacement data (SNR of 1000), demonstrating convergence to a unique solution. (b) Elasticity estimation errors across replications. The MAE exhibits only slight variation, confirming the stability and reproducibility of the predicted elasticity parameters.

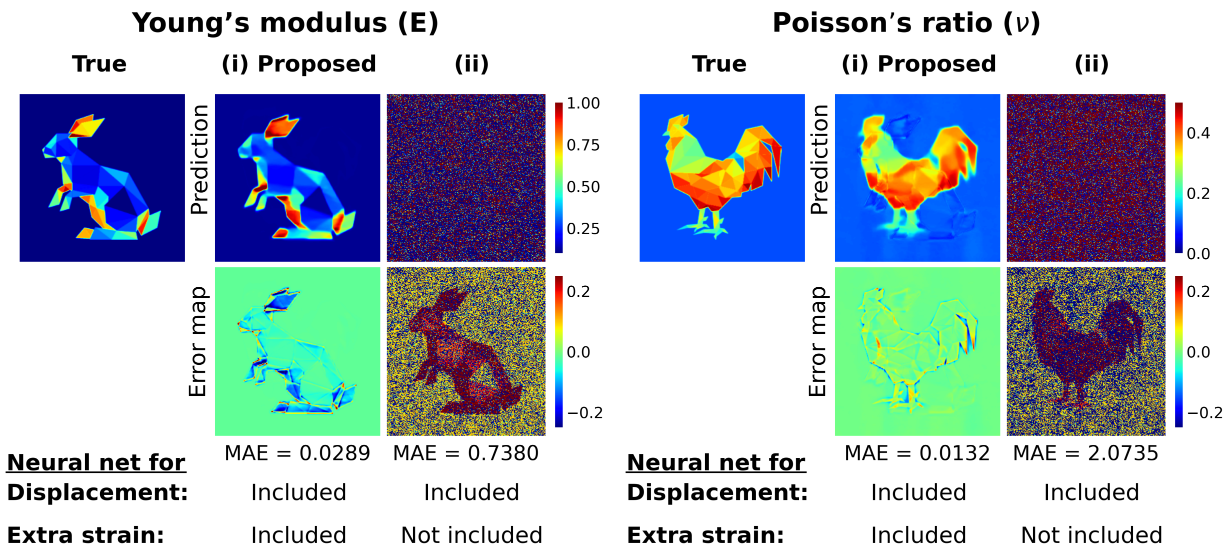

(a) Estimated Young's modulus across different models. **(b)** Estimated Poisson's ratio across different models.

Supplementary Figure S4: Young's modulus (E) and Poisson's ratio (ν) estimation and error map of different models. The models were trained using the same noisy displacement data with a signal-to-noise ratio of 1000, where the true spatial distribution of Young's modulus has the shape of a rabbit, and that of Poisson's ratio has the shape of a roaster. (i) IE-PINN (Proposed) includes both displacement and strain networks. (ii) Model only with displacement network (without strain network). In this dataset, using only displacement networks fails in elasticity estimation.

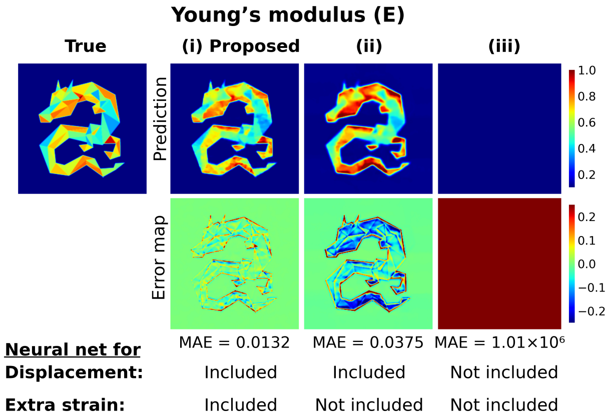

(a) Estimated Young's modulus across different models.

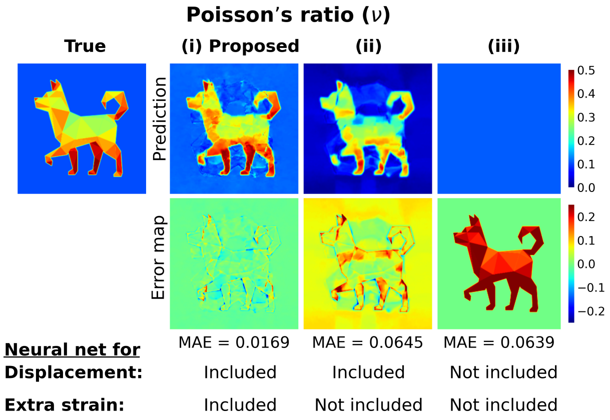

(b) Estimated Poisson's ratio across different models.

Supplementary Figure S5: Young's modulus (E) and Poisson's ratio (ν) estimation and error map of different models at a signal-to-noise ratio of 500. The models were trained using the displacement data with a higher noise level: SNR 500. (i) IE-PINN (Proposed): Model that fits in both displacement and strain. (ii) Model with fitting only displacement data without extra strain fitting and (iii) ElastNet : Model without fitting in both displacement and strain data.[1] When the noise increases, relying solely on fitting displacement is less effective than additional fitting strain.

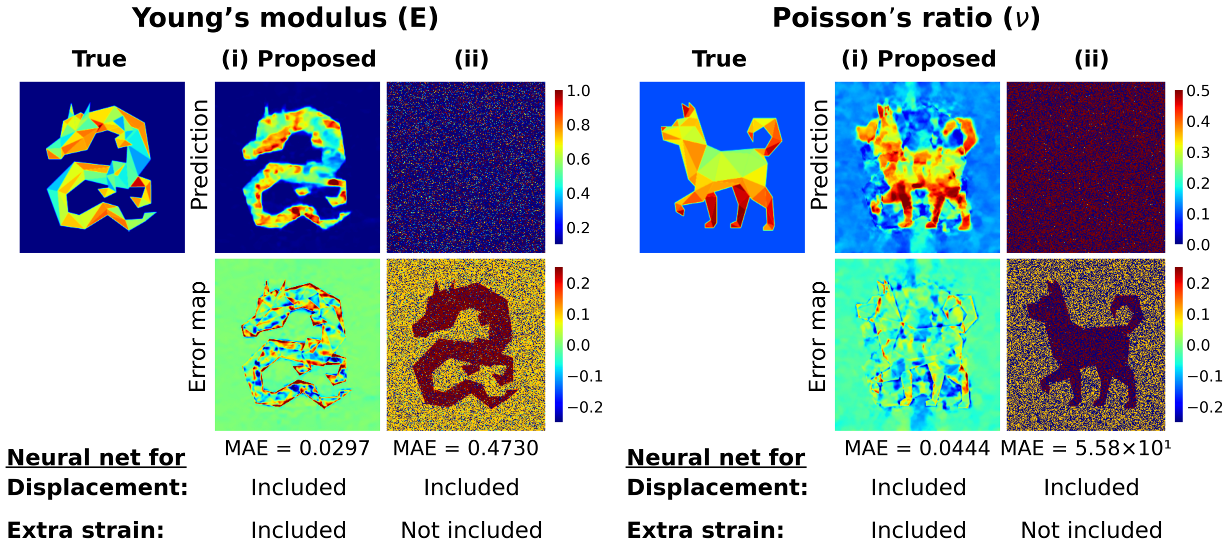

(a) Estimated Young's modulus across different models. **(b)** Estimated Poisson's ratio across different models.

Supplementary Figure S6: Young's modulus (E) and Poisson's ratio (ν) estimation and error map of different models at a signal-to-noise ratio of 100. The models were trained using the displacement data with a higher noise: SNR 100. (i) IE-PINN (Proposed): Model that fits in both displacement and strain. (ii) Model with fitting only displacement data without extra strain fitting, and the strain can be calculated from the displacement-strain relation through finite differentiation. In conditions of high noise, fitting displacement only is not able to resist the high noise condition and leads to worse elasticity estimation.

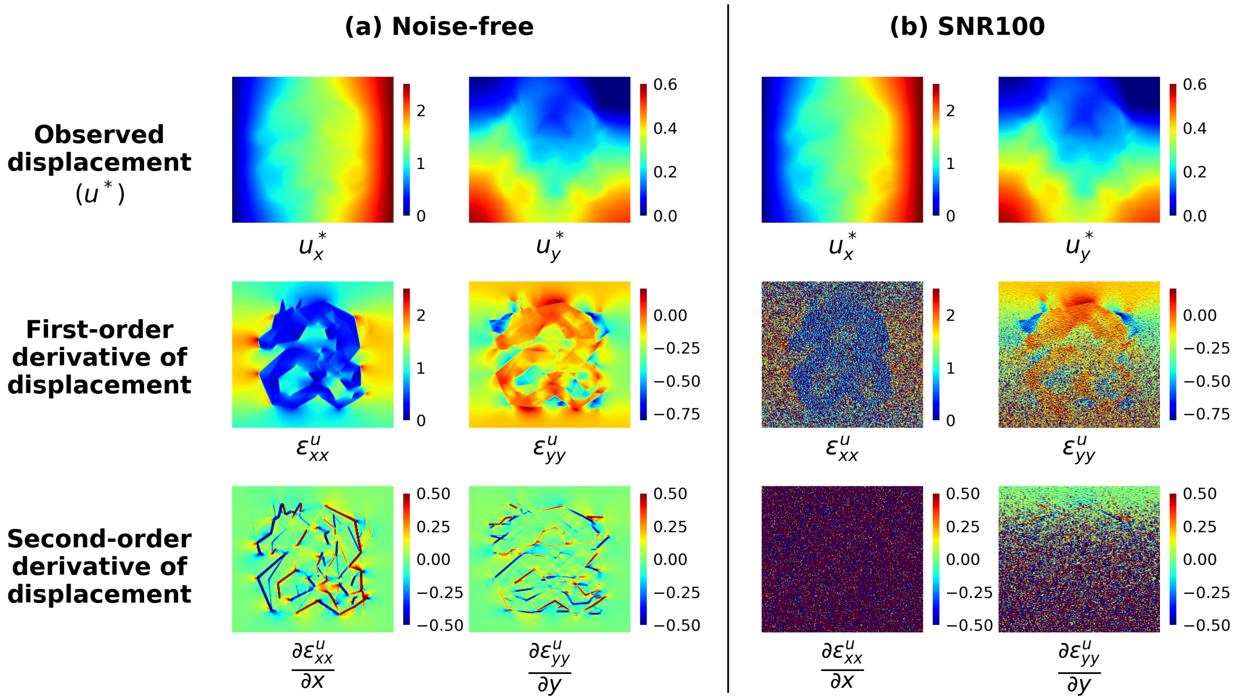

Supplementary Figure S7: Effects of noise on derivative computation when directly applying the finite difference. The presence of noise significantly impacts derivative accuracy, as illustrated by comparing the results obtained using (a) noise-free displacement and (b) noisy displacement at SNR 100. The derivative field derived from the noise-free displacement data clearly reveals the dragon shape. In contrast, the derivative field obtained from noisy data exhibits significant noise amplification during differentiation. Furthermore, the second-order derivatives computed from noisy displacement data, which are critical in evaluating partial differential equation (PDE) equilibrium residuals, appear completely corrupted and show no discernible structure.

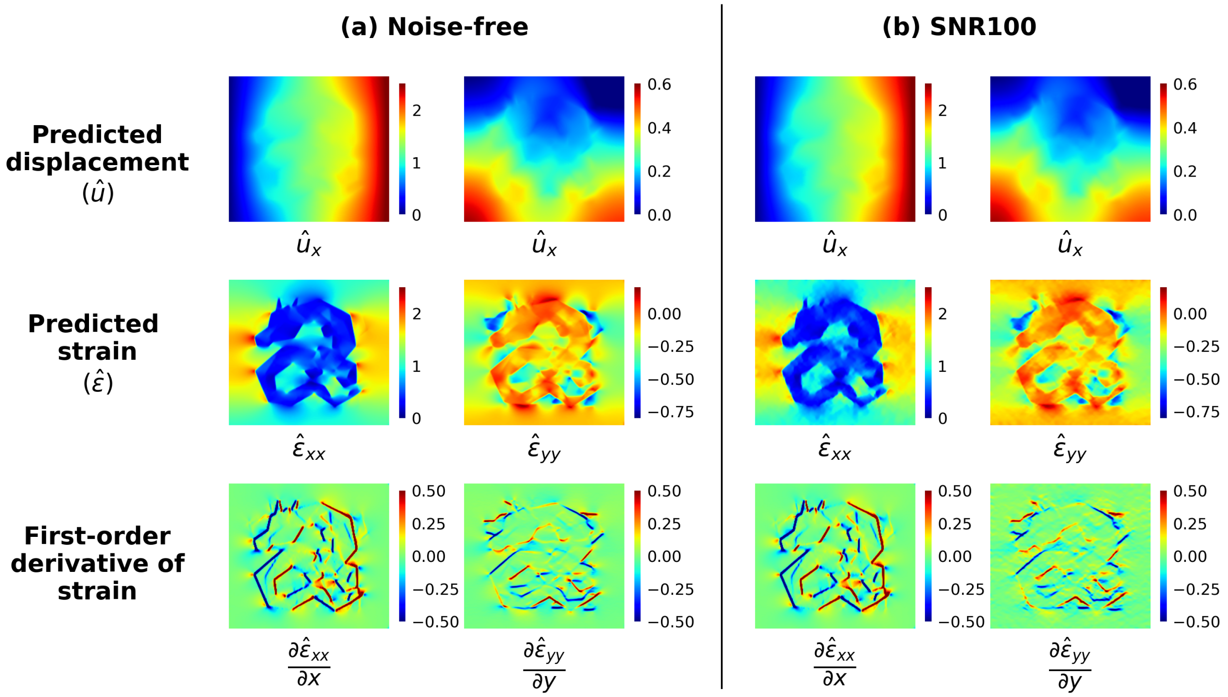

Supplementary Figure S8: Effects of noise on derivative prediction from IE-PINN. The predicted strain obtained from IE-PINN is similar in both (a) the noise-free case and (b) the noisy observation case. In both scenarios, IE-PINN successfully recovers both the first- and second-order derivatives, demonstrating its robustness to noise.

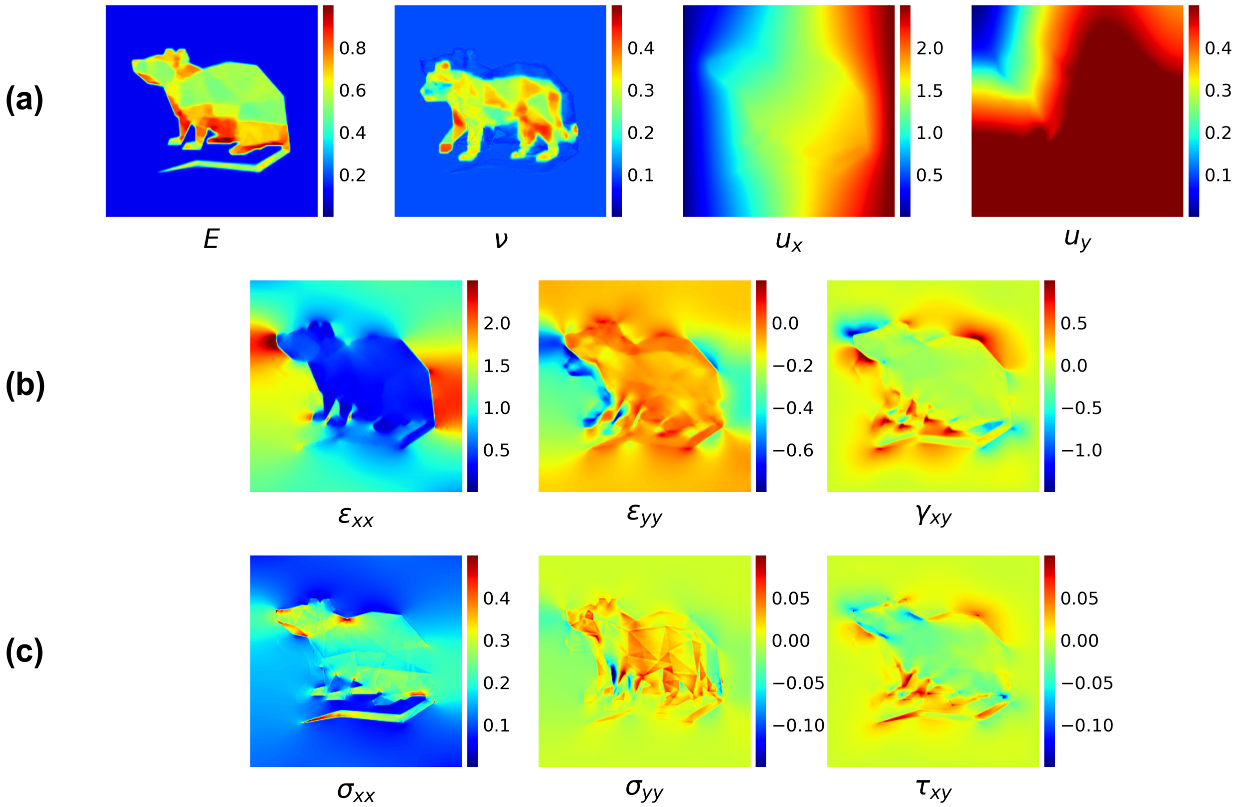

Supplementary Figure S9: The predicted elastic properties based on the rat-shaped distribution of Young's modulus and the tiger-shaped distribution of Poisson's ratio. The model uses the noisy displacement field at a signal-to-noise ratio (SNR) of 1000. (a) The predicted Young's modulus field, Poisson's ratio field, and axial displacement field. (b) The predicted strain field (%). (c) The predicted stress field.

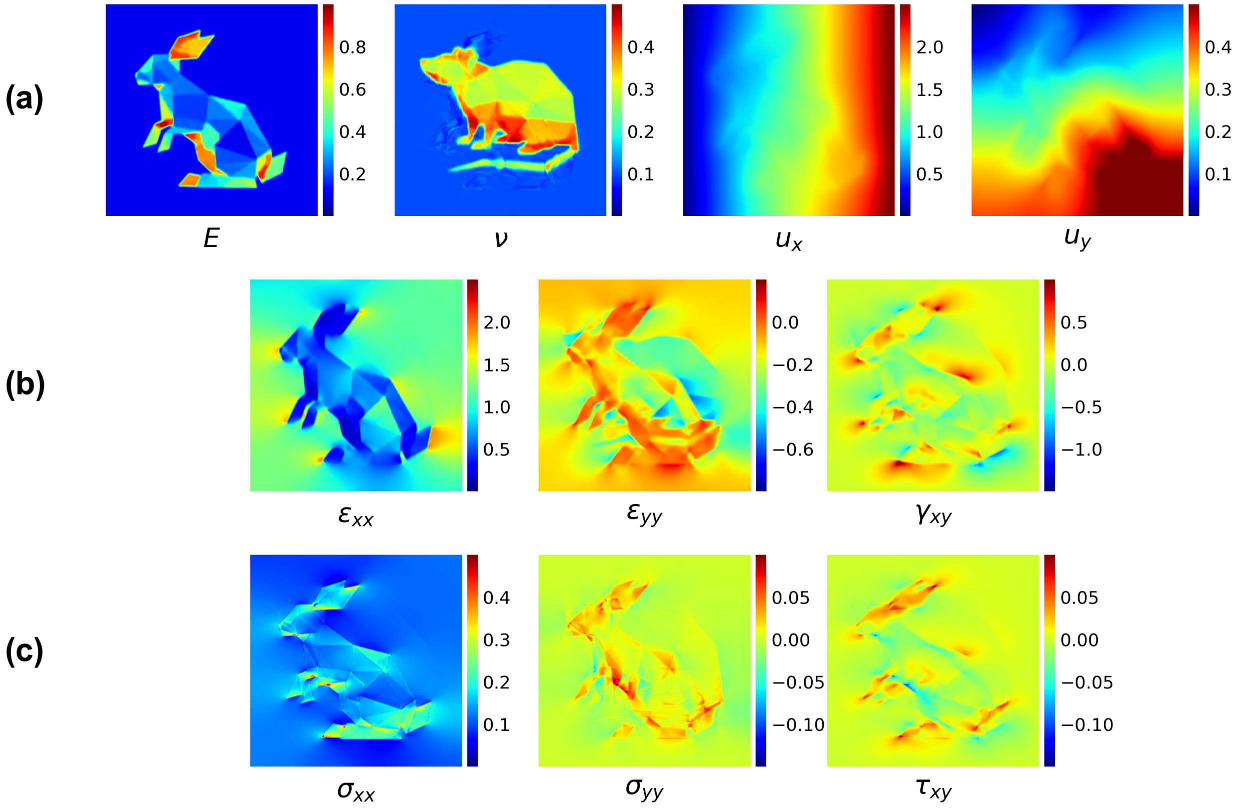

Supplementary Figure S10: The predicted elastic properties based on the rabbit-shaped distribution of Young's modulus and the rat-shaped distribution of Poisson's ratio. The model uses the noisy displacement field at a signal-to-noise ratio (SNR) of 1000. (a) The predicted Young's modulus field, Poisson's ratio field, and axial displacement field. (b) The predicted strain field (%). (c) The predicted stress field.

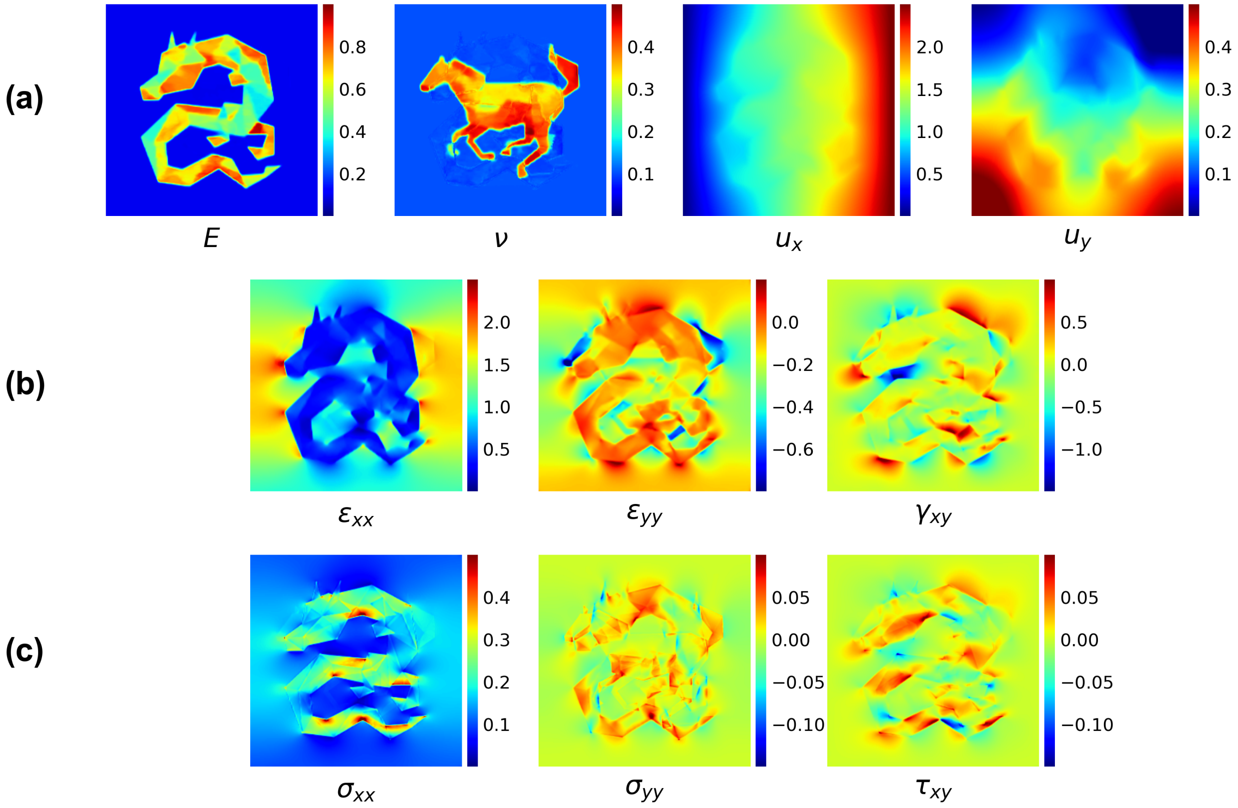

Supplementary Figure S11: The predicted elastic properties based on the dragon-shaped distribution of Young's modulus and the horse-shaped distribution of Poisson's ratio. The model uses the noisy displacement field at a signal-to-noise ratio (SNR) of 1000. (a) The predicted Young's modulus field, Poisson's ratio field, and axial displacement field. (b) The predicted strain field (%). (c) The predicted stress field.

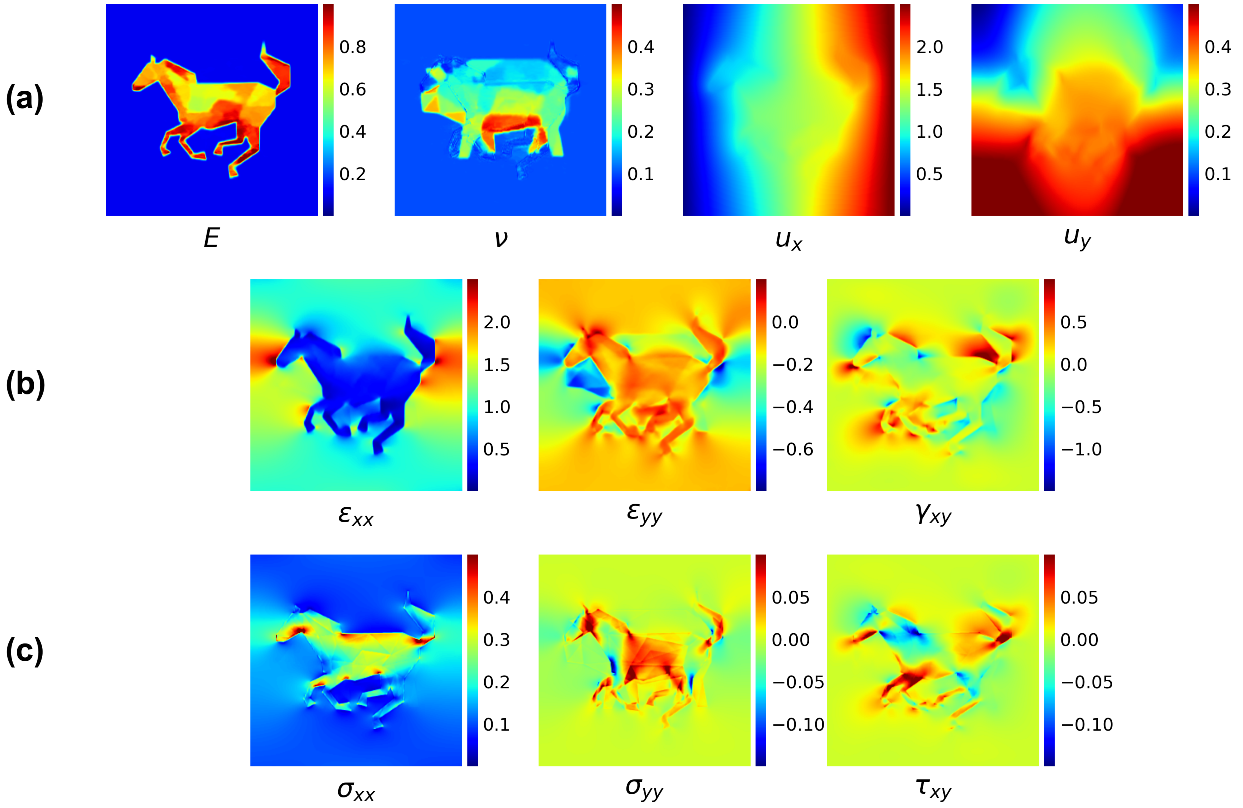

Supplementary Figure S12: The predicted elastic properties based on the horse-shaped distribution of Young's modulus and the pig-shaped distribution of Poisson's ratio. The model uses the noisy displacement field at a signal-to-noise ratio (SNR) of 1000. (a) The predicted Young's modulus field, Poisson's ratio field, and axial displacement field. (b) The predicted strain field (%). (c) The predicted stress field.

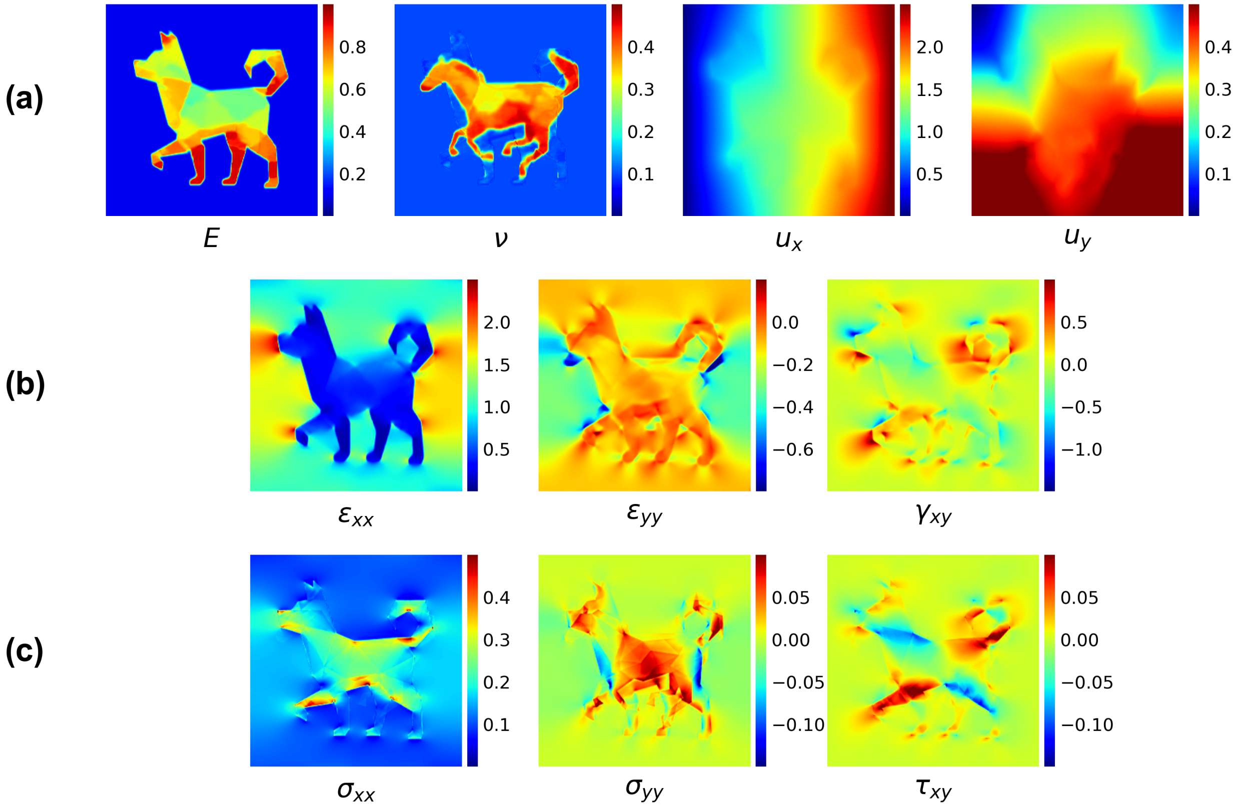

Supplementary Figure S13: The predicted elastic properties based on the dog-shaped distribution of Young's modulus and the horse-shaped distribution of Poisson's ratio. The model uses the noisy displacement field at a signal-to-noise ratio (SNR) of 1000. (a) The predicted Young's modulus field, Poisson's ratio field, and axial displacement field. (b) The predicted strain field (%). (c) The predicted stress field.

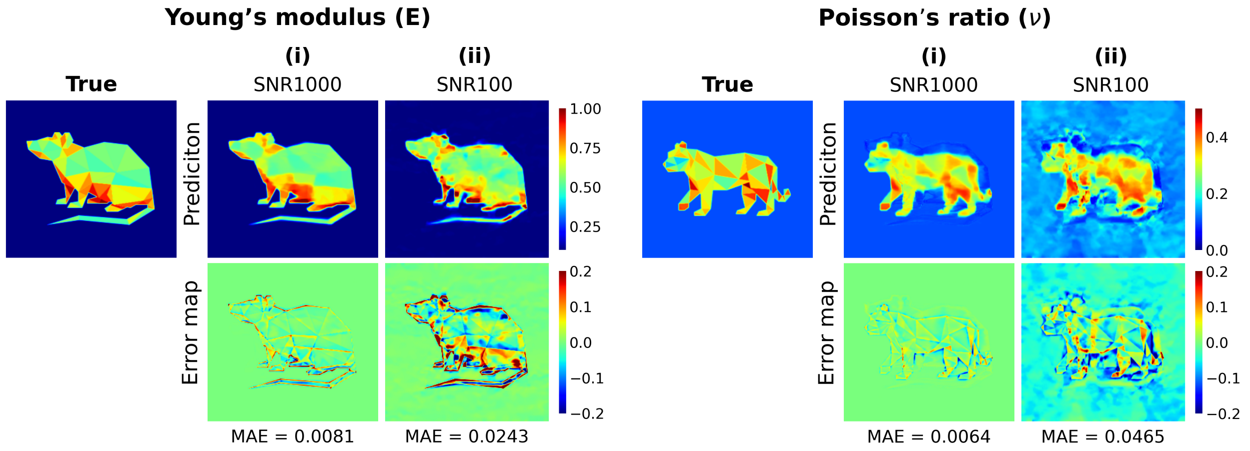

(a) Estimated Young's modulus across noise levels.

(b) Estimated Poisson's ratio across noise levels.

Supplementary Figure S14: Predicted (a) Young's modulus (E) and (b) Poisson's ratio (ν), along with their corresponding error maps, evaluated across varying noise levels (signal-to-noise ratio, SNR) with a dataset where the true spatial distribution of Young's modulus adopts a rat shape and Poisson's ratio a tiger shape. IE-PINN was trained using the same displacement data across two different SNRs: (i) SNR $= 1000$, and (ii) SNR $= 100$.

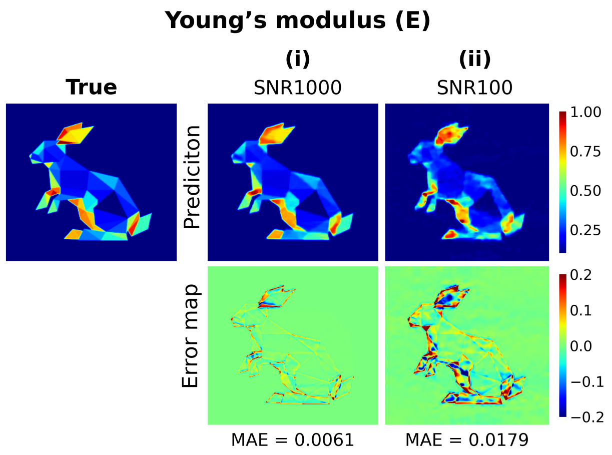

(a) Estimated Young's modulus across noise levels.

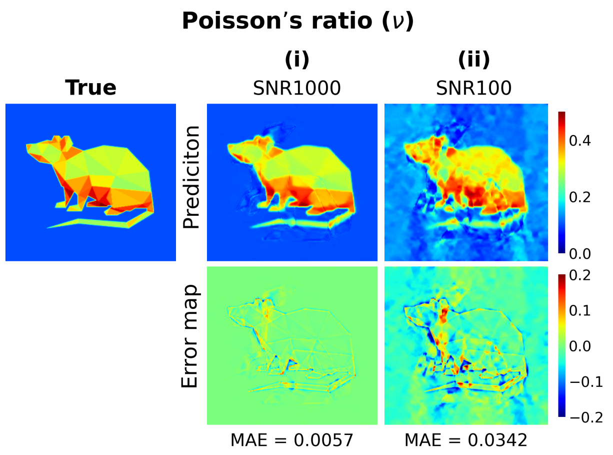

(b) Estimated Poisson's ratio across noise levels.

Supplementary Figure S15: Predicted (a) Young's modulus (E) and (b) Poisson's ratio (ν), along with their corresponding error maps, evaluated across varying noise levels (signal-to-noise ratio, SNR) with a dataset where the true spatial distribution of Young's modulus adopts a rabbit shape and Poisson's ratio a rat shape. IE-PINN was trained using the same displacement data across two different SNRs: (i) SNR = 1000, and (ii) SNR = 100.

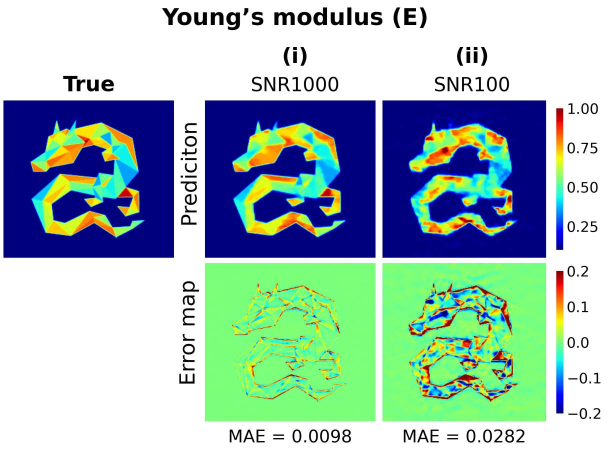

(a) Estimated Young's modulus across noise levels.

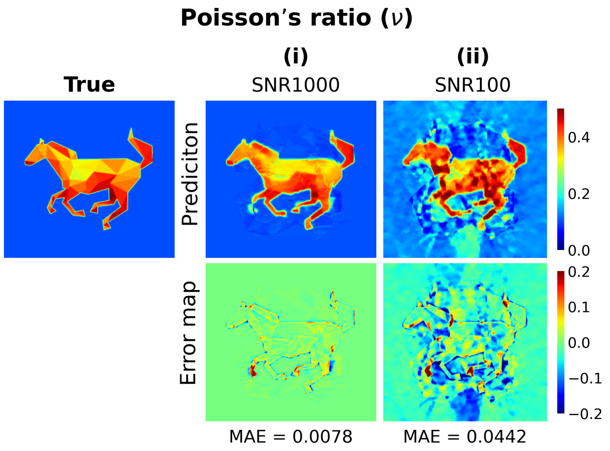

(b) Estimated Poisson's ratio across noise levels.

Supplementary Figure S16: Predicted (a) Young's modulus (E) and (b) Poisson's ratio (ν), along with their corresponding error maps, evaluated across varying noise levels (signal-to-noise ratio, SNR) with a dataset where the true spatial distribution of Young's modulus adopts a dragon shape and Poisson's ratio a horse shape. IE-PINN was trained using the same displacement data across two different SNRs: (i) SNR = 1000, and (ii) SNR = 100.

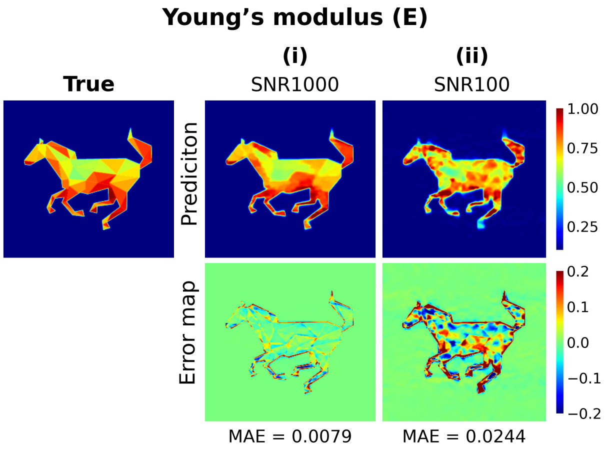

(a) Estimated Young's modulus across noise levels.

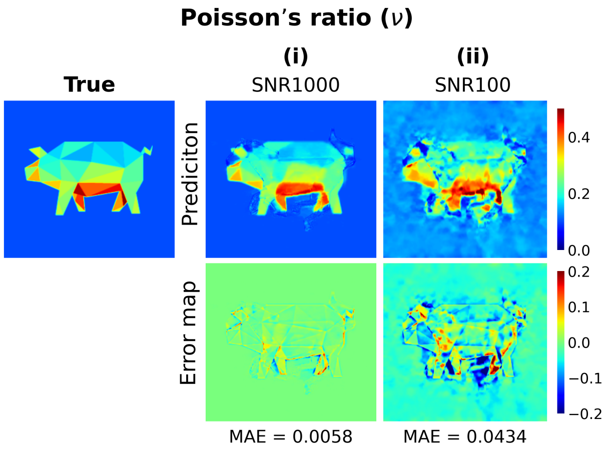

(b) Estimated Poisson's ratio across noise levels.

Supplementary Figure S17: Predicted (a) Young's modulus (E) and (b) Poisson's ratio (ν), along with their corresponding error maps, evaluated across varying noise levels (signal-to-noise ratio, SNR) with a dataset where the true spatial distribution of Young's modulus adopts a horse shape and Poisson's ratio a pig shape. IE-PINN was trained using the same displacement data across two different SNRs: (i) SNR = 1000, and (ii) SNR = 100.

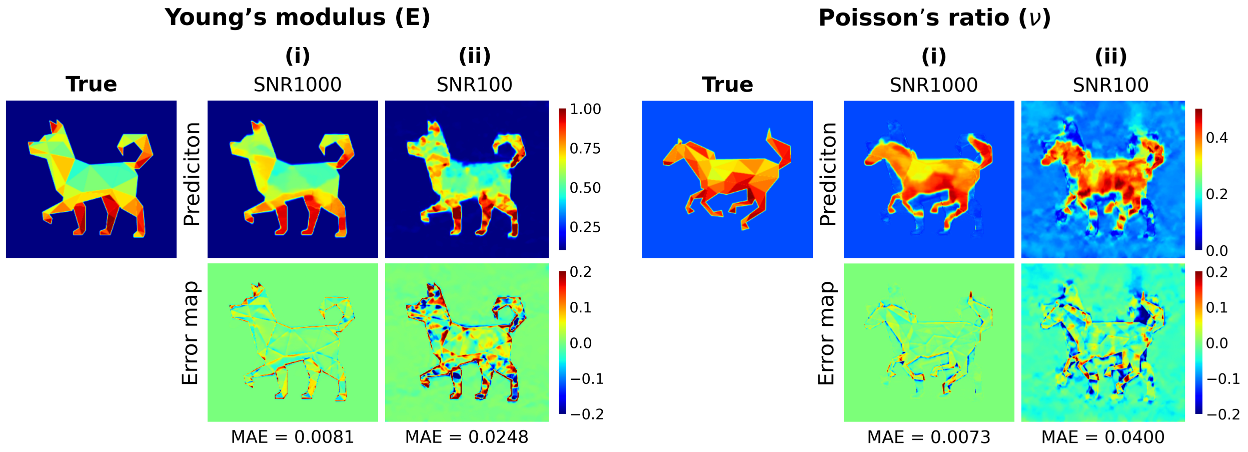

(a) Estimated Young's modulus across noise levels.

(b) Estimated Poisson's ratio across noise levels.

Supplementary Figure S18: Predicted (a) Young's modulus (E) and (b) Poisson's ratio (ν), along with their corresponding error maps, evaluated across varying noise levels (signal-to-noise ratio, SNR) with a dataset where the true spatial distribution of Young's modulus adopts a dog shape and Poisson's ratio a horse shape. IE-PINN was trained using the same displacement data across two different SNRs: (i) SNR = 1000, and (ii) SNR = 100.

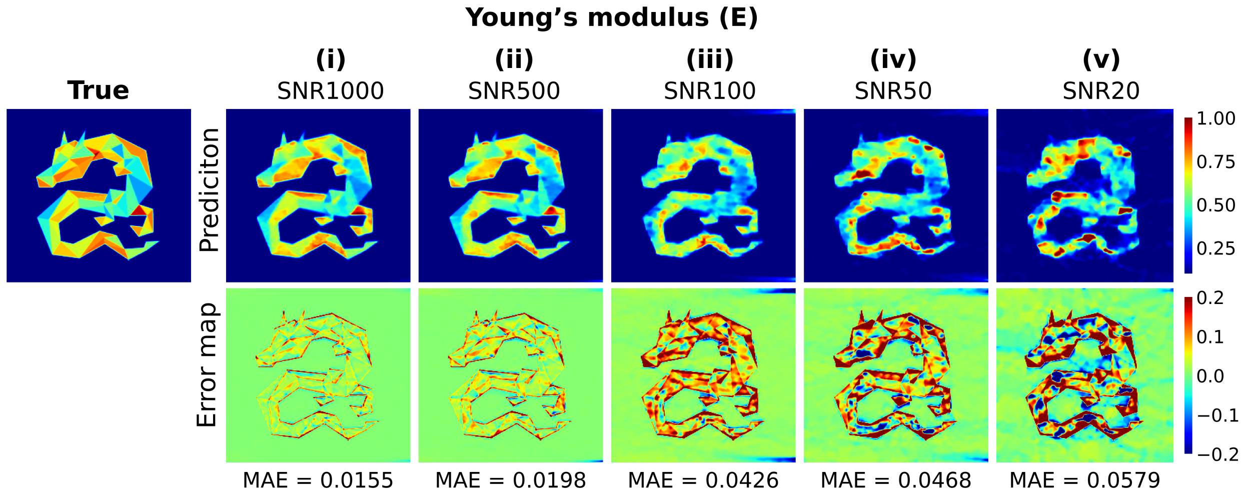

(a) Estimated Young's modulus across spatially dependent structured noise levels.

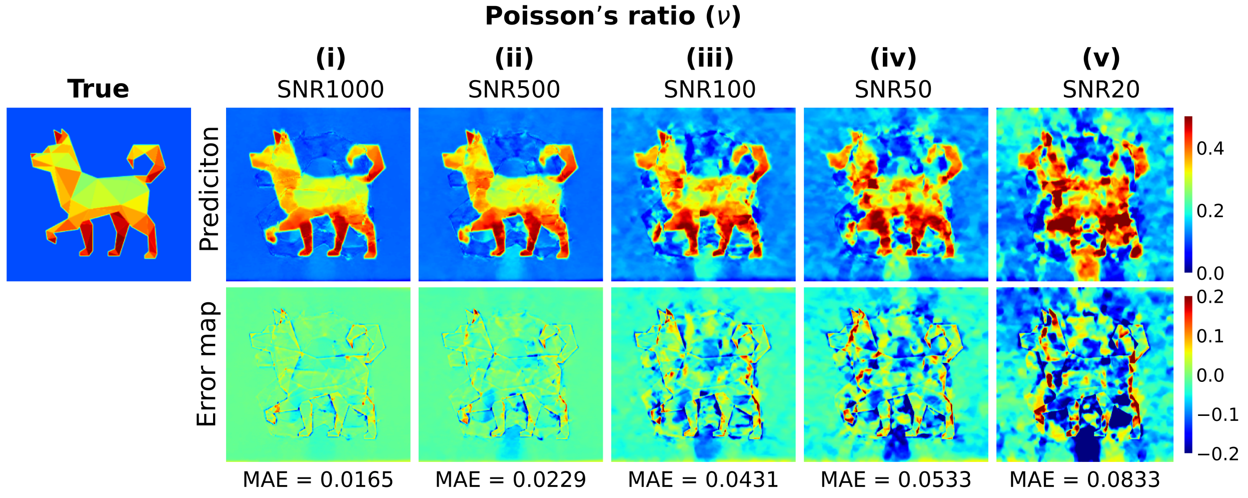

(b) Estimated Poisson's ratio across spatially dependent structured noise levels.

Supplementary Figure S19: Robustness of IE-PINN under spatially dependent structured noisy displacement data. Predicted field of (a) Young's modulus (E) and (b) Poisson's ratio (ν), along with their corresponding error maps, are evaluated across varying noise levels, defined by signal-to-noise ratio (SNR): (i) SNR = 1000, (ii) SNR = 500, (iii) SNR = 100, (iv) SNR = 50 and (v) SNR = 20. IE-PINN was trained using the displacement data across five different SNRs. Although prediction errors increase with higher noise, the model maintains robust performance across all noise levels.

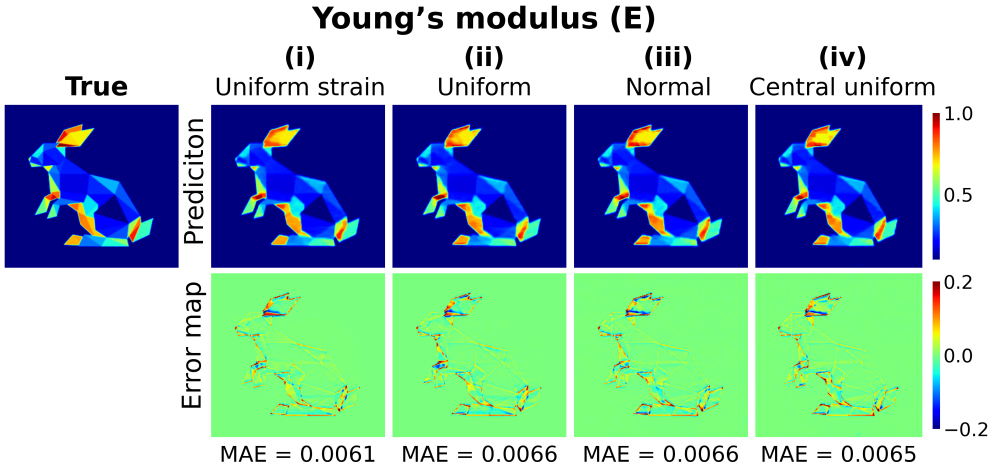

(a) Estimated Young's modulus across boundary loading conditions.

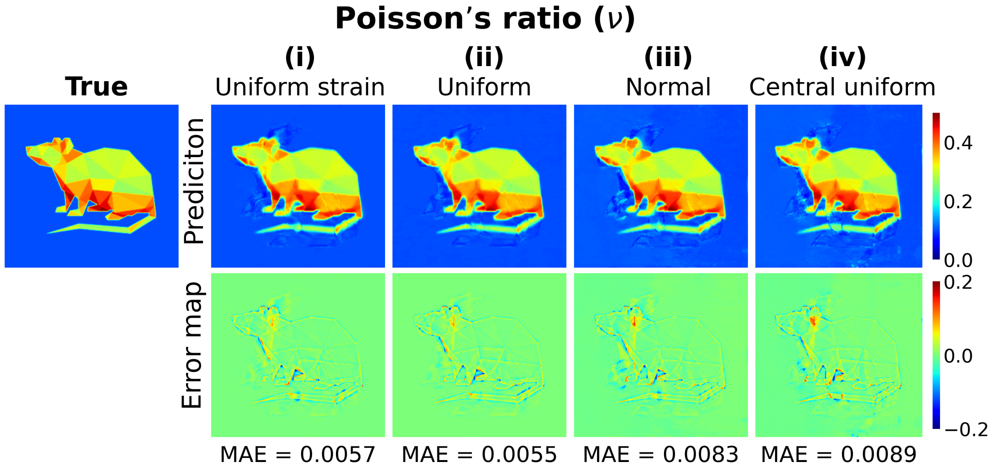

(b) Estimated Poisson's ratio across boundary loading conditions.

Supplementary Figure S20: Predicted (a) Young's modulus (E) and (b) Poisson's ratio ((ν)), along with their corresponding error maps, evaluated under four different boundary loading conditions. The dataset is characterized by a distribution of Young's modulus in the shape of a rabbit and Poisson's ratio in the shape of a rat.

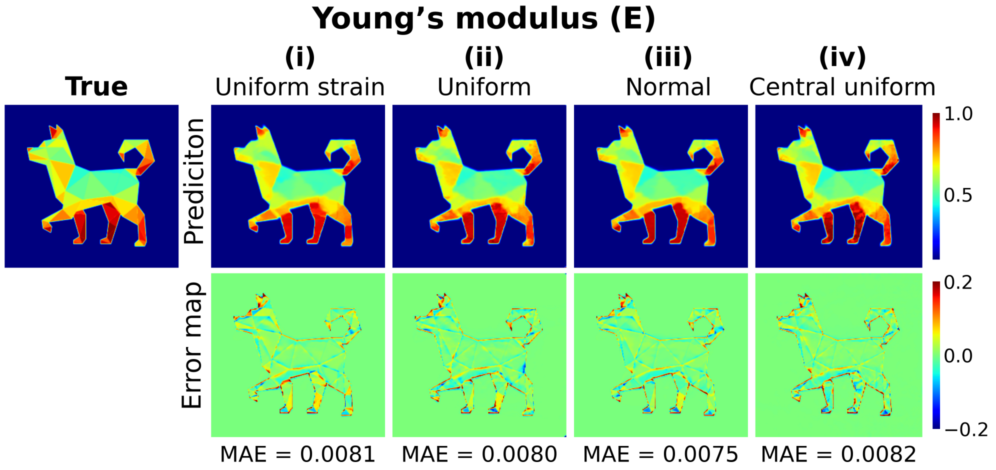

(a) Estimated Young's modulus across boundary loading conditions.

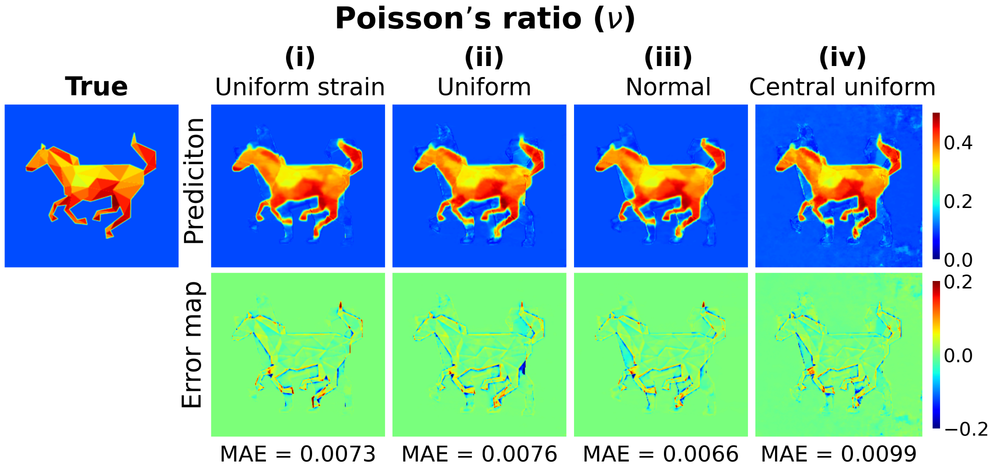

(b) Estimated Poisson's ratio across boundary loading conditions.

Supplementary Figure S21: Predicted (a) Young's modulus (E) and (b) Poisson's ratio ((ν)), along with their corresponding error maps, evaluated under four different boundary loading conditions. The dataset is characterized by a distribution of Young's modulus in the shape of a dog and Poisson's ratio in the shape of a horse.

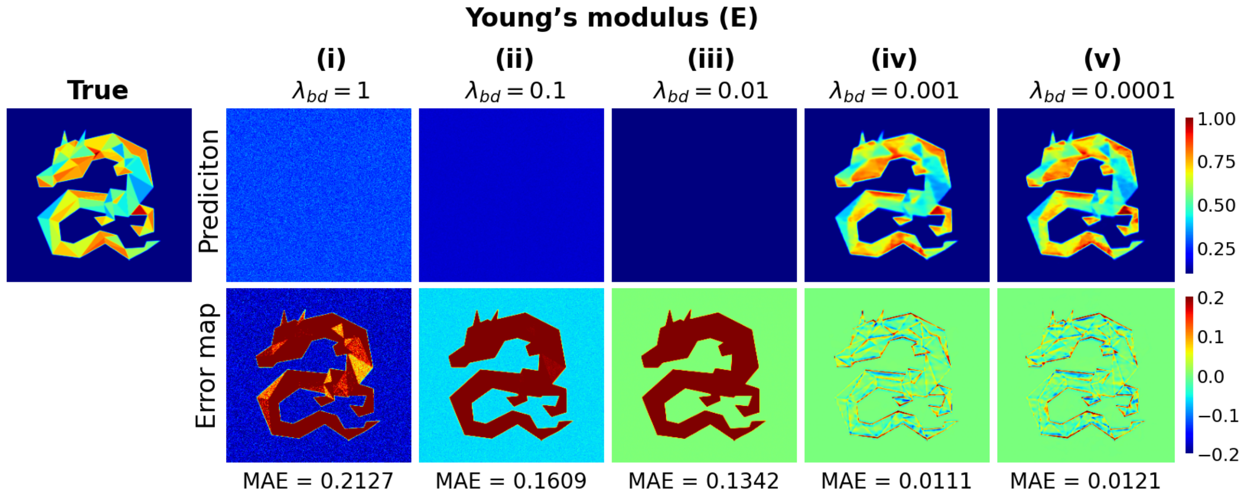

(a) Estimated Young's modulus across noise levels.

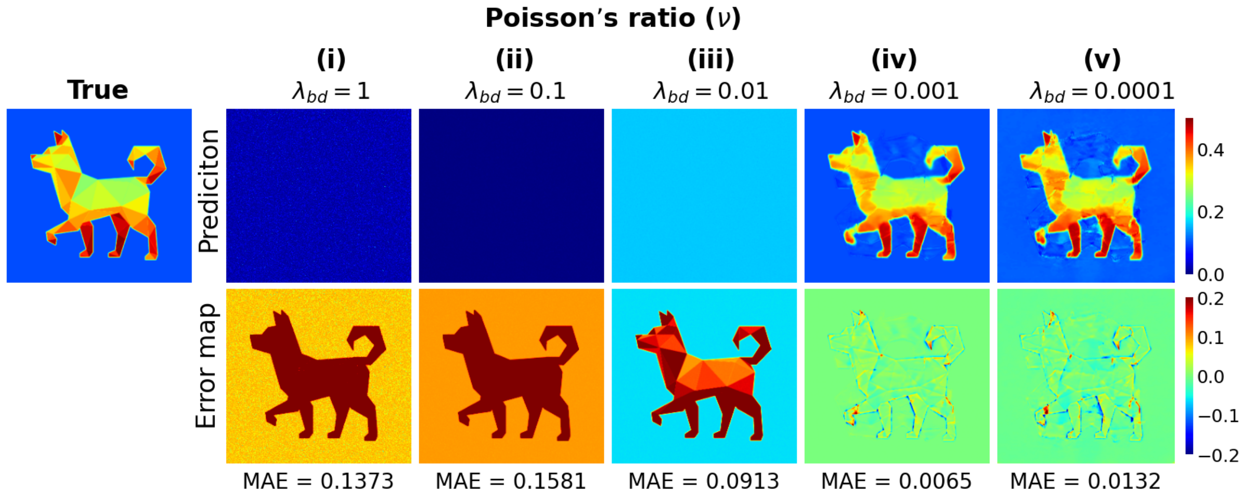

(b) Estimated Poisson's ratio across noise levels.

Supplementary Figure S22: The single-phase approach is sensitive to the loss weight λ_{bd}. When λ_{bd} is not properly chosen, it fails to estimate the elastic properties. Predicted (a) Young's modulus (E) and (b) Poisson's ratio (ν), along with their corresponding error maps, are evaluated across different weights assigned to the boundary condition during single-phase training.

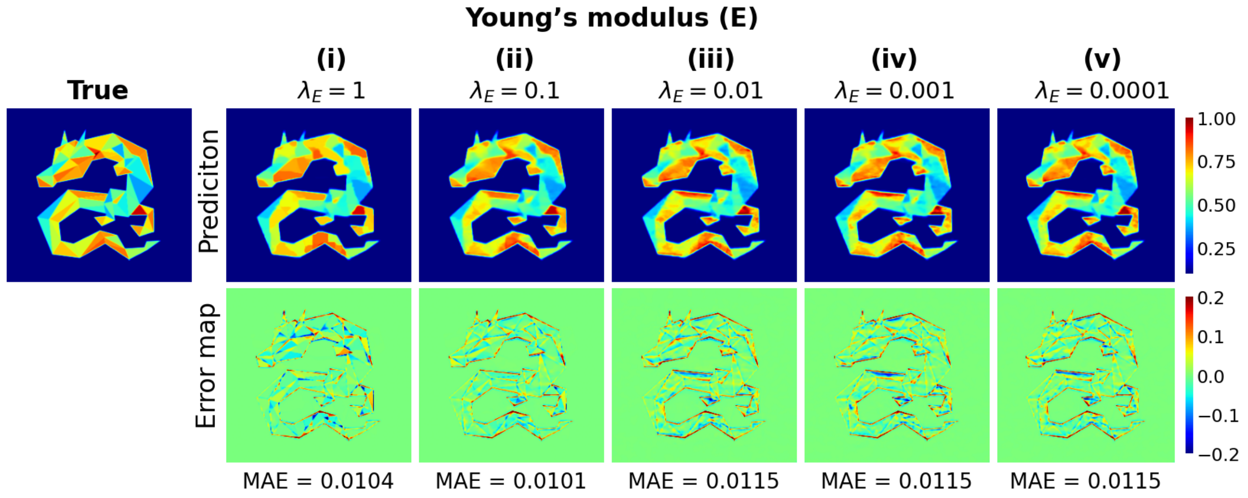

(a) Estimated Young's modulus across noise levels.

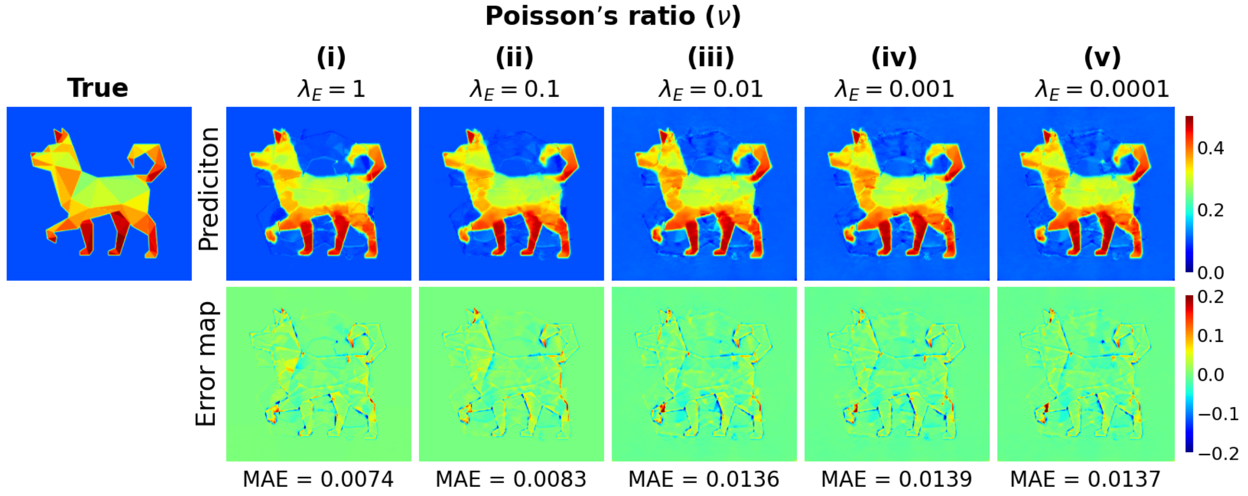

(b) Estimated Poisson's ratio across noise levels.

Supplementary Figure S23: The two-phase approach is stable with respect to the loss weight λ_E. Its performance is not significantly affected by variations in λ_E. Predicted (a) Young's modulus (E) and (b) Poisson's ratio (ν), along with their corresponding error maps, are evaluated across different weights assigned to the mean modulus constraint loss during two-phase training.

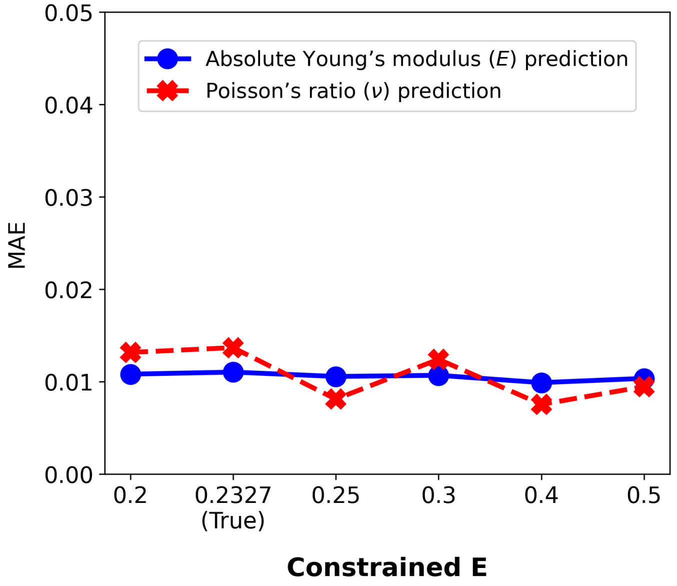

Supplementary Figure S24: Impact of constrained mean Young's modulus to elasticity parameters estimation. The estimation error (Mean Squared Error: MSE) comparison of Young's modulus (E) and Poisson's ratio (ν) estimations of different constrained mean Young's modulus. The model is trained with the Dragon and Dog dataset and is subjected to the measured noisy displacement (SNR1000). Both Young's modulus (E) and Poisson's ratio (ν) estimations are consistently robust regardless of the different values of mean Young's modulus constraints.

Supplementary Tables

Supplementary Table S1: Robustness of estimation accuracy under different constrained mean values of Young's modulus. Estimation error of absolute Young's modulus (E) and Poisson's ratio (ν) under varying constraints on the mean value of Young's modulus. Results are based on the Dragon and Dog datasets, with displacement measurements corrupted by Gaussian noise at a signal-to-noise ratio (SNR) of 1000.

Constrained E	MAE of Relative E	MAE of Absolute E	MAE of ν
0.20	0.0338	0.0108	0.0132
0.2327 (True)	0.0117	0.0110	0.0137
0.25	0.0219	0.0106	0.0081
0.30	0.0689	0.0107	0.0124
0.40	0.1672	0.0099	0.0076
0.50	0.2673	0.0104	0.0095

Supplementary Table S2: Accuracy under varying levels of force measurement error. Prediction error of predicted absolute Young's modulus (E) and Poisson's ratio (ν) under different levels of error in the measured force. Results are based on the Dragon and Dog datasets, using displacement measurements corrupted by Gaussian noise at a signal-to-noise ratio (SNR) of 1000.

Error in measured force	MAE of Absolute E	MAE of ν
-3.0%	0.0130	0.0081
-2.0%	0.0119	0.0081
-1.0%	0.0110	0.0081
-0.5%	0.0106	0.0081
**0% (Baseline)**	**0.0106**	**0.0081**
0.5%	0.0110	0.0081
1.0%	0.0115	0.0081
2.0%	0.0126	0.0081
3.0%	0.0140	0.0081

Supplementary Table S3: Training parameters of IE-PINN

Parameter	Value
Common	
learning rate (α)	10^{-3}
optimizer	Adam
Displacement network architecture	
network structure	fully connected neural network
number of hidden layers	16
number of neurons per layer	128
linearity	SIREN [2]
activation function of output	-
Strain network architecture	
network structure	fully connected neural network
number of hidden layers	16
number of neurons per layer	128
linearity	SIREN [2]
activation function of output	-
Elasticity network architecture	
network structure	fully connected neural network
number of hidden layers	16
number of neurons per layer	128
linearity	SIREN [2]
activation function of output	Softplus function
Weighting scheme	
weight of displacement fitting loss (λ_u)	3
weight of of strain fitting loss (λ_ε)	1
weight of residual of equilibrium loss (λ_r)	1
weight of mean modulus constraint loss (λ_E)	0.1

Supplementary Table S4: Computational resources for IE-PINN

Phase	Elapsed time (h:mm)	Actual usage memory (MB)	Peak usage memory (MB)	Allocated usage memory (MB)
Pretraining stage				
Displacement fitting	0:35	25.96	1089.28	1252.00
Strain fitting	2:25	29.05	2083.42	2248.00
Physic-informed training stage				
Elasticity learning	10:00	33.63	4136.57	4294.00

Supplementary Notes

Supplementary Note S1: Inaccurate Boundary Conditions

Conventional PINNs incorporate boundary conditions into their loss function through point-wise boundary condition residuals.[3] They heavily rely on precise specification of boundary conditions, which are often subject to errors in practice.[4] When the force application region is imprecisely defined (Figure S25 (ii)), the estimated Young's modulus can be significantly degraded.

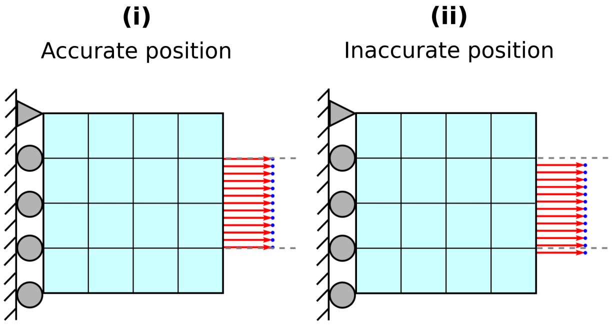

Supplementary Figure S25: Misspecified boundary loading conditions: (i) True loading condition and (ii) deviated loading condition with a slight shift.

For instance, a 2% deviation of the boundary region from the true one in a PINN leads to an 85% increase in estimation error (MAE increases from 0.0093 to 0.0172 in Figure S26(a)(ii)). A 6% deviation results in a 283% increase in error (MAE increases from 0.0094 to 0.0360 in Figure S27(a)(ii). The prediction of the Poisson ratio remains unaffected by the inaccuracies in loading information across both methods. In the IE-PINN framework, the boundary region can be inferred from the surface stress distribution in Phase 1. This inferred boundary region can be applied in Phase 2, enabling an accurate and robust estimation of the elastic properties even when the boundary condition deviates from the true location (Figures S26(a)(i) and S27(a)(i)).

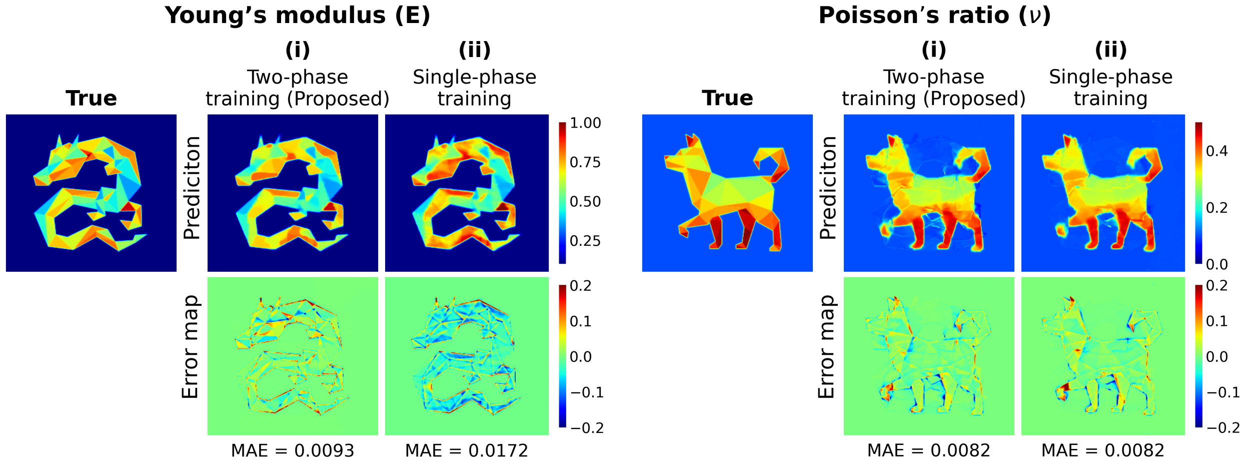

(a) Estimated Young's modulus **(b)** Estimated Poisson's ratio

Supplementary Figure S26: Elasticity estimation under a misspecified loading region with a 2% spatial shift from the true location. (a) Young's modulus: The single-phase approach exhibits an 85% increase in estimation error, whereas the two-phase strategy accurately infers the shifted region and maintains robust performance. (b) Poisson's ratio: its accuracy remains unaffected by the boundary condition

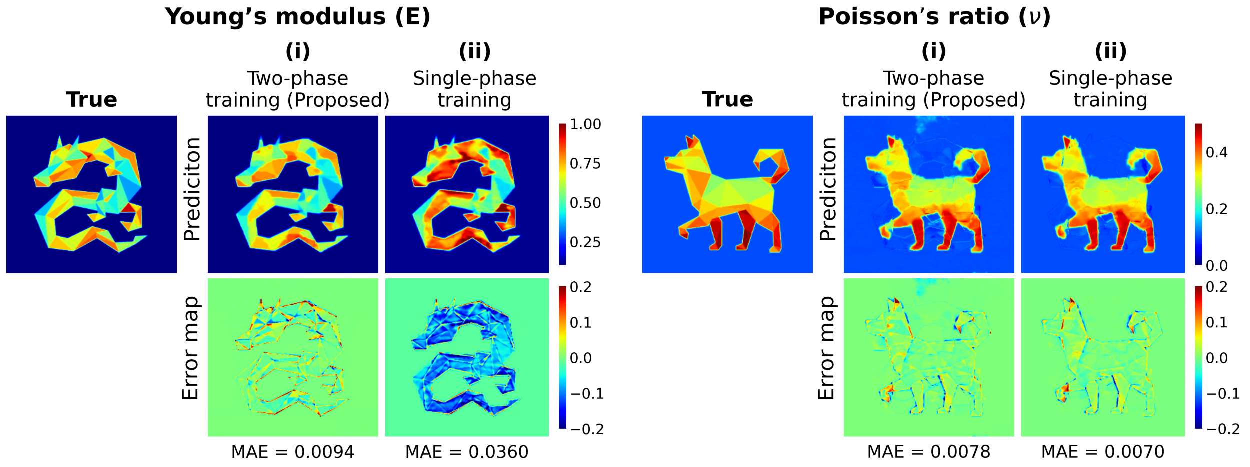

(a) Estimated Young's modulus across different strategies **(b)** Estimated Poisson's ratio across different strategies

Supplementary Figure S27: Elasticity estimation under a misspecified loading region with a 6% spatial shift from the true location. (a) Young's modulus: The single-phase approach exhibits a 283% increase in estimation error, whereas the two-phase strategy accurately infers the shifted region and maintains robust performance. (b) Poisson's ratio: its accuracy remains unaffected by the boundary condition

Supplementary Note S2: Accuracy of Measured Force

In ultrasound imaging, the applied force can be measured by attaching force sensors to the probe.[5, 6]

Elasticity is frequently inferred from mechanical testing, which involves the measurement of applied force and the corresponding deformation, where the applied force is accurately recorded.[7] However, in practical settings of mechanical testing, precise information regarding the distribution and region of the applied force is often unavailable.[8]

The proposed IE-PINN model employs a two-phase estimation strategy to establish the spatial distribution of relative elasticity without relying on boundary information in Phase 1. In Phase 2, the model utilizes only the magnitude of the applied force to calibrate the Young's modulus prediction onto an absolute scale. This approach does not require prior measurement of the stress distribution or the spatial region of force application. Furthermore, the model is capable of inferring both the stress distribution and the region where the force was applied as part of its predictive output, without necessitating additional inputs.

Only the magnitude of the applied force is required for the second-phase calibration of Young's modulus to an absolute scale. This scalar quantity can be directly acquired from the mechanical testing machine. Mechanical testing systems employed across engineering, manufacturing, and scientific domains are subject to standardized calibration protocols such as ISO 7500-1 and ASTM E4.[9] These standards define the procedures for calibration and verification of force measurement. ISO 7500-1 categorizes accuracy classes into 0.5, 1, 2, and 3, which represent permissible maximum relative errors expressed as percentages.[10] In comparison, the ASTM E4 standard requires that the applied force must be measured with an accuracy within ±1%.[11]

The measured force is utilized to compute the absolute scale of Young's modulus as defined:

$$\hat{c} = \frac{F_{\text{applied}}}{\sum_{i=0}^{Y} \hat{\sigma}_{xx}(x_b, y_i)h}$$

Given this relationship, any measurement error in the applied force will directly influence the accuracy of the predicted Young's modulus. The sensitivity of the Young's modulus estimation to errors in force measurements is quantitatively analyzed in Table S2. This analysis shows that although the accuracy of Young's modulus may be marginally affected by force measurement errors, the Poisson's ratio remains unchanged.

Supplementary Note S3: Pretraining Strategy Details

The IE-PINN model was trained for a total of 350,000 iterations (150,000 iterations for pretraining and 200,000 iterations for elasticity estimation). This training process was completed in approximately 13 hours to obtain the most accurate estimation with a deep architecture, following ElastNet.[1] For ease of notation, we define one epoch as equivalent to 1,000 iterations.

The pretraining stage required approximately 3 hours to complete, as shown in Figure S28. Fitting neural networks without PDE residual loss is computationally less intensive than training with the full physics-informed loss. This is because computing the PDE residual loss involves backpropagation through convolution operations, which significantly increases the computational cost. As a result, the pretraining stage facilitates rapid fitting of the displacement and strain networks before incorporating the more computationally expensive components.

After pretraining, the physics-informed training involving the elastic network proceeded at an average rate of approximately 3 minutes per epoch. For the final reported results, we trained the model for 200 epochs, which took about 10 hours. It is important to note that IE-PINN exhibits rapid initial convergence. The model achieves reasonably accurate predictions within the first few epochs and gradually improves the quality of its estimations over time.

Figure S29 illustrates the estimated elastic properties and the corresponding errors at every five epochs. The mean absolute error (MAE) stabilizes by epoch 20, which corresponds to roughly 1 hour of training. At this point, the MAE values for Young's modulus and Poisson's ratio are 0.0132 and 0.0236, respectively. Continued training up to 200 epochs further reduces the MAE to 0.0106 for Young's modulus and 0.0081 for Poisson's ratio. Additionally, the loss trajectories comparing the two strategies are demonstrated in Figure S30.

In many high-impact applications, real-time prediction is not the central requirement; instead, the precision of the recovered elasticity fields is paramount. For example, in ultrasound elastography, precisely identifying anomalous tissue is critical, as errors in clinical decisions can have prohibitive consequences. The reported 13 hours of training time corresponds to the most accurate setting with a deep architecture, following ElastNet.[1] In practice, a favorable trade-off between computational cost and accuracy can be achieved. With a 3-hour pretraining phase followed by 1 hour of physics-informed training, IE-PINN already yields precise estimates of heterogeneous elasticity fields (Figure S29). Furthermore, reducing the network depth from 16 to 4 layers shortens the total training to approximately 1 hour. Although this simplification increases the mean absolute error (by factors of 3.8 for the Young's modulus (E) and 7.9 for the Poisson's ratio (ν) compared to the 13-hour model), the recovered elasticity maps still capture the essential structures (Figure S31).

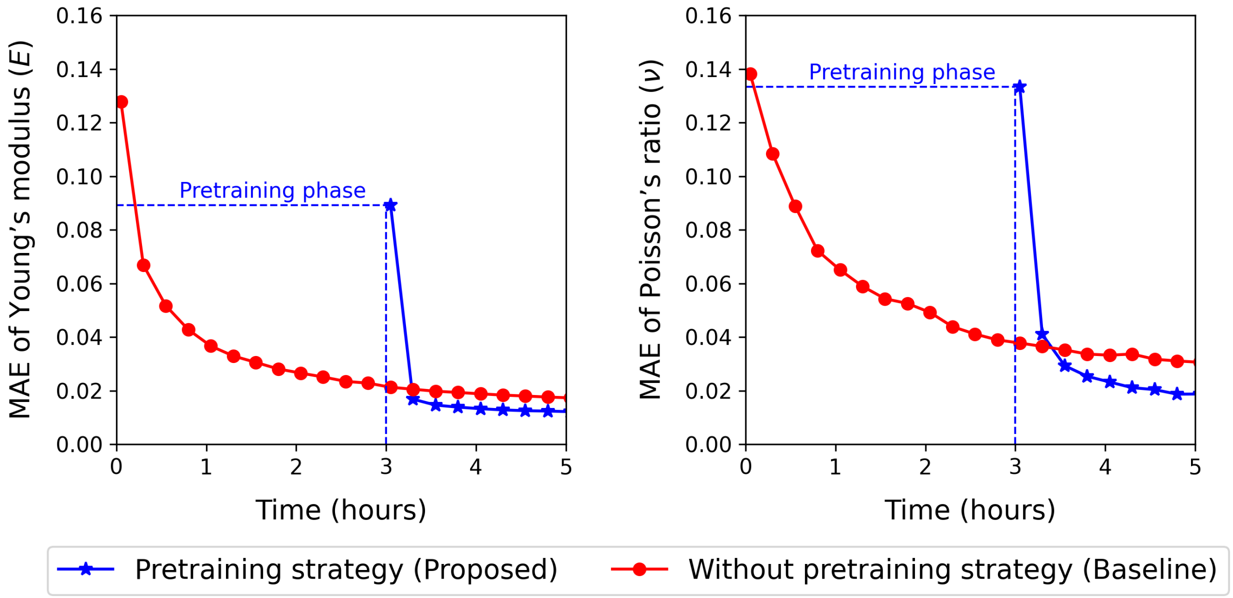

Supplementary Figure S28: The training time and estimation error of predicting Young's modulus (E) and Poisson's ratio (ν) are compared between the two training approaches. After three hours in the pretraining stage, the predictions for elasticity are more accurate when a pretraining strategy is used compared to when it is not, especially as estimation error converges.

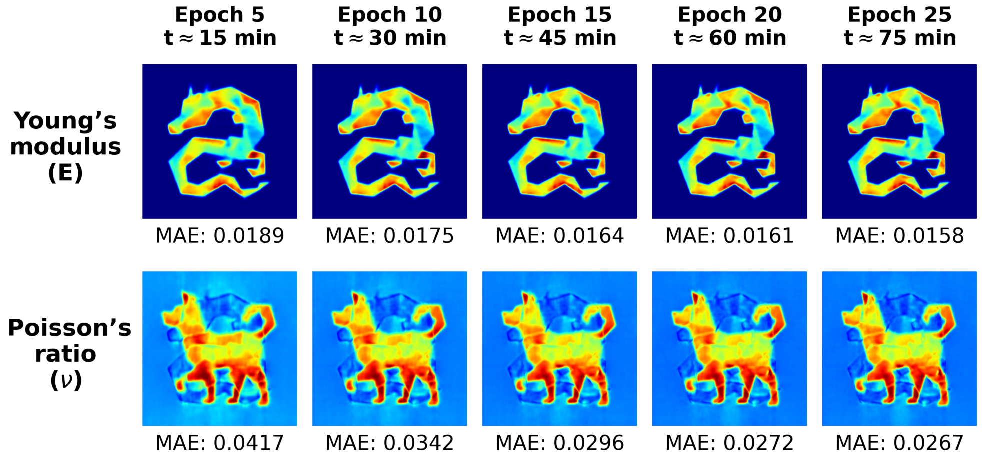

Supplementary Figure S29: Elasticity estimation results during training. The prediction error for Young's modulus (E) and Poisson's ratio (ν) is robust throughout training and progressively improves. The model converges within 20 epochs, with a total training time of 4 hours (3 hours for pretraining and 1 hour for physics-informed elasticity learning).

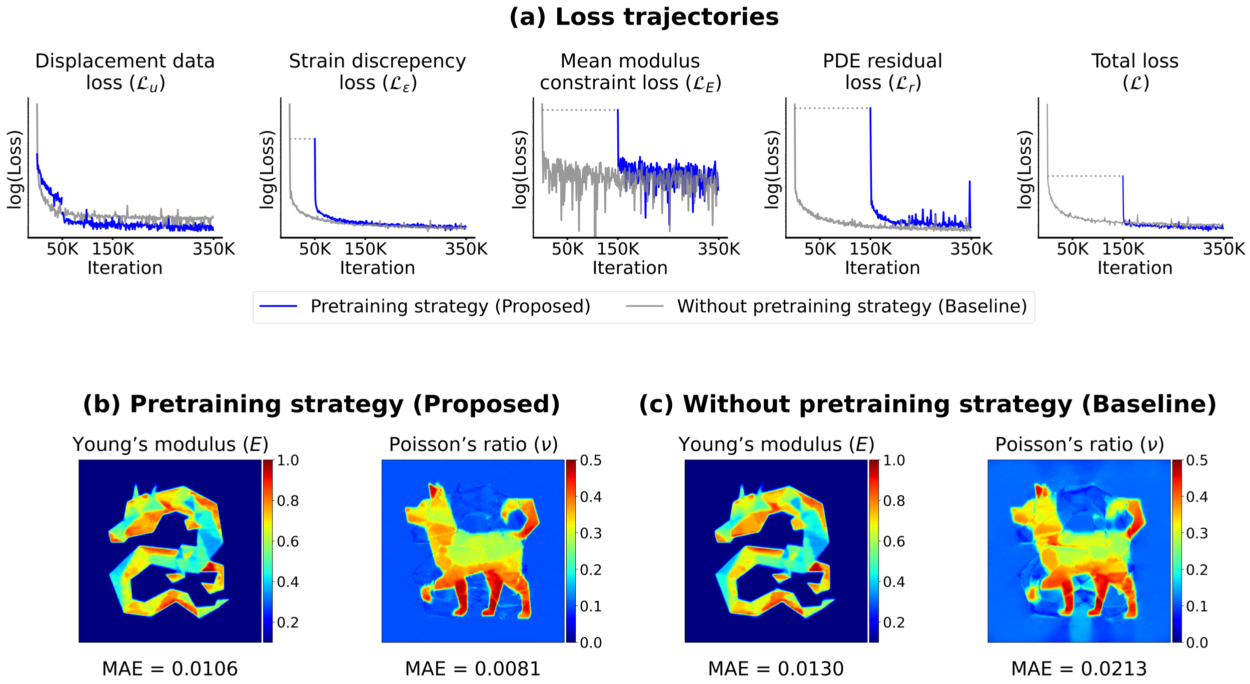

Supplementary Figure S30: Benefits of Pretraining Strategy. Both training strategies were implemented using the same parameters on the same dataset. The results include: (a) Loss trajectories of two different approaches. The proposed model is trained with pretraining: i) 50,000 iterations for displacement network training, ii) 100,000 iterations with strain network (until 150,000 iterations), and iii) 200,000 iterations with entire networks of IE-PINN. Pretraining effectively reduces the total loss function, especially with significantly lower displacement loss. (b) Elasticity predictions obtained from pretraining strategy (Proposed), and (c) Elasticity predictions from simultaneous training of the three-network scheme. The loss trajectories illustrate the trade-offs between the two strategies. While the pretraining strategy can achieve a lower displacement fitting loss, the simultaneous training can achieve a lower PDE residual loss. However, the total loss with the pretraining strategy is significantly better, resulting in more accurate predictions of Young's modulus (E) and Poisson's ratio (ν).

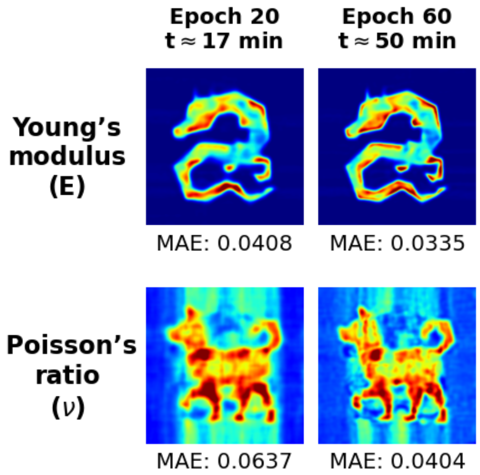

Supplementary Figure S31: Elasticity estimation results during training with a simple neural network architecture. The prediction error for Young's modulus (E) and Poisson's ratio (ν) is robust throughout training and progressively improves. The model can estimate clear patterns of elasticity distribution within 20 epochs, with a total training time of 1 hour (43 minutes for pretraining and 17 minutes for physics-informed elasticity learning).

Supplementary Note S4: Weights Exploration

The weights in this work are explored in two main directions: constant and dynamic weighting factors.

In the constant weight approach, the weights are experimentally optimized with trial and error. We have explored combinations of weights to determine the optimal weight set by varying each weight of each of three loss terms: displacement fitting loss (λ_u), strain fitting loss (λ_ε) and PDE residual loss (λ_r), while fixing the weight to the mean modulus constraint loss at 0.1, We vary the three selected weights with three distinct values: 1, 2, and 3, which are chosen based on preliminary experiments. The training has been run for 100 epochs (100,000 iterations) with pre-training. The estimation error of elasticity prediction of IE-PINN with different weight patterns is shown in Table S5. From the result, we choose the best combination: the $\lambda_u = 3$, $\lambda_\varepsilon = 1$, $\lambda_r = 1$, and $\lambda_E = 0.1$.

Supplementary Table S5: Estimation error across varying weight settings. The mean absolute errors (MAEs) of predicted absolute Young's modulus (E) and Poisson's ratio (ν) with respect to varying weights for each loss term. The dataset of Dragon and Dog pair is used at a signal-to-noise ratio of 1000.

λ_u	λ_ε	λ_r	MAE of Absolute E	MAE of ν
1	1	1	0.0177	0.0243
1	1	2	0.0177	0.0242
1	1	3	0.0203	0.0298
1	2	1	0.0243	0.0412
1	2	2	0.0227	0.0361
1	2	3	0.0250	0.0383
1	3	1	0.0281	0.0580
1	3	2	0.0298	0.0560
1	3	3	0.0318	0.0512
2	1	1	0.0130	0.0154
2	1	2	0.0137	0.0149
2	1	3	0.0168	0.0214
2	2	1	0.0160	0.0256
2	2	2	0.0171	0.0219
2	2	3	0.0177	0.0223
2	3	1	0.0188	0.0359
2	3	2	0.0201	0.0315
2	3	3	0.0207	0.0316
**3**	**1**	**1**	**0.0113**	**0.0097**
3	1	2	0.0129	0.0154
3	1	3	0.0125	0.0141
3	2	1	0.0138	0.0105
3	2	2	0.0142	0.0172
3	2	3	0.0150	0.0179
3	3	1	0.0160	0.0271
3	3	2	0.0170	0.0231
3	3	3	0.0186	0.0295

On the contrary, the dynamic weighting factor is generally applicable to various PINN problems as discussed in the literature.[12, 13] The dynamic weight factor addresses the challenges in selecting the weights of loss terms in PINN training. This approach addresses the inherent challenge of selecting appropriate weights for multiple loss components in PINN training. A widely used strategy involves learning the weight of each loss term simultaneously

with the neural network parameters. The goal is to assign greater weight to loss components that exhibit relatively higher magnitudes or converge more slowly, thereby maintaining a balanced optimization across terms.

We examined two strategies for training loss weights. The first strategy, loss-term adaptive weighting,[12] updates the weights for each loss term of the total loss function. The second strategy, pointwise adaptive weighting,[13] updates the weights individually at each training point for the various loss terms. We conducted an ablation study to compare both strategies against a constant-weighting baseline by fixing the mean modulus constraint weight as a constant. The results indicate that adaptive weighting based on entire loss components does not improve performance, as shown in Figure S32. Similarly, pointwise adaptive weighting fails when applied to individual loss terms, as shown in Figure S33.

In general, the adaptive weighting schemes did not yield better performance than an empirically optimized set of weights. This outcome may result from the fact that the adaptive sampling scheme is also heuristic, relying on the presumed prospect of optimization. It does not guarantee superior performance. Furthermore, pointwise adaptive weighting is known to be susceptible to noise.

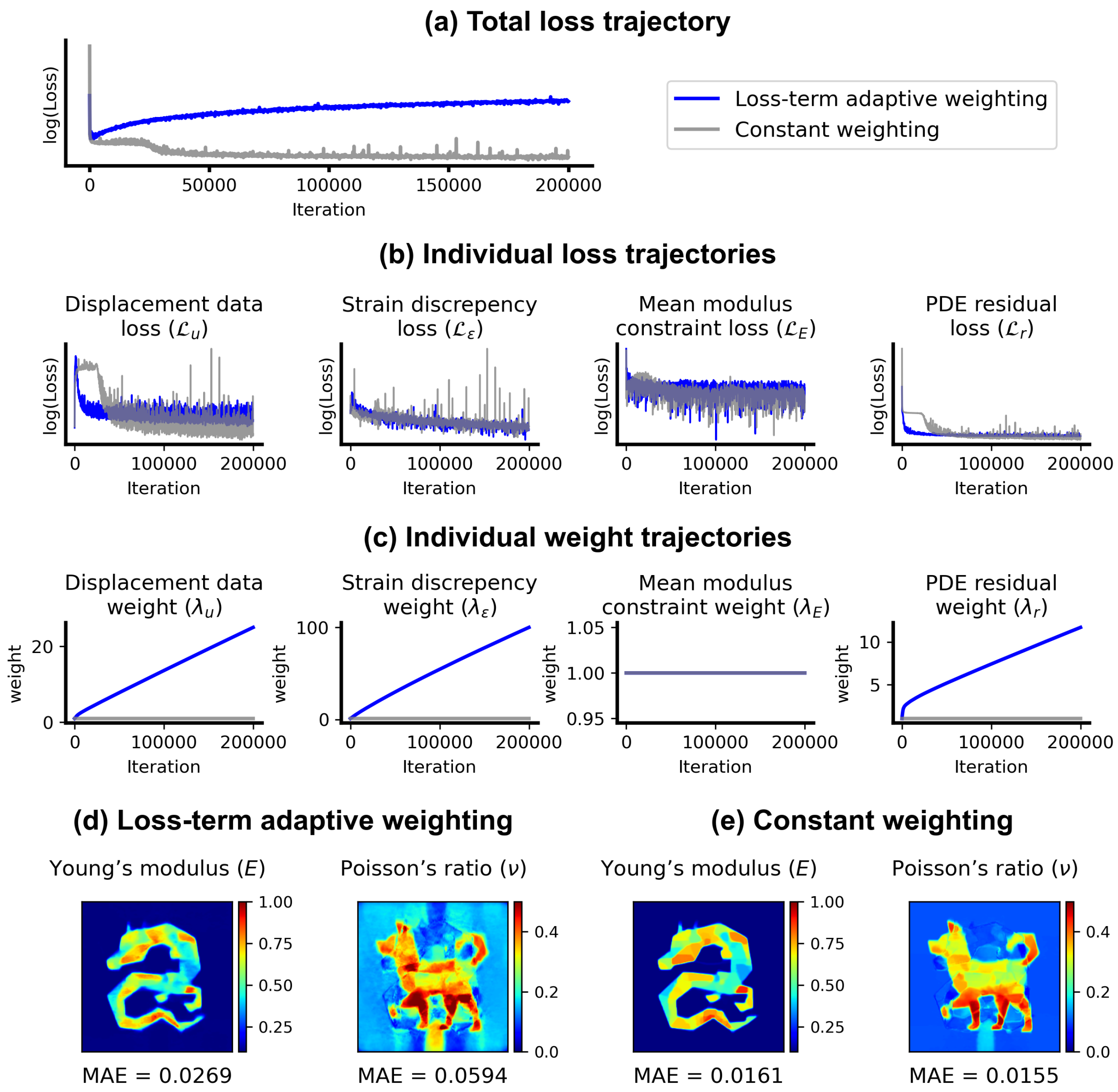

Supplementary Figure S32: Loss trajectories, weight trajectories, and estimated elastic parameters from loss-term adaptive weighting. (a) Total loss trajectories. The loss-term adaptive weighting factor exhibits an increasing pattern of total loss according to an ascending improvement in weight, whereas the constant weighting factor demonstrates a consistent loss improvement during training. (b) Individual loss trajectories. The individual loss trajectories using the adaptive weighting scheme exhibit a similar improvement pattern as the constant weighting scheme across all loss terms; however, the losses are consistently higher with the adaptive scheme. (c) Individual weight trajectories. The adaptive weighting factor shows continuous improvement across all loss terms. Despite the total loss approaching convergence, the individual weight values have not yet converged. (d) Elasticity predictions from the loss-term adaptive weighting factor. (e) Elasticity predictions from a constant weighting factor. The elasticity prediction from the constant weighting factor can yield better accuracy compared with the loss-term adaptive weighting factor.

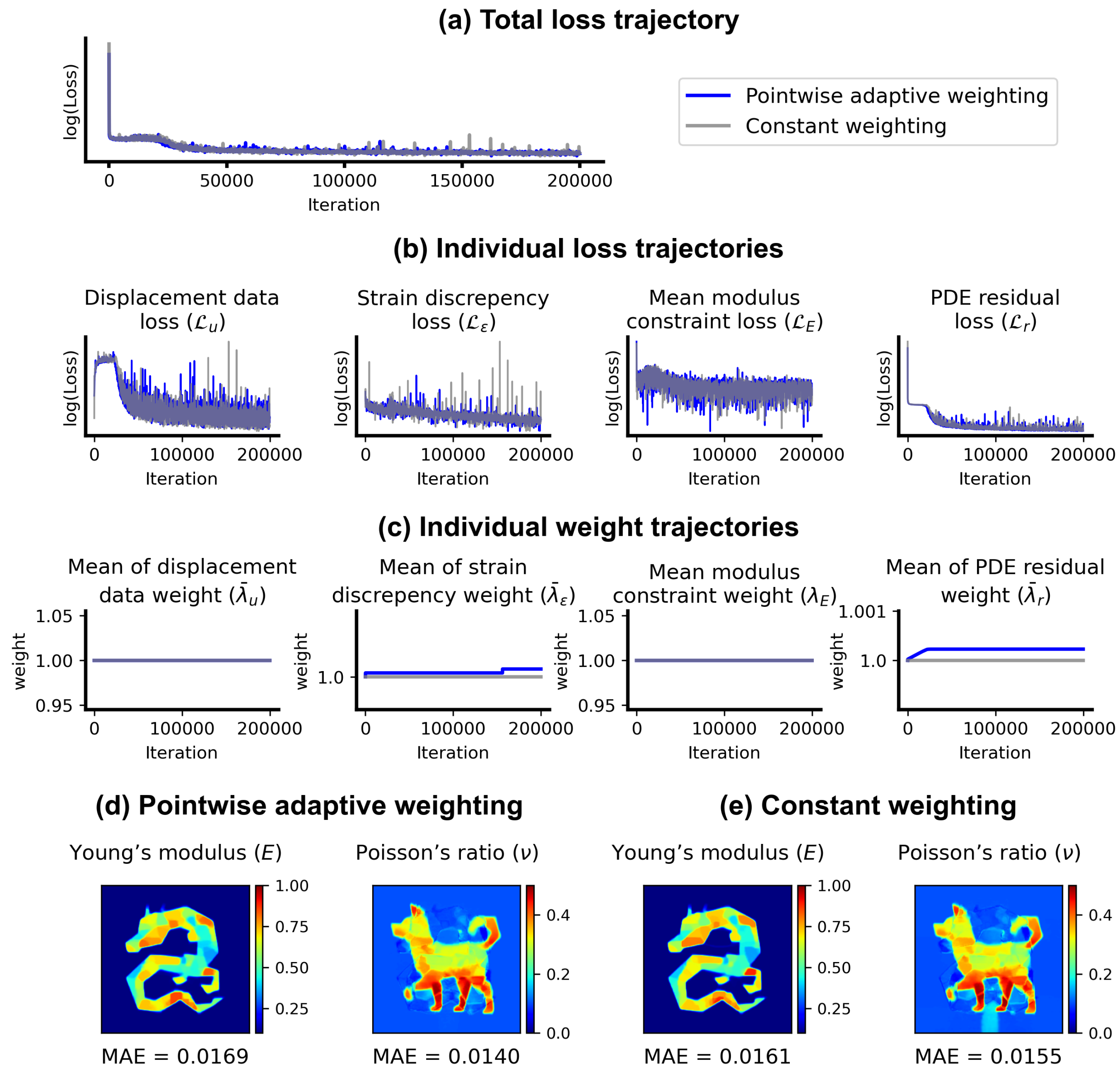

Supplementary Figure S33: Loss trajectories, weight trajectories, and elasticity prediction from pointwise adaptive weighting factor applied to individual training points across different loss components. a) Total loss trajectories. The loss term from the pointwise adaptive weighting factor shows a similar pattern of total loss as the constant weighting factor. (b) Individual loss trajectories on a log scale. All of the loss terms from both strategies exhibit similar loss improvement. (c) Individual weight trajectories. The improvement from the adaptive weighting strategy remains relatively unchanged during training. (d) Elasticity predictions from the pointwise adaptive weighting factor. (e) Elasticity predictions from a constant weighting factor. The pointwise adaptive weighting factor does not significantly improve the accuracy of elasticity prediction compared to the constant weighting factor.

Supplementary Note S5: Loading Boundary Condition

The loading boundary condition refers to the applied force that can cause deformation in an object. This training dataset is generated using a finite element model of an elastic quadrilateral body, where its elasticity is defined by one of 12 zodiac shapes in Young's modulus (E), and Poisson's ratio (ν).[14] The top and bottom boundaries of the object are free. In contrast, the left boundary is fixed to restrict horizontal movement (in the x-direction) to zero, allowing only horizontal movement in the right boundary and vertical movement (in the y-direction). The objective of the simulation is to achieve a displacement of 1% of the body length at the right boundary, which is expected to result in an average normal strain along the x-direction (ε_{xx}) of 1%. Under these conditions, the applied force that defines the loading boundary condition can generally be obtained by the total traction force derived from the stress (calculated from the true boundary strain and elasticity). Since the goal is to produce horizontal displacement at the right boundary, the boundary stress in the x-direction is used to obtain the applied force, as shown in the following equation:

$$F \approx \sum_{i=0}^{Y} \sigma_{xx}(x_b, y_i)h$$

where $\sigma_{xx}(x_b, y_i)$ is true stress in the x-direction at the boundary (x_b), which is obtained from the elastic constitutive relation based on the predefined elasticity and a true strain of 1% of the body length, and h is the width of the spacing between two points, which is equal to one according to the dataset.

References

[1] C. Chen, G. X. Gu, *Adv. Sci.* 2023, **10**, 18.

[2] A. Vaswani, N. Shazeer, N. Parmar, J. Uszkoreit, L. Jones, A. N. Gomez, L. Kaiser, I. Polosukhin, Attention Is All You Need. Preprint at https://arxiv.org/abs/1706.03762 (2017).

[3] M. H. Sadd, *Elasticity: Theory, Applications, and Numerics*, 2nd ed., Elsevier, Amsterdam, 2009.

[4] B. F. Zalewski, R. L. Mullen, R. L. Muhanna, *Eng. Anal. Bound. Elem.* 2009, **33**, 508–513.

[5] T. Schimmoeller, R. Colbrunn, T. Nagle, M. Lobosky, E. E. Neumann, T. M. Owings, B. Landis, J. E. Jelovsek, A. Erdemir, *J. Biomech.* 2019, **83**, 117–124.

[6] A. Kumar, K. M. Kempski Leadingham, M. J. Kerensky, S. Sankar, N. V. Thakor, A. Manbachi, *Front. Med. Technol.* 2023, **5**, 1238129.

[7] H. Varner, T. Cohen, *Soft Matter* 2024, **20**, 9174–9183.

[8] P. E. Barbone, N. H. Gokhale, *Inverse Problems* 2004, **20**, 283–296.

[9] N. Saba, M. Jawaid, M. Sultan, *Mechanical and Physical Testing of Biocomposites, Fibre-Reinforced Composites and Hybrid Composites*, Elsevier, 2019, pp. 1–12.

[10] *International Organization for Standardization, ISO 7500-1:2018 — Metallic materials — Calibration and verification of static uniaxial testing machines*, 2018, Available at: https://www.iso.org/standard/72572.html.

[11] *ASTM International, ASTM E4-20 — Standard Practices for Force Verification of Testing Machines*, 2021, Available at: https://store.astm.org/e0004-20.html.

[12] D. Liu, Y. Wang, *Neural Networks* 2021, **136**, 112–125.

[13] L. D. McClenney, U. M. Braga-Neto, *J. Comput. Phys.* 2023, **474**, 111722.

[14] C. Chen, G. X. Gu, *Proc. Natl. Acad. Sci. USA* 2021, **118**, 31.
